# Twenty-four new species of *Aleiodes* Wesmael from the eastern Andes of Ecuador with associated biological information (Hymenoptera, Braconidae, Rogadinae)

**DOI:** 10.3897/zookeys.405.7402

**Published:** 2014-04-28

**Authors:** Eduardo Mitio Shimbori, Scott Richard Shaw

**Affiliations:** 1 Universidade Federal de São Carlos, Departamento de Ecologia e Biologia Evolutiva, Rod. Washington Luís, km 235, CEP 13565-905, São Carlos, SP, Brazil; 2 University of Wyoming, Department of Ecosystem Science and Management (3354), 1000 E. University Avenue, Laramie, WY 82071 USA

**Keywords:** Taxonomy, biology, rearing, caterpillar, Napo Province, Yanayacu

## Abstract

*Aleiodes* Wesmael is the most diverse rogadine genus worldwide, with specialized koinobiont endoparasitic development in Lepidoptera caterpillars resulting in mummification of the host remains. This paper focuses on describing new *Aleiodes* species from the Yanayacu Biological Station, with special interest in those with biological information. We describe 24 new species (*Aleiodes albidactyl* **sp. n.**, *Aleiodes albigena* **sp. n.**, *Aleiodes albiviria* **sp. n.**, *Aleiodes bimaculatus* **sp. n.**, *Aleiodes cacuangoi* **sp. n.**, *Aleiodes colberti* **sp. n.**, *Aleiodes delicatus* **sp. n.**, *Aleiodes dyeri* **sp. n.**, *Aleiodes elleni* **sp. n.**, *Aleiodes falloni* **sp. n.**, *Aleiodes frosti* **sp. n.**, *Aleiodes kingmani* **sp. n.**, *Aleiodes longikeros* **sp. n.**, *Aleiodes luteosicarius* **sp. n.**, *Aleiodes marilynae* **sp. n.**, *Aleiodes mirandae* **sp. n.**, *Aleiodes napo* **sp. n.**, *Aleiodes nubicola* **sp. n.**, *Aleiodes onyx* **sp. n.**, *Aleiodes shakirae* **sp. n.**, *Aleiodes stewarti* **sp. n.**, *Aleiodes townsendi* **sp. n.**, *Aleiodes tzantza* **sp. n.**, and *Aleiodes yanayacu* **sp. n.**) from Napo Province in Ecuador, 16 of which were reared from host caterpillars. With these results 89 species of Neotropical *Aleiodes* are now known, with 41 of them having host records. The most commonly reared species were in the *circumscriptus/gastritor* species-group, and mostly associated with Geometridae hosts (six of ten species). Three species of *seriatus* species-group, in contrast, were each reared from a different family. One of these species (i.e. *A. frosti* **sp. n.**), reared from Notodontidae, cuts a posterior radial opening in the mummy for emergence, a unique behavior in *Aleiodes*, recorded here for the first time. *A. luteosicarius* **sp. n.** is the first described species from Ecuador in the *pallidator* species-group. Differing from previously described *pallidator* species, which attack only Lymantriinae larvae, *A. luteosicarius* **sp. n.** attacks several species of Arctiinae larvae, being both subfamilies within Erebidae with densely setose caterpillars. We also describe new species of the *gressitti* and *pulchripes* species-groups.

## Introduction

*Aleiodes* is the most common and speciose rogadine braconid genus worldwide. Species richness estimations have changed dramatically in last decade. In the Taxapad catalog (Yu et al. 2012) 431 described species are recorded, but Butcher et al. (2012), in a single work published after that catalog, described 179 new species from Thailand. Those authors estimated the *Aleiodes* fauna of Thailand alone to include more than 400 species, pushing the diversity of *Aleiodes* to a much higher level than previously estimated, especially for the tropical fauna, previously considered not as rich as the Holarctic (S. Shaw 2006). Quicke (2012) discussed evidence suggesting a greatly unknown tropical Ichneumonoidea fauna, where the lack of studies on small body sized groups and highly speciose genera are the main reasons for the underestimation. For Neotropical *Aleiodes*, Delfin–Gonzalez and Wharton (2002) estimated about 200 undescribed species, but in light of these recent works, this number is also likely to be much higher (in addition, for example, there are at least 160 undescribed species from Brazil. EMS, previously unpublished data). Prior to this study, there are 65 described species of *Aleiodes* in Neotropical region, 25 of them with host records (Fortier 2009, Townsend and Shaw 2009, Shimbori and Penteado–Dias 2011).

The first division of *Aleiodes* into species-groups (S. Shaw et al. 1997) accounts for fifteen different groups. After phylogenetic analyses three additional groups were proposed (Fortier and Shaw 1999). Townsend and Shaw (2009) found the species of the closely-related *gastritor* and *circumscriptus* groups in Ecuador to be difficult to separate, and recommended treating these as one single group for Neotropical fauna. Although the existing species-group system presents some limitations when applied to Neotropical fauna (Townsend and Shaw 2009), it provides a working framework to investigate this diverse group and to improve our taxonomic knowledge. Most species-groups are represented in Neotropical Region, except for the *compressor*, *praetor*, *procerus*, *rugulosus*, *ufei* and *unipunctator* groups (S. Shaw et al. 1997, S. Shaw et al. 1998a, Marsh and S. Shaw 1998, S. Shaw et al. 1998b, Marsh and S. Shaw 1999, Marsh and S. Shaw 2001, Marsh and S. Shaw 2003, S. Shaw et al. 2006, S. Shaw et al. 2013), being the *pallidator* species-group represented only by undescribed species in Neotropical Region. Based on undescribed material from Neotropical collections, the *seriatus* species-group is the most diverse group in this region, followed by *circumscriptus*/*gastritor* species-group.

Rogadinae is a cosmopolitan subfamily of koinobiont endoparasitoids of Lepidoptera (M. Shaw 1983, M. Shaw and Huddleston 1991, M. Shaw 1994, S. Shaw 1997), currently divided in five tribes: Stiriopini, Clinocentrini, Yeliconinae, Rogadini and Aleiodini (=*Aleiodes*+*Heterogamus*) (van Achterberg 1995, Zaldívar–Riverón et al. 2008), the later containing *Aleiodes*, the most speciose genus with about 612 described species of the current 1,141 named Rogadinae species (Yu et al. 2012, Butcher et al. 2012, S. Shaw et al. 2013). All Rogadinae induce the hardening of the host larva before pupation, producing the so-called “mummy,” with the mummification of the host larva been considered the only biological synapomorphy of this subfamily (van Achterberg 1995). *Aleiodes* is known to attack almost exclusively exposed-feeding macrolepidepterans, especially the superfamilies Noctuoidea and Geometroidea, and to a lesser extent, Sphingoidea and Papilionoidea (S. Shaw et al. 1997, S. Shaw 2006). A few exceptions include some exposed-feeding microlepidopterans (e.g. Zygaenidae) and, in rare cases, such as *Aleiodes compressor* (Herrich–Schäffer, 1838), macrolepidopterans with semi-concealed habit (M. Shaw 2002). *Aleiodes* commonly attacks second and third instar caterpillars, but first instar records are known, though less successful (M. Shaw 1983). The host is mummified in later instars, and within the hardened skin of the host the parasitoid pupates and eventually emerges through a hole on posterior region of the mummy.

The present work presents descriptions of 24 *Aleiodes* new species, 16 of which with information on biology and photographs of the mummified host larvae. These comprise mostly exposed-feeding host records, with the parasitoids attacking hosts living in small trees and bushes, rather than herbaceous plants near ground level, or higher in the canopy.

## Methods

Specimens for this study were collected during the *Caterpillars and Parasitoids of the Eastern Andes of Ecuador* project (Miller and Dyer 2009), and reared at the Yanayacu Biological Station and Center for Creative Studies (YBS). The YBS is situated on the northeastern slope of the Andes in Napo Province, Ecuador, the watershed streams at the YBS flow to the Amazon basin through the Río Napo, a major tributary of the Amazon River (S. Shaw 2012). The reserve comprises both primary- and secondary-growth montane forests (Miller and Dyer 2009). The plant community at Yanayacu is very diverse and complex, comprising species from at least 76 families (Rab Green et al. 2011).

Specimens were sampled using varied methods including yellow pan trap, Malaise traps situated on the ground and suspended in the canopy, Maxi net, hand collected with aspirators at a light sheet, ultraviolet light trap (= U.V. light trap or black light trap), and during daylight off vegetation with net or vials. Most of the specimens were reared from caterpillars (Greeney 2007), from 2007 to 2013. Caterpillars were sampled by walking through various habitats using two different methods: by hand collecting after inspecting herbs, shrubs and trees up to a height of approximately 2.5 m; or beating plants over a white cloth square of 1×1 m size. Caterpillars were collected in clear plastic bags with their food plant, assigned identification codes, and transported to the rearing shed at YBS. Reared parasitoid specimens are associated with the voucher number of the respective host larva (e.g. YY-00000). Caterpillars and host plants were identified and recorded. Rearing took place in plastic bags in an open-air shelter with ambient temperatures and natural day length. Frass and decaying plant material were removed every other day and new plant material was provided as necessary. While cleaning out the bags, the caterpillars were inspected to note the date of caterpillar pupation or date of parasitoid pupation. Parasitoid pupae were inspected daily for emergence. All emerging adult parasitoids were kept with the original code given to the caterpillar to preserve host data. The parasitoids were preserved in alcohol and transferred to the University of Wyoming where they were dried and point mounted for identification.

Type specimens are deposited at University of Wyoming Insect Museum, University of Wyoming, Laramie, Wyoming, U.S.A. (UWIM). Additional specimens from Canadian National Collection, Ottawa, Canada (CNC) were added whenever appropriate to the scope of this work.

All the species proposed here are satisfactorily distinguished by morphological features. Nevertheless, for some of species, a comparison of ribosomal DNA sequences in gene COI by Donald Quicke was helpful in the definition of species limits.

Because of the supporting grant funding, this work focused on species sampled at the YBS. Some paratypes outside the boundaries of this refuge were included, but only if at least one type specimen is from YBS. All specimens included in this project are from the eastern face of the Andes Mountains, in Napo Province. The altitude of sampling sites ranged from 2,000 to 2,800 meters. In previous work on *Aleiodes* from Napo Province by Townsend and Shaw (2009) the altitudinal range was from 1,383 to 2,837m. Most of additional material is also from Napo province, with altitude ranging from 1,700 to 2,000 meters; only two type specimens are from lowland Manabí Province, at 400 meters elevation.

For recognition of subfamily Rogadinae see van Achterberg (1993) and Sharkey (1997). The definition of *Aleiodes* follows that of van Achterberg (1991) and S. Shaw (1993). Species-group definitions follow S. Shaw et al. (1997), Fortier and S. Shaw (1999) and Townsend and Shaw (2009). Terminology follows Sharkey and Wharton (1997) and S. Shaw et al. (1997). Microsculpture terminology follows that of Harris (1979). Wing vein terminology follows the system adopted by Sharkey and Wharton (1997).

We present descriptions for 24 new species from Northeastern Andes in Ecuador. Along with the description of the new species, we provide summarized taxonomic information on the nine previously described species from the same region (Townsend and Shaw 2009), and also a set of additional characters, not discussed in the original description. New biological information for these species is presented whenever available. Images were captured with a 3MP Leica video camera and a Leica M205C stereomicroscope running Leica Application Suite (LAS) software, and focus-stacked using the same software. Some minor adjustments in images and plate preparation were performed in Adobe Photoshop version CS6. Pictures of caterpillars and host mummies before parasitoid emergence were taken, at the rearing site, by Wilmer Rosendo Simbaña, José Arturo Simbaña and Luis Alberto Salagaje.

### Key to species of *Aleiodes* from eastern Andes of Ecuador

**Table:** 

1	Occipital and epicnemial carina entirely absent (Figure 70)	2
–	Occipital carina present, at least laterally (Figures 46, 85); epicnemial carina present	3
2(1)	Head mostly black and granulate, rugose below antenna (Figure 2), pronotum and propleuron black; malar space 1/2 eye height (Figure 2)	*Aleiodes capillosus* Townsend, 2009
–	Head mostly white to light yellow and smooth, pronotum and propleuron yellow (Figures 69, 70); malar space longer, nearly as long as eye height (Figure 3)	*Aleiodes marilynae* sp. n.
3(2)	Apex of hind tibia with a row of flat setae along inner margin forming a distinct fringe (Figures 4, 20)	4
–	Apex of hind tibia without a row of flat setae along inner margin, setae may be present but not flattened and not forming a distinct fringe	12
4(3)	Occipital carina weak or absent at vertex (Figure 19)	5
–	Occipital carina complete and well-defined at vertex (Figures 35, 43)	10
5(4)	Mesopleuron yellow, sometimes with antero-dorsal corner brown to black (Figures 54); propodeum mostly uniformly colored, without strongly contrasting colors; fore wing vein 1CUa shorter than or equal to length of vein 1CUb (Figure 21)	6
–	Mesopleuron black with a posterior white thumb-like marking (Figure 4); propodeum black anteriorly with white spot posteriorly (Figure 6); vein 1CUa 1.5× longer than vein 1CUb (Figure 5)	*Aleiodes albidactyl* sp. n.
6(5)	Ocell–ocular distance about equal to diameter of lateral ocellus (Figure 19); metasomal tergum 1 at most 1.4× longer than its apical width; species with known biology with normal emergence behavior, cutting a postero-dorsal exit hole in the mummified host caterpillar (Figures 40, 57)	7
–	Ocelli larger, ocell–ocular distance 1/2 diameter of lateral ocellus; metasomal tergum 1 unusually long and narrow, more than 2× longer than its apical width (Figure 47); species with a unique emergence behavior, cutting the whole posterior tip away from the mummified host caterpillar (Figure 51)	*Aleiodes frosti* sp. n.
7(6)	Metasomal terga mostly black to dark brown (Figures 38, 54), sometimes lighter posteriorly or with light spots medially; flagellum dark brown to black; wings hyaline or weakly infuscate	8
–	Metasoma entirely orangish yellow; flagellum black with white band medially (Figure 18); wing membrane tinged honey yellow	*Aleiodes albiviria* sp. n.
8(7)	Pronotum and mesonotum honey yellow; hind coxa bicolored, black apically and white basally (Figures 38, 54)	9
–	Pronotum and mesonotum black except light brown square postero-medially on mesoscutum, hind coxa light brown	*Aleiodes greeneyi* Townsend, 2009
9(8)	Propodeum and metapleuron black (Figure 54); fore wing vein 1M strongly curved basally (as in Figure 120); hind wing vein 1M more than 2.0× longer than vein r-m, vein m-cu absent	*Aleiodes longikeros* sp. n.
–	Propodeum and metapleuron pale yellow (Figure 38); fore wing vein 1M weakly curved (as in Figure 21); hind wing vein 1M shorter than vein r-m, vein m-cu present and distinctly antefurcal to r-m (as in Figure 83)	*Aleiodes dyeri* sp. n.
10(4)	Mesonotum dark brown (Figure 34); ocelli extremely small, ocell–ocular distance 2× ocellus width (Figure 35)	*Aleiodes delicatus* sp. n.
–	Mesonotum orange (Figures 41, 82); ocelli larger, ocell–ocular roughly as long as ocellus width (Figure 43)	11
11(10)	Apical 2/3 of hind coxa, metanotum, propodeum, metapleuron dorsally and mesopleuron on antero-dorsal corner black (Figure 41); fore wing second submarginal cell long and rectangular, veins 2RS and 3RS forming a right angle, and vein r less than half length of 3RSa; wings hyaline; frons with lateral ridges (Figure 43)	*Aleiodes elleni* sp. n.
–	Hind coxa, propodeum, metanotum, metapleuron, and mesopleuron off-white (Figure 82); second submarginal cell shorter and trapezoidal (Figure 83), angle between veins 2RS and 3RS obtuse, vein r 0.85× vein 3RSa; wings moderately infuscate; lateral ridges on frons absent	*Aleiodes nebulosus* Townsend, 2009
12(3)	Ocelli small, ocell–ocular distance longer than width of lateral ocellus (Figures 14, 26, 89, 118)	13
–	Ocelli moderate-sized to large, ocell–ocular distance equal to or shorter than width of lateral ocellus (Figures 32, 46, 59)	23
13(12)	First and second metasomal terga with median carina present (Figures 23, 87); ovipositor sheaths at most 2/3 length of hind basitarsus	14
–	First and second metasomal terga with median carina absent (Figure 10); ovipositor about 2× length of hind basitarsus	*Aleiodes albiterminus* Townsend, 2009
14(13)	Malar space about as long as width of mandible base (Figure 11); head mostly black to dark brown, except for a crescent moon-shaped brown mark vertex, contrasting to thorax mostly yellow (Figure 12)	*Aleiodes arbitrium* Townsend, 2009
–	Malar space at least 1.25× width of mandible base (Figure 13); head and thorax coloration not as above: head and thorax mostly with same color, or head contrasting lighter than thorax (Figures 14, 22, 26)	15
15(14)	Occipital carina weak or interrupted at vertex (Figure 15)	16
–	Occipital carina complete and well-defined at vertex (Figures 26, 85)	20
16(15)	Mesopleuron with central disc lacking setae, smooth and shining (Figures 74, 77)	17
–	Mesopleuron with central disc setose and mostly granulate (Figures 16, 95)	19
17(16)	Tergite 2 mostly black with white markings, hind coxa black (Figures 74, 77, 78); hind wing vein M+CU distinctly shorter than 1M (Figure 76)	18
–	Tergite 2 entirely whitish, hind coxa yellowish (Figures 114, 116); hind wing vein M+CU about as long as 1M	*Aleiodes yanayacu* sp. n.
18(17)	Head, pronotum, propleuron and scutellum orangish yellow (Figure 74), except ocellar triangle black; tergite 1 entirely white; tergite 2 with median carina complete; mesoscutum with mid-posterior depressed area finely rugulose	*Aleiodes mirandae* sp. n.
–	Head and thorax black, except reddish brown mark on temples, just behind eyes; tergite 1 white with large black medial spot (Figure 77); tegite 2 with median carina incomplete, not reaching end of segment (Figure 78); mesoscutum not depressed postero-medially, entirely granular (Figure 79)	*Aleiodes napo* sp. n.
19(16)	Head orange, except for black ocellar triangle, contrasting with mostly black body (Figure 92); mesopleuron entirely black (Figure 95); metasoma stout, wider than propodeum, tergum 1 shorter than its apical width (Figure 94); ovipositor sheaths shorter than 1/2 length of hind basitarsus; hind wing vein m-cu distinct	*Aleiodes onyx* sp. n.
–	Head mostly yellowish brown with large black semicircular spot on occiput, vertex and ocellar triangle also black (Figures 14, 15); mesopleuron with ventral 1/2 yellowish brown, dorsally black (Figure 16); metasoma narrower than propodeum, tergum 1 about 1.2× longer than apical width (Figure 17); ovipositor sheaths longer than 1/2 length of hind basitarsus; hind wing vein m-cu absent (Figure 21) or at most weakly indicated by infumate pigmentation	*Aleiodes atripileatus* Townsend, 2009
20(15)	Central disc of mesopleuron smooth and shining (Figure 108); wings black; tarsal claws strongly pectinate (Figure 126); metasomal tergite 2 coarsely longitudinally costate (Figure 107)	*Aleiodes stilpnos* Townsend, 2009
–	Central disc of mesopleuron with various types of surface micro-sculpturing, not smooth and shining (Figure 28); wings clear or weakly infuscate; tarsal claws simple or weakly pectinate basally; metasomal tergite 2 with various weaker micro-sculpturing, not coarsely longitudinally costate (Figures 23, 27, 87, 88)	21
21(20)	Pronotum and mesonotum mostly black (Figure 28); hind coxa surface granulate	22
–	Pronotum and mesonotum yellowish brown (Figure 22); hind coxa rugose dorsally	*Aleiodes bimaculatus* sp. n.
22(21)	Mesoscutum with square orangish brown mark postero-medially (Figure 89); head mostly dark brown to black with crescent moon-shaped honey brown area bordering eyes at temples (Figure 84); metasoma mostly dark brown, light markings when present most frequent apically beyond tergite 4 (Figure 87), rarely reaching apex of tergite 1 in males (Figure 88)	*Aleiodes nubicola* sp. n.
–	Mesoscutum entirely black (Figure 26); head color variable, mostly yellowish with black occiput and vertex, and dark brown frons and face medially (Figures 25, 26); metasoma black with small basal spot on tergite 1 and finger-shaped mid-basal spot on tergite 2 (Figure 27)	*Aleiodes cacuangoi* sp. n.
23(12)	Mesosoma and metasoma mostly honey brown–honey yellow (Figures 45, 58, 104), reddish brown (Figure 31), sometimes with dark marks on mesoscutum (Figure 105)	24
–	Propodeum and most of metasomal terga black to dark brown (Figures 7, 98, 111), or sometimes first tergite white (Figure 52, 109); hind coxa sometimes bicolored black and white (Figures 7, 98, 109)	27
24(23)	Hind wing vein 2-1A present (Figure 119); diameter of lateral ocellus at least 2× ocell–ocular distance (Figure 59)	25
–	Hind wing vein 2-1A absent; diameter of lateral ocellus roughly as long as ocell–ocular distance (Figure 46)	*Aleiodes falloni* sp. n.
25(24)	Hind wing marginal cell gradually widening toward apex, vein RS well pigmented; dorsal carina on first tergite meeting subbasally, enclosing a basal polished triangular area (Figures 36, 106)	26
–	Hind wing marginal cell narrowest point at middle, vein RS faint, difficult to trace (Figure 119); dorsal carina on first tergite meeting basally enclosing a semicircular basal area (Figure 37)	*Aleiodes luteosicarius* sp. n.
26(25)	Antenna dark brown to brown proximally, lightening gradually toward pale brown apex (Figure 104); wings uniformly weakly infuscate; ocelli large, about 2× ocell–ocular distance (Figure 105)	*Aleiodes stewarti* sp. n.
–	Antenna black with white median annulus (Figure 31); wings weakly infuscate with dark band just bellow stigma (Figure 31); ocelli extremely large, about 8× ocell–ocular distance (Figure 32)	*Aleiodes colberti* sp. n.
27(23)	Malar space moderately wide, at least slightly longer than width of mandibular base; median carina present on propodeum	28
–	Malar space short, length 0.7× width of mandibular base (Figure 1); median carina absent on propodeum	*Aleiodes aclydis* Townsend, 2009
28(27)	Mesoscutum and scutellum honey yellow, hind coxa bicolored black and white (Figures 7, 98, 109)	29
–	Mesoscutum and scutellum mostly black (Figures 52, 111); hind coxa one color, either black (Figure 52), yellow, or white (Figure 111)	31
29(28)	Head honey yellow, ocellar triangle dark brown (Figure 99); fore wing vein 1M strongly curved basally (Figure 120)	30
–	Head dark brown, gena white (Figure 7); fore wing vein 1M almost straight or weakly and evenly curved (as in Figure 119)	*Aleiodes albigena* sp. n.
30(29)	First metasomal tergite about 2× longer than its apical width, dark brown to black (Figure 100); hind coxa basally white and apically black (Figures 98, 100)	*Aleiodes shakirae* sp. n.
–	First metasomal tergite about as long as apical width, white with small black spot mid-apically; hind coxa black basally and apically white (Figure 109)	*Aleiodes townsendi* sp. n.
31(28)	First metasomal tergite white, contrasting to remainder mostly dark brown metasoma (Figure 103); pronotum mostly black; mesopleuron dark brown at least on anterior corner (Figure 52)	32
–	First metasomal tergite dark brown; pronotum white with brown mark dorsally; mesopleuron entirely yellow (Figure 111)	*Aleiodes tzantza* sp. n.
32(31)	Mesopleuron, metapleuron, hind coxa and propodeum medially and anteriorly smooth; mesopleuron except for anterior corner and hind coxa orangish	*Aleiodes speciosus* Townsend, 2009
–	Mesopleuron, metapleuron, hind coxa and propodeum granulate; mesopleuron and hind coxa black (Figure 52)	*Aleiodes kingmani* sp. n.

## Systematics

### Aleiodes aclydis

Townsend, 2009

http://species-id.net/wiki/Aleiodes_aclydis

Figure 1

#### Diagnosis.

Body length 6.1 mm; antenna with 44 segments; head with vertex black, occiput light orangish brown; ocelli large, ocell–ocular distance less than width of lateral ocellus; occipital carina interrupted at vertex; mesosoma mostly light orangish brown, except propodeum black; wings slightly darkened; mesopleuron granulate; apex of hind tibia without comb of modified setae; propodeum without median propodeal carina; metasomal terga entirely black; metasomal tergum 3 costate on anterior 2/3, with median carina along with this sculpturing; ovipositor short, about 0.25× length of hind basitarsus.

#### Additional characters.

Last flagellomere with “bottle nipple”-like tip; mesoscutum with carina only in front of scutellar sulcus; scutellar sulcus with complete median carina plus two pairs of weak and incomplete lateral carina; fore wing vein 1M only slightly curved at base; hind wing vein 2-1A absent, vein m-cu present and well pigmented, antefurcal to r-m in left wing and interstitial in the right wing; ovipositor sheaths about as long as hind tarsomere II, 0.6× hind basitarsus.

Type material examined. (UWIM)

#### Biology.

*Aleiodes aclydis* has been reared from an unidentified Geometridae found on *Ocotea* sp. (Lauraceae).

#### Distribution.

Known only from the type locality, Isla de Las Palmas, Napo province, ECUADOR, at 1,883 meters elevation.

#### Discussion.

From the newly described species, *Aleiodes aclydis* is very similar to *Aleiodes albigena* sp. n. in color features, including a mostly blackish head with a lighter gena. However, in *Aleiodes aclydis* the gena is yellow instead of the white gena of *Aleiodes albigena* sp. n. Both species belong to *circumscriptus*/*gastritor* species-group but *Aleiodes aclydis* is the unique species with a malar space as short as 0.7 times the basal width of mandibles. No additional *Aleiodes aclydis* specimens were found since Townsend and Shaw’s (2009) work. The dorso-medially elevated area on mesopleuron is well-demarcated posteriolly. In the original description the sulcus demarcating this region is called the “sternaulus” (Townsend and Shaw 2009). We now consider that this sulcus is not a true sternaulus, as defined by Sharkey and Wharton 1997, and neither is it the precoxal sulcus, as defined by other authors (van Achterberg 1991, Wharton 2006). Additionaly, the absence of sternaulus is a common feature in all species treated in this work. Therefore, this term is avoided in the descriptions of the new species. The same matter is found in the descriptions of *Aleiodes atripileatus* Townsend and *Aleiodes nebulosus* Townsend.

**Figures 1–9.**
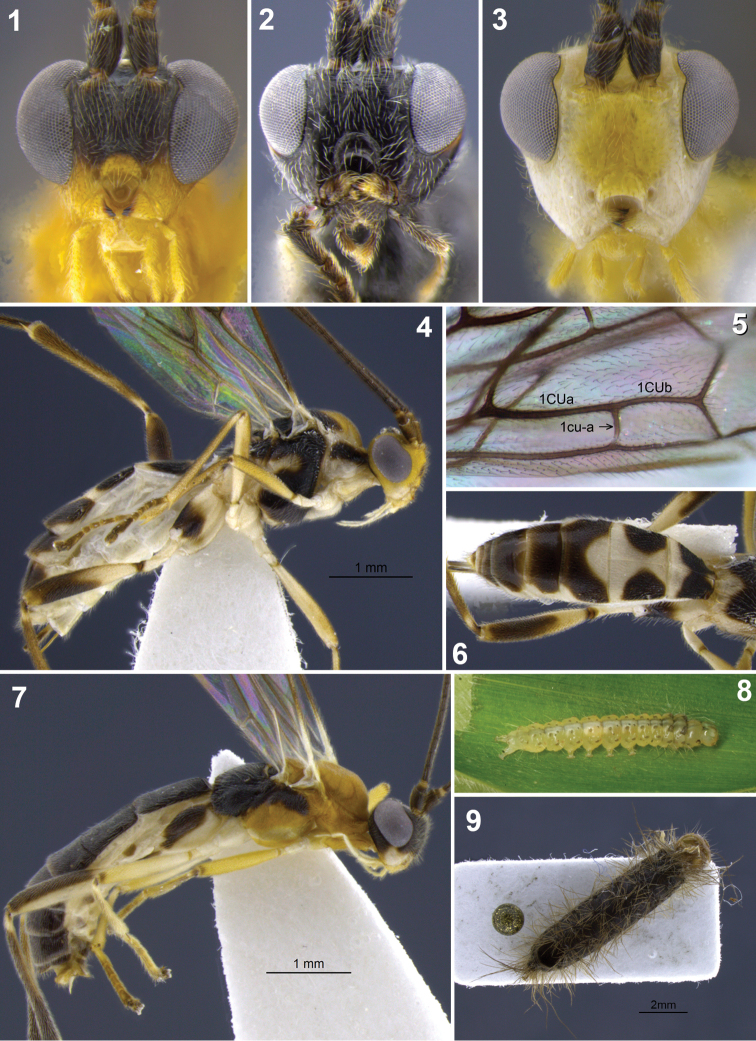
**1–3** head, frontal. **1** *Aleiodes aclydis* Townsend **2** *Aleiodes capillosus* Townsend; *Aleiodes marilynae* sp. n. **4–6** *Aleiodes albidactyl* sp. n. **4** habitus **5** fore wing, detail of 1^st^ subdiscal and subbasal cells **6** metasoma, dorsal **7–9** *Aleiodes albigena* sp. n. **7** habitus **8** host larva, nr. *Desmotricha* (Erebidae) **9** host mummy after parasitoid emergence.

### Aleiodes albidactyl sp. n.

http://zoobank.org/4969BD1E-3D57-4EC5-B5A0-F6B097D0148C

http://species-id.net/wiki/Aleiodes_albidactyl

Figures 4–6

#### Description of holotype.

Female (holotype). Body length 5.2 mm; antenna length 6.6 mm; fore wing length 4.9 mm.

Color. Distinctive black and white color pattern. Head mostly pale yellow with small dark brown spot just above clypeus; occiput laterally and stemmaticum dark brown. Antenna dark brown, scape and pedicel brown. Mesosoma mostly black; mesoscutum mostly pale yellow with postero-lateral borders dark brown; metapleuron, propodeum posteriorly based triangular area, thumb shaped area posteriorly on mesopleuron, ventral half of pronotum, and propleuron white. Metasoma mostly black dorsally and completely white ventrally; T1 apical 1/3 and small mid-basal spot white; T2 mostly white with two round antero-lateral spots; T3 with white semicircular basal spot; lateral borders of remainder terga white; ovipositor sheaths basal 1/5 white, remainder dark brown. Fore and mid legs pale light brown. Hind legs brown; basal half of femur and coxa, trochanter and trochantellus white. Wings very weakly infuscate, veins dark brown.

Head. Antenna 50 antennomeres, apical flagellomere with “bottle-nipple”-shaped apex; malar space slightly longer than basal width of mandible, 0.4× eye height; in dorsal view eyes 2.75× longer than temples; occipital carina incomplete, directed toward vertex, getting close to lateral ocelli, well defined laterally and meeting hypostomal carina; oral space small and circular, maximum width slightly smaller than basal width of mandible; clypeus not swollen; ocell–ocular distance as long as diameter of lateral ocellus; maxillary palp not swollen; head surface sculpture shining granulate, occiput smooth and shining; vertex with few wrinkles; higher face with few transverse rugosity; frons smooth with pairs of wrinkles concentric to each toruli, frons excavated with excavation bordered by a “W-shaped” carina.

Mesosoma. Sculpturing granulate; pronotum smooth latero-ventrally, latero-dorsally foveate; mesopleuron with antero-dorsal corner rugose, mid-longitudinally depressed area foveate; propodeum coarsely shining granular with complete mid-longitudinal carina; notauli very shallow anteriorly, virtually absent; posterior margin of mesoscutum with complete carina; scutellar sulcus with five carina, relatively strong but irregular and not reaching anterior margin of sulcus.

Wings. Fore wing: stigma 3.4× longer than high; vein r about as long as vein 2RS, 1.5× longer than vein RS+Mb, and 0.8× vein m-cu; vein 3RSa 0.43× vein 3RSb, and 0.9× vein 2M; vein 1CUa 3.3× vein 1cu-a; vein 1CUb 0.7× vein 1CUa; vein 1M slightly and evenly curved. Hind wing: vein m-cu only very weakly indicated; vein M+CU 1.8× longer than vein 1M; 1M slightly shorter than r-m; RS smoothly curved at middle; vein 1M straight, almost unpigmented; vein 2-1A absent.

Legs. Hind tibia with comb of modified setae; tarsal claws simple, with a comb of relatively long thin setae basally; hind basitarsus 3.6× longer than inner apical spur on hind tibia.

Metasoma. T1, T2 and basal 2/5 of T3 granulose-striate, longitudinal carina present along this sculpturing; remainder visible terga granular coriaceous; ovipositor sheaths about as long as hind tarsomere II; ovipositor sheaths parallel sided with lanceolate apex; T1 about as long as its apical width.

Male unknown.

#### Type material.

Type-locality: ECUADOR, Napo Province, Yanayacu Biological Station, Macucoloma trail, S00°35.9', W77°53.4', 2163 m, cloud forest, August 17, 2006, A. Townsend.

Type-specimen: Holotype female, point mounted. Top label: “ECUADOR: Napo Prov. / Yanayacu Biological Station / 17-Aug-2006 Macucaloma trail / Yellow pan 2163m. A. Townsend” (UWIM).

#### Discussion.

This species belongs to *seriatus* species-group. It differs from all other species of this group by the white posterior thumb like marking on the black mesopleuron, and the long vein 1CUa on fore wing, about 1.5× longer than 1CUb and 3.5× vein 1cu-a. This species is most closely related to *Aleiodes dyeri* sp. n., but the color patterns of both species are quite distinct (see comments for *Aleiodes dyeri* sp. n.).

#### Etymology.

From the Latin roots meaning “white finger”, a reference to the white thumb like mark on mesopleuron.

### Aleiodes albigena sp. n.

http://zoobank.org/E47103FE-3B7C-4AF0-8646-C67E62D7E968

http://species-id.net/wiki/Aleiodes_albigena

Figures 7–9

#### Description of holotype.

Male (holotype). Body length 5.2 mm; antenna length 7.0 mm; fore wing length 5.0 mm.

Color. Mostly black. Head and antenna dark brown; gena, mandibles and palp white, mandible tips dark brown. Pronotum, propleuron, mesoscutum, most of mesopleuron and scutellum honey yellow; metanotum, propodeum, metapleuron and dorsal 1/4 of mesopleuron, including the border with metapleuron, black; mesopleuron with whitish longitudinal stripe. Legs: all coxa, trochanter and trochantellus, mid femur and tibia, and most hind femur white; fore femur yellowish; fore tibia and tarsi and mid tarsi light brown; hind tibia and tarsi dark brow to black, but tibia with white basal band and fourth and fifth tarsi lighter; hind coxa, trochanter, trochantellus and femur black dorsally, except basal 1/5 of femur; tip of fore and mid femur and mid tibia with infuscate stains. Metasoma dark brown to black dorsally, laterally and ventrally white, but some lateral spots and the last sternites dark brown.

Head. 46 antennomeres, flagellomeres roughly 2.0× as long as wide, apical flagellomere with short pointed apex; malar space moderate, length 1.4× basal width of mandible, and 0.4× eye height; in dorsal view eye height about 3× temples; occipital carina complete, ventrally touching hypostomal carina; oral space small and circular, maximum width slightly smaller than basal width of mandible; clypeus not swollen; ocell–ocular distance 0.6× diameter of lateral ocellus; maxillary palp not swollen. Face somewhat bulging, frons barely excavated and without lateral ridges. Head surface sculpturing, including frons, granulate, face coarsely granulate, occiput smooth and shining.

Mesosoma. Sculpturing mostly granulate; pronotum with few laterally running wrinkles laterally; mesopleuron rugose on dorso-anterior corner; propodeum rugose–granulate, with complete mid-longitudinal carina; notauli very shallowly indicated anteriorly, posteriorly disappearing in a depressed rugose area; posterior margin of mesoscutum with very short carina, just anterior to scutellar sulcus; scutellar sulcus with five short carina, not reaching anterior margin of sulcus.

Wings. Fore wing: stigma 5× longer than high; vein r 0.5× vein 2RS, about 0.64× vein RS+Mb, and 0.56× as long as vein m-cu; vein 3RSa about 0.4 times vein 3RSb, and as long as vein 2M; vein 1CUa 2.7× vein 1cu-a; vein 1CUb about 2× vein 1CUa; vein 1M evenly slightly curved. Hind wing: m-cu indicated as short nebulose vein interstitial to vein r-m; vein M+CU as long as vein 1M; vein 1M 1.5× vein r-m; RS smoothly curved at middle; vein 2-1A present as a short stub.

Legs. Hind tibia without comb of modified setae; tarsal claw simple, with a comb of thin bristles medially; hind basitarsus 2.5× length of inner apical spur of hind tibia.

Metasoma. T1–T3 granular-striated; remainder terga granular; mid longitudinal carina complete from T1 throughout T2; T1 length 1.3× its apical width.

Female. Unknown.

Mummy. Length 9.0 mm, body reddish brown, head yellowish, setae pale brown, covered with setae, thorax compact and wrinkled, glue hole located ventrally on the thorax, exit hole irregular, located postero-dorsally, posterior to hind abdominal prolegs.

#### Type material.

Type-locality: ECUADOR, Napo Province, Yanayacu Biological Station, S00°35.9', W77°53.4', 2163 m, cloud forest, May 14, 2010.

Type-specimen: Holotype female and mummy, point mounted. Top label: “ECUADOR: Napo Province / Yanayacu Biological Station / S00°35.9', W77°53.4’ 2163m / CAPEA – NSF-BSI-07-17458 / (hand written) April 2010 / 47082”, back (hand written): “14-MAY-2010”; bottom label (hand written): “coll. 8 April 2010 / YY road, Chusquea / Arctiidae, inst. 2 / Beat 650 / pup. 23 April / em. 14 May 2010” (UWIM).

#### Biology.

Reared from a species near *Desmotricha* Hampson (Erebidae) larvae (voucher number YY-47082), feeding on *Chusquea scandens* (Poaceae). Parasitoid took three weeks to emerge after host mummification. This is the only described *circumscriptus*/*gastritor*-group species in Neotropical region known to attack Arctiinae hosts, producing a densely setose mummy.

#### Discussion.

*Aleiodes albigena* sp. n. belongs to *circumscriptus*/*gastritor* species-group. Some diagnostic characters are the very shallow notauli present only anteriorly; scutellar sulcus without bisecting carina; occipital carina complete dorsally; face bulging; and frons surface granular, not excavated, without a lateral carina. This species is similar to *Aleiodes townsendi* sp. n. and *Aleiodes shakirae* sp. n., but it can be distinguished from both by the mostly black head with contrasting whitish gena, yellowish in *Aleiodes townsendi* sp. n. and *Aleiodes shakirae* sp. n., and the almost straight vein 1M on fore wing, strongly curved in *Aleiodes townsendi* sp. n. and *Aleiodes shakirae* sp. n. *Aleiodes albigena* sp. n. resembles *Aleiodes arbitrium* in color pattern but differs in the shorter ocell–ocular distance relative to lateral ocelli diameter.

#### Etymology.

From the Latin roots meaning “white cheeks,” named in reference to the contrasting white gena, as compared with the black head of this species.

### Aleiodes albiterminus

Townsend, 2009

http://species-id.net/wiki/Aleiodes_albiterminus

Figure 10

#### Diagnosis.

Body length 4.4 mm; antenna with 33 segments; head color mostly black, with light orangish brown mark below eye; malar space 1.25× basal width of mandible, ocelli small, ocell–ocular distance 1.3× width of lateral ocellus; occipital carina interrupted at vertex; mesopleuron granulate; apex of hind tibia without comb of flattened setae; propodeum mostly granulate, with median propodeal carina present but incomplete; metasomal tergum 3 black basally, apically with irregular “half heart-shaped” off-white spot; ovipositor sheaths long, 1.3× length of hind basitarsus.

#### Additional characters.

Last flagellomere lanceolate; mesoscutum with complete carina on posterior margin though not well defined; scutellar sulcus with a strong complete median carina and some irregular carina laterally; fore wing vein 1M slightly and evenly curved; hind wing vein 2-1A present as a very short stub, vein m-cu present and distinctly postfurcal to vein r-m; ovipositor sheaths 1.3× longer than hind basitarsus.

Type material examined. (UWIM)

#### Biology.

*Aleiodes albiterminus* has been reared from an unidentified Geometridae larva on *Alnus acuminata* (Betulaceae).

#### Distribution.

Known only from the type locality, Rio Chalpi Grande, Napo province, ECUADOR, at 2,837 meters elevation.

#### Discussion.

For standardization reasons we provide the measurement of the ovipositor sheaths instead of the ovipositor itself. The ovipositor and the sheaths length in *Aleiodes albiterminus* are unusually long for *Aleiodes* species. This is the only species from Ecuadorian Northeastern Andes with the ovipositor sheaths longer than its hind basitarsus. *Aleiodes albiterminus* can be distinguished also by the absence of a median carina on the first to third metasomal terga, and the distinctive off-white markings at the apex of metasomal tergum 3.

**Figure 10–17.**
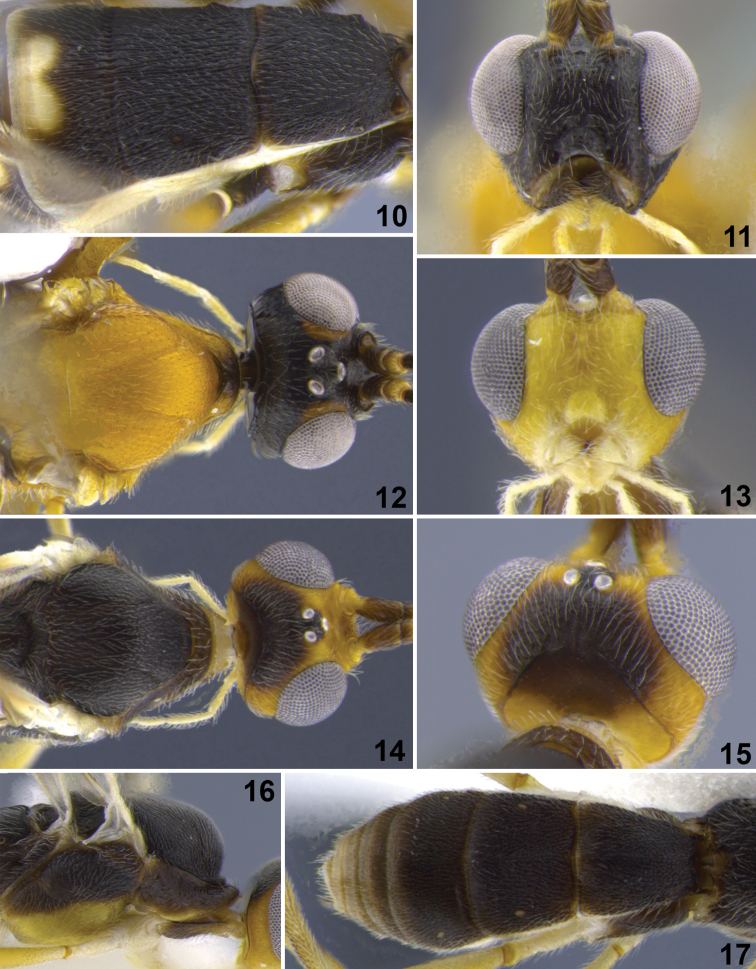
**10** *Aleiodes albiterminus* Townsend, metasoma, dorsal. **11, 12** *Aleiodes arbitrium* Townsend **11** head frontal **12** head and mesoscutum, dorsal **13–17** *Aleiodes atripileatus* Townsend **13** head, frontal **14** head and mesoscutum, dorsal **15** head, occiput and vertex **16** thorax, lateral **17** metasoma, dorsal.

### Aleiodes albiviria sp. n.

http://zoobank.org/099A4E94-7013-4B08-930B-1DB134C7EB3E

http://species-id.net/wiki/Aleiodes_albiviria

Figures 18 –21

#### Description of holotype.

Female (holotype). Body length 7.1 mm; antenna length 9.0 mm; fore wing length 5.8 mm.

Color. Mostly yellowish “bronze” (orangish yellow). Ocellar triangle brown. Antenna black; scape yellowish ventrally; flagellum with white band medially from flagellomeres 18–19^th^ to 29–30^th^. Brown regions of mesosoma: mesoscutum lateral borders, notauli region and depressed posterior area; lunules; metanotum; propodeum. Dorsal mesopleuron and metasoma ventrally pale yellow. Wings tinged honey yellow, veins honey brown.

Head. Antenna with 57 antennomeres, mid flagellomere roughly 2.0× longer than wide, apical flagellomere with small pointed apex; malar space moderate, about 1.25× longer than basal width of mandible, 0.33× eye height; in dorsal view eyes 3× longer than temples; occipital carina incomplete, directed toward vertex, well defined laterally and meeting hypostomal carina; oral space small and circular, maximum width equal to basal width of mandible; clypeus not swollen; ocelli moderate, ocell–ocular distance slightly shorter than diameter of lateral ocellus; maxillary palp not swollen; head surface sculpture finely shining granulate, occiput smooth and shining; higher face with a small longitudinal ridge and transverse rugosity directed to it; frons excavated, excavation bordered by a weak “W-shaped” carina.

Mesosoma. Sculpturing shining granulate; pronotum foveate laterally; mesopleuron with antero-dorsal corner rugose; propodeum granular with some longitudinal diverging wrinkles laterally, mid-longitudinal carina present only on anterior 1/3; notauli with few crenulae and shallow anteriorly, meeting on depressed rugose area posteriorly; posterior margin of mesoscutum with complete carina; scutellar sulcus with median carina plus two pairs of complete lateral carina and one irregular.

Wings. Fore wing: stigma 3.8× longer than high; vein r as long as vein 2RS, 1.2× longer than vein RS+Mb, and 0.78× vein m-cu; vein 3RSa 0.45× vein 3RSb, and 0.9× vein 2M; vein 1CUa 2.7× vein 1cu-a; vein 1CUb about as long as vein 1CUa; vein 1M weakly curved basally. Hind wing: vein m-cu present, antefurcal to r-m; vein M+CU about 1.6× 1M; vein 1M almost as long as r-m; vein RS gradually opening from wing margin; vein 1M straight, dark brown, well pigmented; vein 2-1A absent.

Legs. Hind tibia with comb of modified setae; tarsal claw simple, with a comb of relatively long thin setae basally; hind basitarsus 3.5× longer than inner apical spur of hind tibia.

Metasoma. T1, T2 and basal 2/5 of T3 costate, longitudinal carina present along this sculpturing; remainder visible terga smooth; ovipositor sheaths short and lanceolate, about as long as hind tarsomere IV (half length of tarsomere II); T1 1.4× longer than its apical width.

Male unknown.

#### Type material.

Type-locality: ECUADOR, Napo Province, Yanayacu Biological Station, Macucoloma trail, S00°35.9', W77°53.4', 2163 m, cloud forest, April 1–8, 2007, J. Simbaña col.

Type-specimen: Holotype female, point mounted. Top label: “ECUADOR: Napo Province / Yanayacu Biological Station / S00°35.9', W77°53.4’ 2163m / 1-8 April 2007, J. Simbaña /Macucoloma trail, Malaise trap / CAPEA - NSF-BSI-07-17458, S.R. Shaw”; bottom label: “SRS - 00043” (UWIM).

#### Discussion.

This species is assigned to the *seriatus* species-group, where it most resembles *Aleiodes greeneyi* Townsend because of the dorsally incomplete occipital carina. *Aleiodes albiviria* sp. n. differs from other New World species of this species group by the mostly honey brown body with dark brown notauli and mid-posterior mesoscutum, and the white middle band on blackish antenna. *Aleiodes albiviria* sp. n. also resembles the Brazilian *Aleiodes scriptus* (Enderlein, 1920), by the costate sculpturing on metasomal tergite 1, which is rugose–costate in all other Neotropical species in *seriatus* species-group, but differs from *Aleiodes scriptus* by the shape of hind wing vein RS (parallel to wing margin basally and bent downward apically, as opposed to sinuate at middle in *Aleiodes scriptus*).

#### Etymology.

From the Latin *albus*, meaning “white,” and *viria* meaning “bracelet,” a reference to the white band on the antenna.

**Figures 18–20.**
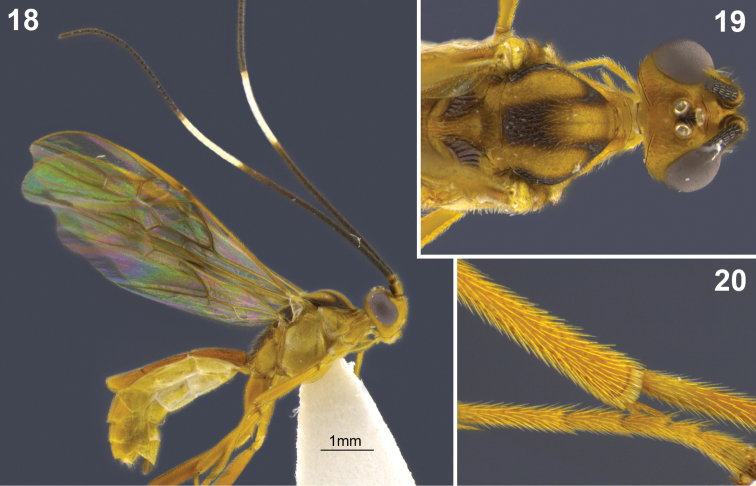
*Aleiodes albiviria* sp. n. **18** habitus **19** head and mesonotum, dorsal **20** hind tibia inner side with comb of modified setae at apex.

### Aleiodes arbitrium

Townsend 2009

http://species-id.net/wiki/Aleiodes_arbitrium

Figures 11 , 12

#### Diagnosis.

Body length 5.7 mm; antenna with 43 segments; head mostly black; malar space slightly wider than basal width of mandible; ocelli small, ocell–ocular distance slightly wider than width of lateral ocellus; occipital carina at least shortly interrupted at vertex; mesosoma mostly light yellowish brown, except propodeum black; wings clear; mesopleuron granulate; apex of hind tibia without comb of flattened setae; propodeum rugulose basally, granulate apically, with median propodeal carina present; metasomal terga mostly black, except terga 2 and 3 often each with an oval-shaped off white marking; metasomal terga 1–3 costate, median carina complete to end of tergum 2; ovipositor sheaths slightly shorter than half of hind basitarsus.

#### Additional characters.

Last flagellomere with short pointed tip; mesoscutum with carina on posterior margin only in front of scutellar sulcus; scutellar sulcus with a median carina barely complete plus two or three more or less weak and incomplete carina laterally; fore wing vein 1M slightly and evenly curved; hind wing vein 2-1A present as a very short stub, vein m-cu position varying from distinctly antefurcal to interstitial to vein r-m; ovipositor sheaths 0.45 to 0.49 times longer than hind basitarsus.

#### Morphological variation.

All specimens examined agree well with original description of *Aleiodes arbitrium*, however the occipital carina in all specimens is at least shortly interrupted at vertex, and not complete but weak at vertex as described originally. The two spots on terga vary in size. In some females it is darker, not so contrasting, while in others it is larger, with the T2 spot reaching apical margin of tergite. In some males the spots tends to meet and form one single irregular elongate spot. In all specimens the gena is black–dark brown but the borders with mandibles are light brown.

#### Material.

Type material examined. (UWIM)

Non-type material: 14 females and 9 males, ECUADOR, Napo, Yanayacu Biological Station, Napo Province, S00°35.9', W77°53.4', 2,163 m. 7 females and 6 males reared from Geometridae: ♂, YY-29185; ♀, YY-36318; ♂, YY-39980; ♂, YY-39990; ♀, YY-47187; ♀, YY-47708; ♀, YY-48161; ♂, YY-49147; ♂, YY-53729; ♂, YY-57356; ♀, YY-62371; ♀, YY-70773; ♀, YY-71345. 5 females collected by Malaise trap; 2 females and 3 males collected by yellow pan trap. (UWIM)

#### Biological additions.

All new *Aleiodes arbitrium* Townsend rearings were from caterpillars feeding on polypod ferns: *Diplazium costale* (Dryopteridadceae) or *Dennstaedtia cornuta* (Dennstaedtiaceae). Most caterpillars were identified as Geometridae. Caterpillar pictures and mummy morphology corroborate family level identification, and two different species of *Psaliodes* Guenée (Geometridae), and one species of Pyralidae as hosts of *Aleiodes arbitrium*.

The holotype and eight non-type specimens were reared from *Psaliodes* sp. larva feeding on *Diplazium costale* or *Dennstaedtia cornuta*. The two paratypes and four other non-type specimens were reared from *Psaliodes castanea* (Warren) feeding on *Diplazium costale*. A single non-type specimen was reared from unknown Pyralidae larva on *Dennstaedtia cornuta*. One paratype collected from Urticaceae did not feed, so may have simply wandered there prior to mummification.

#### Distribution.

Known only from the Yanayacu Biological Station, Napo province, ECUADOR, at 2,163 meters elevation.

#### Discussion.

After publication of Townsend and Shaw (2009) paper, 11 new specimens were reared and ten collected by traps, all from Yanayacu Biological Station, Napo Province, S00°35.9', W77°53.4', 2163. This additional reared material provided good biological information at least on feeding preferences of the host caterpillars. It is likely that *Aleiodes arbitrium* attacks mostly Geometridae species feeding on polypod ferns. In the *circumscriptus*/*gastritor* group, this species resembles *Aleiodes albigena* sp. n. in most color patterns; however, the smaller ocelli of *Aleiodes arbitrium* and the honey brown marks on vertex, absent in *Aleiodes albigena*, distinguish these two species. Other species with small ocelli and interrupted occipital carina, as *Aleiodes onyx* sp. n. and *Aleiodes atripileatus*, have more extensively dark bodies than *Aleiodes arbitrium*.

### Aleiodes atripileatus

Townsend, 2009

http://species-id.net/wiki/Aleiodes_atripileatus

Figures 13–17 , 21

#### Diagnosis.

Body length 4.1–4.8 mm; antenna with 36–39 segments; head color black dorsally, light orangish brown ventrally; malar space 1.5× basal width of mandible, ocelli small, ocell–ocular distance 1.7× width of lateral ocellus; occipital carina interrupted at vertex; mesopleuron granulate; apex of hind tibia without comb of flattened setae; propodeum mostly rugulose-granulate, with complete median carina; metasomal terga 1–4 entirely dark brown to black; ovipositor sheaths about 2/3 of the hind basitarsus length.

#### Additional characters.

Last flagellomere with short “bottle-nipple”-like tip; mesoscutum posterior margin with carina only in front of scutellar sulcus; scutellar sulcus with a median plus two or three pairs lateral carina well defined and almost complete; fore wing vein 1M only slightly and evenly curved; hind wing vein 2-1A absent, vein m-cu absent; ovipositor sheaths about 2/3 length of hind basitarsus, one specimen in type series have 2.5/3, and another 1.8/3 proportions for this character.

#### Material.

Type material examined. (UWIM)

Non-type material: 3 females and 2 undetermined sex (?), ECUADOR, Napo, Yanayacu Biological Station, Napo Province, S00°35.9', W77°53.4', 2163 m; voucher numbers: ♀, YY-28538: prob. Noctuidae (*Hypena* sp2) on *Phenax rugosus* (Urticaceae); ♀, YY-29079: *Hypena* sp2 (Noctuidae) on *Boehmeria* ulmifolia (Urticaceae); ♀, YY-37062: Noctuidae on *Phenax rugosus* (Urticaceae); ?, YY-58626: Noctuidae on *Phenax rugosus* (Urticaceae); ?, YY-63838: Noctuidae on *Phenax rugosus* (Urticaceae). (UWIM)

#### Biology.

*Aleiodes atripileatus* has been reared from a species of *Hypena* Schrank (Noctuidae) caterpillars feeding on Urticaceae, including *Phenax rugosus*, *Boemeria bullata*, *Miriocarpa* sp., and three other unidentified urticaceous plants. Other noctuids on the same host plants might be utilized as hosts.

#### Distribution.

Known only from the YBS, Napo province, ECUADOR, at 2,163 meters elevation.

#### Discussion.

*Aleiodes atripileatus* is one of the most commonly reared *Aleiodes* species in YBS. The species belongs to the *circumscriptus*/*gastritor* species-group. It is very similar to *Aleiodes nubicola* sp. n. and *Aleiodes cacuangoi* sp. n., differing from these two species in having the occipital carina distinctly interrupted at vertex. This character is shared with *Aleiodes onyx* sp. n., from which it differs by having a black vertex, a thinner metasoma, and the vein m-cu of hind wing absent. Different than originally described, the true sternaulus is absent.

**Figures 21.**
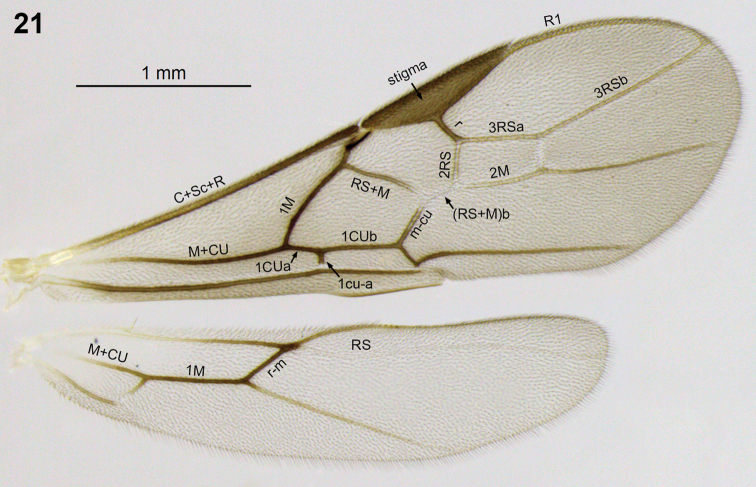
*Aleiodes atripileatus* Townsend wings.

### Aleiodes bimaculatus sp. n.

http://zoobank.org/65E1286E-44D3-48DF-A403-F895A9D504A1

http://species-id.net/wiki/Aleiodes_bimaculatus

Figures 22–24

#### Description of holotype.

Female (holotype). Body length 5.6 mm; antenna length 6.4 mm; fore wing 5.4 mm.

Color. Mostly honey brown. Antenna brown, scape and pedicel honey brown as head; cheeks and palp light yellow; ocellar triangle brown; fore and mid coxa, and all trochanter and trochantellus whitish; metanotum and propodeum dark brown; metasoma dark brown dorsally with mid-apical pale yellow spots on T1 and T2; ovipositor sheaths brown. Wings hyaline with brown veins and stigma, parastigma contrasting darker–black.

Head. Antenna 44 segmented, apical flagellomere lanceolate, without pointed tip; malar space about 1.3× longer than mandible basal width, and 0.6× eye height; occipital carina complete; oral space moderate and circular, maximum width about equal to basal width of mandible; clypeus swollen; ocelli small, ocell–ocular distance 1.4× diameter of lateral ocellus; in dorsal view temples almost as long as eye height; head sculpturing mostly granular, face coarsely granular to rugose, occiput smooth.

Mesosoma. Sculpturing mostly granulate; pronotum covered with wrinkles; mesopleuron mostly rugose otherwise granulate, wrinkles stronger on sternaulus area; metapleuron with rugosity posteriorly; propodeum rugose, with complete mid-longitudinal carina; notauli deep and mostly smooth anteriorly, with two or three crenulae, meeting on rugose area posteriorly; posterior margin of mesoscutum bordered by short carina just anterior to scutellar sulcus; scutellar sulcus with median carina plus one pair of lateral carina.

Wings. Fore wing: stigma 3.5× longer than high; vein r 0.65× vein 2RS, 1.2× vein RS+Mb, and 0.7× as long as vein m-cu; vein 3RSa about 0.5× vein 3RSb, and as long as vein 2M; vein 1CUa 1.5× vein 1cu-a; vein 1CUb 3.0× vein 1CUa; vein 1M evenly slightly curved. Hind wing: m-cu indicated as short not tubular vein interstitial to vein r-m; vein M+CU 1.4× longer than vein 1M; vein 1M 1.2× vein r-m; RS smoothly curved at middle; vein M unpigmented; vein 2-1A present as a short stub.

Legs. Hind tibia without comb of modified setae; tarsal claw simple, with a comb of relatively long thin setae basally; hind coxa rugose dorsally; hind basitarsus 3.5× longer than inner apical spur on hind tibia.

Metasoma. T1 and T2 rugose costate with granulate background; T3 longitudinally striate basally; remainder terga granulate coriaceous; mid-longitudinal carina complete from T1 throughout T2; ovipositor sheaths about as long as hind tarsomere II, T1 1.2× longer than its apical width.

Paratype variation. Body length 5.0–6.0 mm; antennomeres 39–46; occipital carina is only very shortly interrupted in some paratypes, but never curved toward vertex; vein 2-1A of hind wing varying from short to absent. The patratypes from outside the YBS are distinctly smaller (body length 5.0–5.3 mm) than the type specimens from the YBS (5.4–6.0 mm), with fewer segments on antenna (39–40 *vs.* 43–46). The metasoma in this specimens is lighter than the holotype and paratypes from YBS: the apical terga beyond T3 are mostly honey yellow, and the spots on T1 and T2 are frequently larger, forming one large spot covering apical T1 and all T2 medially. We consider the specimens from Baeza as a geographical variant within *Aleiodes bimaculatus* sp. n. Since all but one females were collected at once and shows virtually none variation, the variation could be just an artifact. Further samplings could both confirm this hypothesis with some intermediate forms or support an alternative hypothesis (e.g. speciation process). The metasoma in one of the females from Manabí is almost entirely honey brown, the light spots are not contrasting but still visible.

Male. Body length 4.7–5.0 mm. Antennomeres 48–42. Considerable color pattern variation in males: antenna dark brown, pedicel brown, scape honey brown, face brown, hind coxa whitish, all tibia and tarsi darker, ocellar triangle black, metanotum dark brown as propodeum, T2 pale yellow spot varying from smaller than in female to covering most of the tergite, T3 also with pale yellow spot. The metasoma is narrower, T1 up to 1.4× longer than its apical width; eyes in dorsal view 1.55× longer than temple; occipital carina weak dorsally, barely interrupted at vertex. The males from Baeza follow the same pattern of the females, with the apical metasomal terga honey brown instead of dark brown, however the body length in these males is not distinctly shorter than the specimens from YBS.

#### Type material.

Type-locality: ECUADOR, Napo Province, Yanayacu Biological Station, Macucoloma trail, S00°35.9', W77°53.4', 2163 m, cloud forest, January 1–8, 2008, J. Simbaña col.

Type-specimen: Holotype female, point mounted. Top label: “ECUADOR: Napo Province / Yanayacu Biological Station / S00°35.9', W77°53.4’ 2163m / 1-8 January 2007, J. Simbaña / Macucoloma trail, Malaise trap / NSF-BSI-07-17458, S.R. Shaw”; bottom label: “SRS - 00037”. (UWIM)

Paratypes. 11 females and 2 males (UWIM), same locality as holotype, different date and/or method: 1♀, September 5, 2005, malaise trap (Pumayacu ridge); 1♀, March 1–6, 2006, hand collected at light sheet, G. Gentry col; 2♀, same data as holotype; 1♀, no date/method; 2♀, June 1–8, 2007, malaise trap; 1♀, June 10 – July 10, 2010, canopy malaise on bamboo, S.R. Shaw col; 1♀, December 3–10, 2007, malaise trap; 1♀, October 3–10, 2007, malaise trap; 1♀, August 3–10, 2007, yellow pan; 2♂ May 14, 2011, black light, N. Zikani col.; 2♂ May 10–20, 2011, gate pan trap, M. Bryant col. ECUADOR, 12 females and 7 males (CNC): 9♀ and 1♂, Napo, Baeza, 2000m, February 1979, Mason; 1♀ and 3♂, Napo, 5km South Baeza, 1700m, February 9, 1983, Masner & Sharkey; 3♂, Napo, Baeza, 1900m, February 9, 1983; 2♀ Manabi, Montecristi, 400m, February 6, 1983, Masner & Sharkey.

#### Discussion.

*Aleiodes bimaculatus* sp. n. belongs to *circumscriptus*/*gastritor* species group. It resembles *Aleiodes nubicola* sp. n. and *Aleiodes cacuangoi* sp. n. because of the complete occipital carina and the small ocelli. *Aleiodes bimaculatus* sp. n. differs from these species by the honey brown body color, with propodeum and metasoma dark brown and two pale yellow spots on T1 and T2, while *Aleiodes nubicola* sp. n. and *Aleiodes cacuangoi* sp. n. are mostly black. The stigma is yellowish in *Aleiodes bimaculatus* sp. n., but brownish in *Aleiodes cacuangoi* sp. n. and light brown to whitish in *Aleiodes nubicola* sp. n., and the body larger, 5.0–6.0 mm length while *Aleiodes cacuangoi* sp. n. and *Aleiodes nubicola* sp. n. are shorter, with maximum body length of 4.6 mm.

#### Etymology.

From the Latin roots *bi* = two and *macula* = stain, refers to the two distinctive yellow spots on the dark brown metasomal terga of this species.

**Figures 22–24.**
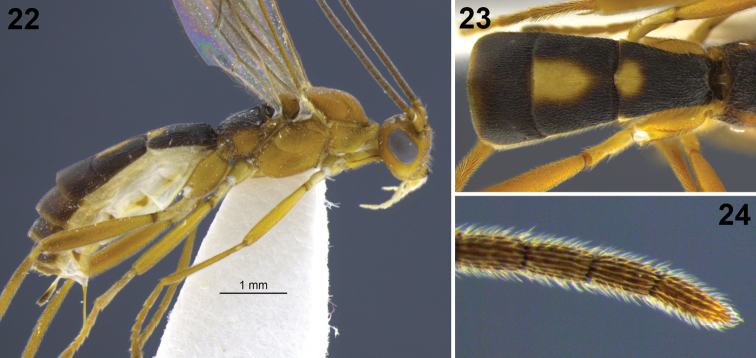
*Aleiodes bimaculatus* sp. n. **22** habitus **23** metasomal terga 1–3, dorsal **24** apical flagellomeres.

### Aleiodes cacuangoi sp. n.

http://zoobank.org/3A2C8D97-3E7C-4EF9-A86C-57C095F39946

http://species-id.net/wiki/Aleiodes_cacuangoi

Figures 25–30

#### Description of holotype.

Female (holotype). Body length: 4.77 mm; antenna length: 5.5 mm; fore wing length: 4.8 mm.

Color. Mostly black. Face, gena, eyes orbits, and apical half of hind coxa yellowish brown; mandibles, gena at border with mandibles and palp whitish, mandibles tips (tooth) brown; fore and mid legs yellowish darkening distally; tarsi dark brown, hind tibial spurs yellowish; ventral metasoma light yellow; metasomal terga 1 and 2 with mid basal whitish marks, spot on tergite 1 small, on tergite 2 extending over basal half. Ovipositor sheaths dark brown on apical half, basal half whitish.

Head. Antenna comprising 43 antennomeres, flagellomeres roughly 2.0× as long as wide, apical flagellomere with short pointed apex; malar space moderate, length 1.6× basal width of mandible, and approximately 0.55× eye height; occipital carina complete and well defined, bordered by a more or less deep sulcus on temples and vertex, reaching hypostomal carina; oral space small and circular, maximum width slightly smaller than basal width of mandible; clypeus not swollen; ocelli small, ocell–ocular distance about 1.5× diameter of lateral ocellus; temples about 1.8× eye height in dorsal view; maxillary palp not swollen; head surface sculpturing mostly granulate, vertex more coarsely granulate, occiput very weakly shining coriaceous, apparently smooth.

Mesosoma. Sculpturing mostly granulate; pronotum rugose laterally, foveate dorsally; mesopleuron rugose anteriorly, with elevated area centrally smooth and shining, and some wrinkles centrally, otherwise coriaceous; propodeum rugose posteriorly, with long mid-longitudinal carina on anterior 2/3; notauli deep and ﻿renulated anteriorly, meeting a coarsely punctate area posteriorly; scutellar sulcus with well defined median carina and two pairs of lateral irregular carina.

Wings. Fore wing: stigma 5× longer than high; vein r as long as veins 2RS and RS+Mb, and 0.7× as long as vein m-cu; vein 3RSa about 0.4× vein 3RSb, about as long as 2M; vein 1CUa 1.7× vein 1cu-a; vein 1CUb about 2.9× 1CUa; vein 1M evenly curved. Hind wing: m-cu absent; vein M+CU 1.36× 1M; vein r-m 1.5× 1M; vein RS smoothly curved at middle; vein 2-1A absent.

Legs. Hind tibia without comb of modified setae; tarsal claw simple, not pectinate, with a comb of relatively long thin setae basally; hind basitarsus about 3.4 times the length of inner apical spur on hind tibia.

Metasoma. T1 and T2 granulate and longitudinally striated; remainder terga granulate; mid longitudinal carina complete from T1 throughout T3; petiole length about 1.1× its apical width; ovipositor sheaths about as long as hind tarsomere II.

Paratype variation. Body length 4.2–4.6 mm; antennomeres 42–45; malar space about 1.4 times basal width of mandible; malar space/eye height = 0.53–0.60; ocell–ocular distance/diameter lateral ocellus~ 1.4–1.5; temple/eye height = 1.75–2.00; fore wing veins: r/2RS ~ 0.75–1, r/m-cu ~ 0.6–0.7, r/RS+Mb = 0.75–1.00, 1CUb/1CUa = 2.4–2.9; hind wing veins: 1M/r-m ~ 1.40–1.55, M+CU/1M = 1.15–1.40, in most paratype m-cu weak but present, its position varying from just antefurcal to interstitial (frequently in the same individual); hind basitarsus/apical inner spur on hind tibia = 3.2–3.4; tergite 1 length/apical width = 1.1–1.2; ovipositor sheaths/hind tarsomere II ~ 0.8–1.1. Color pattern: some paratypes have brown face medially, and/or a white stripe on mesopleuron; occipital carina somewhat irregular at vertex in few specimens; one or two pairs of lateral carina on scutellar sulcus, more or less irregular.

Male. Similar to females but body slightly smaller, antenna shorter, with 40–41 segments, metasoma slightly thinner, and ocelli relatively larger: ocell–ocular distance 1.25× diameter of lateral ocellus. In one specimen the occipital carina is shortly interrupted but the occiput and vertex limits are easily distinguishable. One male paratype have considerably darker head and metasoma: head brown to dark brown, gena at borders with mandibles pale yellow, middle face darker, clypeus and most of temples honey brown, white spots on metasoma reduced, on tergite 2 yellowish and covering less than half its length.

Mummy. Length 6.7–8.0 mm in males and 7.3–10.0 mm in females, mottled gray and yellowish, with lateral bands more or less defined mottled dark brown and brown, thorax compact and wrinkled, exit hole irregular, located postero-dorsally between anal and abdominal prolegs.

#### Type material.

Type-locality: ECUADOR, Napo Province, Yanayacu Biological Station, Beat 10A, YY-58947, S00°35.9', W77°53.4', 2163 m, cloud forest, September 5, 2011.

Type-specimen: Holotype female and mummy, point mounted separately. Top label: “ECUADOR: Napo Province / Yanayacu Biological Station / S00°35.9', W77°53.4’ 2163m / CAPEA – NSF-BSI-07-17458 / (hand written) July 2011 / 58947; back (hand written): “em. 5 Sept 2011”. (UWIM)

Paratypes (UWIM): 12 females and 8 males, same data as holotype, except different dates. *Females*: August 15, 2007, YY-22975; August 20, 2009 YY-40348; December 21, 2009, YY-43751; April 22, 2010, YY-46509; May 4, 2010, YY-46887; May 12, 2010, YY-47603; July 8, 2010, YY-48837; August 13, 2010, YY-49757; August 25, 2010, YY-50103; November 12, 2010, YY-52466; June 14, 2011, YY-56911; June 16, 2011, YY-56954. *Males*: December 21, 2009, YY-43737; April 1, 2010, YY-45833; April 19, 2010, YY-46665; May 17, 2010, YY-47596; August 7, 2010, YY-49712; August 9, 2010, YY-49716; August 27, 2010, YY-50307; June 14, 2011, YY-56894.

#### Biology.

All specimens were reared from the same Geometridae host caterpillar morphospecies (“linea blanca en la espalda chusquea”) feeding on bamboo, *Chusquea scandens* (Poaceae). Consistent morphology of mummies and rearing data of caterpillars support a single host species for this parasitoid, most frequently sampled between July and August. Most host caterpillars collected were in the 3^rd^ larval instar, but one was in 4^th^ and one in 2^nd^ instar. Time span between host mummification and adult emergence varying in days from: 19–35 for females, and 16–26 for males.

#### Discussion.

This species belongs to *gastritor*/*circumscriptus* species-group. It is similar to *Aleiodes atripileatus*, differing from it by having the occipital carina complete (interrupted at vertex in *Aleiodes atripileatus*), smooth area on mesopleuron (granulate–coriaceous in *Aleiodes atripileatus*), 43 antenomeres (maximum 39 in *Aleiodes atripileatus*), longitudinal carina complete on T1–T3 (incomplete in *Aleiodes atripileatus*), and one basal light spot on T1 and one on T2 (absent in *Aleiodes atripileatus*). This species is also very similar to *Aleiodes nubicola* sp. n. The most evident character to distinguish these species being the color patterns on meso- and metasoma. *Aleiodes cacuangoi* sp. n. have a mostly black metasoma dorsally, with one small to tiny basal white spot on T1 and a finger like mid-basal white spot on T2, the size of the spot on T2 varies from 1/3 to 2/3 of the tergite length. In *Aleiodes nubicola* sp. n. the metasoma varies from entirely black to black with apical whitish spots, these spots are larger on apical terga and extends to T4 in females, but one male paratype has the spots extending from the apical terga throughout apex of T1. There are not apical spots in none of the terga in *Aleiodes cacuangoi* sp. n., while in *Aleiodes nubicola* sp. n. there is not basal white spot on T1. The mesosoma in *Aleiodes cacuangoi* sp. n. is entirely black except for some reddish brown–brown stripe on mesopleuron, which is distinctly lighter close to mid coxal insertion. *Aleiodes nubicola* sp. n. have similar color pattern but with a postero-median square on mesoscutum and scutellum medially orangish. In *Aleiodes cacuangoi* sp. n. the head is mostly yellowish with occiput and vertex medially always black, the face varies from pale yellow to dark brown, and temples and gena are pale yellow to brownish orange. In *Aleiodes nubicola* sp. n. the head is mostly dark brown with a crescent moon-shaped honey yellow area on temples, bordering eyes, and the color of gena is variable. Mummies of *Aleiodes atripileatus*, *Aleiodes cacuangoi* sp. n., and *Aleiodes nubicola* sp. n. are very distinctive: *Aleiodes cacuangoi* sp. n. mummies are mottled gray and yellowish with more or less defined dark brown lateral stripes, *Aleiodes atripileatus* mummies are black with extended anal prolegs and head yellow, and *Aleiodes nubicola* sp. n. mummies are entirely brown and gradually narrowing anteriorly.

#### Etymology.

The species is named in honor to Dolores Cacuango, for her pioneering, outstanding brave efforts for the indigenous rights in Ecuador.

**Figures 25–30.**
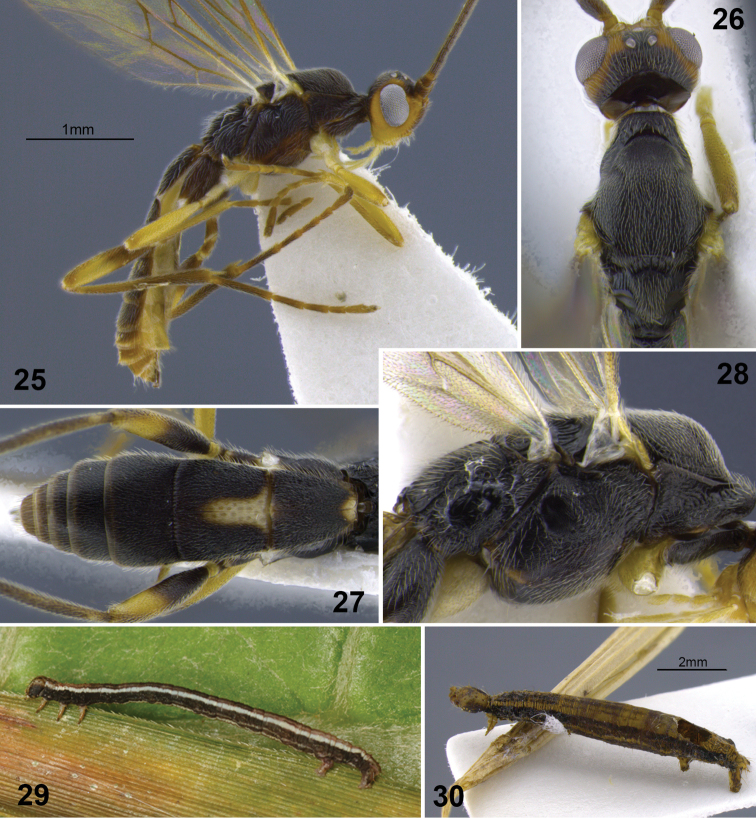
*Aleiodes cacuangoi* sp. n. **25** habitus **26** head and mesonotum, dorsal **27** metasoma, dorsal **28** mesosoma, lateral **29** host larva (Geometridae) **30** host mummy after parasitoid emergence.

### Aleiodes capillosus

Townsend, 2009

http://species-id.net/wiki/Aleiodes_capillosus

Figure 2

#### Diagnosis.

Body length 5.6 mm; antenna with 42 segments; head color black, with small brown spot at gena; malar space 1.8× basal width of mandible, ocelli small, ocell–ocular distance 2.0× width of lateral ocellus; occipital carina completely absent; mesopleuron mostly smooth, epicnemial carina absent; apex of hind tibia without comb of flattened setae; propodeum smooth, without median carina; metasomal tergite 1 white with a black oval spot medially, reminder terga mostly black, laterally white; ovipositor sheaths about 2/3 of the hind basitarsus length.

#### Additional characters.

Last flagellomere lanceolate; mesoscutum with carina on posterior margin varying from present only in front of scutellar sulcus to completely absent; scutellar sulcus with one to five incomplete carina; fore wing vein 1M slightly to moderately curved at base; hind wing vein 2-1A present and relatively long, vein m-cu present and pigmented but never tubular, interstitial to vein r-m; ovipositor sheaths about 2/3 as long as hind basitarsus.

#### Morphological variation.

The non-type material fits well in original description, but most of the mummies are considerably lighter.

#### Material.

Type material examined. (UWIM)

Non-type material: 2 females and 2 males, ECUADOR, Napo, Yanayacu Biological Station, Napo Province, S00°35.9', W77°53.4', 2163 m; voucher numbers: ♀, YY-53697: Geometridae on *Piper* sp1 (Piperaceae) [probably collected as mummy]; ♂, YY-40671: Geometridae on *Alnus acumiata*; ♀, YY-40675: Geometridae on *Alnus acumiata*; ♂, YY-40655: Geometridae on *Alnus acumiata*.

#### Biology.

*Aleiodes capillosus* has been reared from unidentified Geometridae caterpillars on *Diplazium vesiculosum* (Dryopteridaceae). The non-type specimens were also reared from Geometridae, but on *Alnus acumiata* (Betulaceae) and *Piper* sp. (Piperaceae). Feeding on these plant species by the geometrid caterpillars is not confirmed.

#### Distribution.

Known only from the type locality, YBS, Napo province, ECUADOR, at 2,163 meters elevation.

#### Discussion.

*Aleiodes capillosus* is very similar to *Aleiodes marilynae* sp. n. (for a discussion on distinguishing characters see discussion section under *Aleiodes marilynae* sp. n.). These are the only two species in the *gressitti* species-group from Neotropical Region. As well as the morphological similarities in the adult parasitoids, the host mummies of *Aleiodes capillosus* and *Aleiodes marilynae* are very similar. Both produce a relatively swollen mummy with a strongly shrunken thorax, where it is attached to a leaf or branch in a distinct angle.

### Aleiodes colberti sp. n.

http://zoobank.org/62EFAC20-EA55-4035-A4F9-5063A52DE15F

http://species-id.net/wiki/Aleiodes_colberti

**Figures 31–35.**
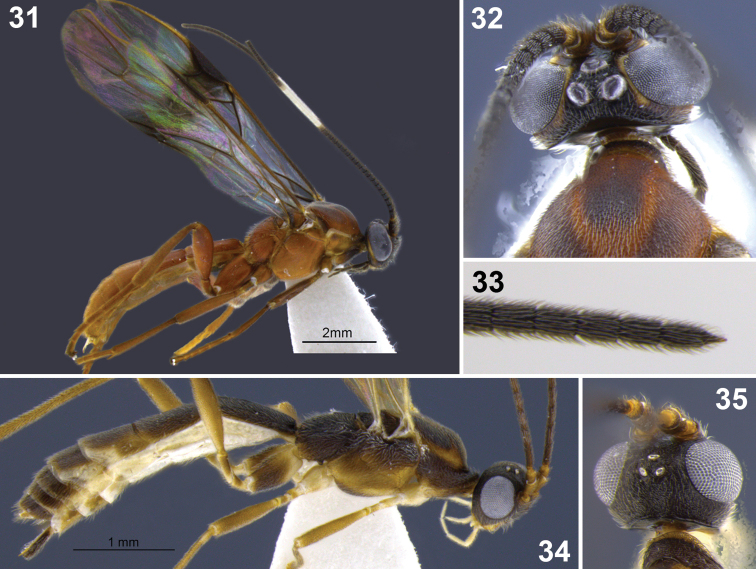
*Aleiodes colberti* sp. n. **31** habitus **32** head and mesoscutum, dorsal **33** apical flagellomeres **34, 35** *Aleiodes delicatus* sp. n. **34** habitus **35** head dorsal.

**Figures 36–37.**
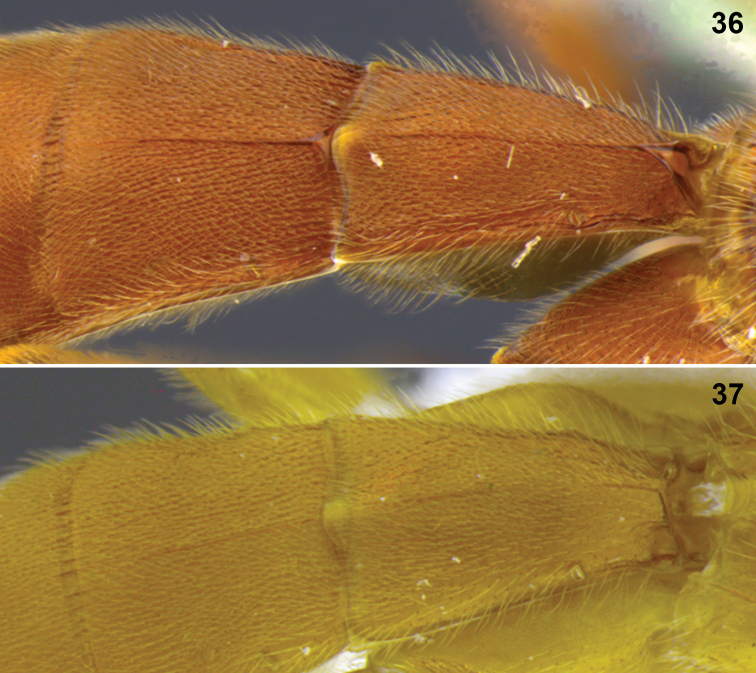
Metasomal terga 1–2. **36** *Aleiodes colberti* sp. n. **37** *Aleiodes luteosicarius* sp. n.

Figures 31–33 , 36 , 121

#### Description of the holotype.

Male (holotype). Body length 8.8 mm; antenna length 8.5 mm; fore wing length 8.3 mm.

Color. Mostly reddish brown. Head mostly dark brown to black; yellowish stripe around eyes on frons, temple and gena; palp dark brown. Antenna black with white middle band between flagellomeres 20–29 (+/- 1). Mesosoma mostly reddish brown; pronotum brown dorsally, laterally irregularly yellowish brown; propleuron brown, lighter posteriorly; mesoscutum infuscate except on notauli region; scutellum brown medially and postero-laterally. Wings weakly infuscate with dark band just bellow stigma; veins brown and stigma dark brown; tegula brown.

Head. 54 antennomeres, flagellomeres as long as wide, except apical 1/3 and basal 1/6, slightly longer than wide, apical flagellomere with distinctly pointed apex; malar space very short, 0.5× basal width of mandible and 1/6 of eye height; temple very narrow, in dorsal view eyes 5.5× longer than temples; occipital carina complete thought irregular dorsally, ventrally barely reaching hypostomal carina; oral space small and circular, diameter about equal to basal width of mandible; clypeus weakly swollen; ocelli extremely large, ocell–ocular distance 1/8 diameter of lateral ocellus; face, gena and temples transverse rugose–costate, with mid-longitudinal ridge just bellow toruli, face granulose medially, clypeus granular–rugose, frons smooth and excavated, bordered by “W-shaped” carina, vertex coarsely granulate.

Mesosoma. Sculpturing mostly opaque granular; pronotum with median scrobiculate line; epicnemial carina incomplete dorsally; propodeum laterally with few wrinkles, mid-longitudinal carina on anterior 1/2, posterior half with irregular wrinkles; metapleuron rugose posteriorly; notauli virtually absent, only indicated anteriorly and meeting rugose depressed area posteriorly; posterior margin of mesoscutum bordered by short carina just in front of scutellar sulcus; scutellar sulcus with five complete and well defined carina.

Wings. Fore wing: stigma 4× longer than high; vein r about 0.7× vein 2RS, as long as vein RS+Mb, and 0.7× vein m-cu; vein 3RSa 0.64× vein 3RSb, and 0.85× vein 2M; vein 1CUa 1.33× vein 1cu-a; vein 1CUb 2.25× vein 1CUa; vein 1M moderately curved at basal half. Hind wing: vein m-cu absent; vein M+CU slightly shorter 1M; 1M about 2× length of r-m; vein RS well pigmented, almost straight, gradually diverging from wing margin; vein M dark brown, well pigmented; vein 2-1A present and relatively long.

Metasoma. T1 and T2 granular–striate, longitudinal carina complete on T1 and T2; remainder visible terga granular; T4–T6 with small circular median dorsal pits, these terga densely setose except on median line, pubescence around the bare line directed to it; T1 length 2× apical width.

Legs. Tarsal claws pectinate, with 6–7 thick bristles, and distinct gap between apical claw and basal pectination; hind basitarsus 1.7× longer than inner apical spur of hind tibia.

Female unknown.

#### Type material.

Type-locality: ECUADOR, Napo Province, Yanayacu Biological Station, Macucoloma trail, S00°35.9', W77°53.4', 2163 m, cloud forest, March 2–5, 2006, S.R. Shaw col.

Type-specimen: Holotype male, point mounted. Top label: “ECUADOR: Napo Province / Yanayacu Biological Station / S00°35.9', W77°53.4’ 2163m / 2-5 March 2006, S.R. Shaw / Ex. Yellow pan trap”. (UWIM)

#### Discussion.

*Aleiodes colberti* sp. n. belongs to the *pulchripes* species-group. This species can be distinguished from other New World species of this group by the infuscate band on the forewing just below the pterostigma, and the white middle band on antenna. Despite these characters, it is similar to *Aleiodes earinos* Shaw, 1997, differing from which in its mostly black head and some dark stains on the mesoscutum and scutellum, as compared with the unicolored body in *Aleiodes earinos*. The pattern of wing veins resembles *Aleidoes arizonensis* Marsh & Shaw, 1997, mainly by the very long second submarginal cell. The infuscation below the stigma is also present in the Cuban and Costa Rican species *Aleiodes pedalis* Cresson, 1869, but *Aleiodes pedalis* also has distinct infumation apically, not present in *Aleiodes colberti* sp. n., as well as the apical hind tibia black, as opposed to reddish in *Aleiodes colberti* sp. n. In the key to New World *pulchripes* species (S. Shaw et al. 1997), *Aleiodes colberti* does not run easily to any of the described species. Considering the body color it will be forcibly run to *Aleiodes notozophus* Marsh & Shaw, 1997, but differs from that species by the large gap between apical claw and basal pectination, which is absent in *Aleiodes notozophus*. The male of *Aleiodes colberti* sp. n. has small setose pits on terga 4–6 (4–7 in *Aleiodes notozophus*). Disregarding the presence of the pits it will run to *Aleiodes vaughani* Muesebeck, 1960, but the ocelli are larger in the new species.

#### Etymology.

This species is named after Stephen Tyrone Colbert, an American comedian, political satirist, writer, actor, and host of *The Colbert Report*.

### Aleiodes delicatus sp. n.

http://zoobank.org/2C718F94-CE3D-4120-BD76-BD3F2FE1713D

http://species-id.net/wiki/Aleiodes_delicatus

Figures 34 , 35

#### Description of holotype.

Female (holotype). Body length 5.4 mm; antenna length 7.3 mm; fore wing length 4.5 mm.

Color. Mostly dark brown; legs pale brown but fore and mid coxae and trochanter whitish, hind coxa mostly dark brown, whitish on basal 1/4; all palp whitish; scape and pedicel, notauli and ventral border of mesoscutum, and mandibles yellowish; scutellum brown mid-anteriorly; propleuron pale brown, anteriorly darker; mesopleuron pale brown laterally, ventrally whitish with a roughly defined inverted “heart-shaped” honey brown infuscation; metasomal terga 3 and 4 slightly lighter than remainder metasoma and with whitish lateral borders; metasoma ventrally white except for last two sternites mostly light brown. Wing veins brown, but basally whitish, stigma pale brown, tegula white.

Head. Antenna with 45 segments, flagellomeres roughly 2.0× as long as wide, apical flagellomere with small pointed apex; malar space wide, about 1.6× basal width of mandible, 0.6× eye height; in dorsal view eyes 2.4× longer than temples; occipital carina complete dorsally, well defined laterally and meeting hypostomal carina; oral space small and circular, maximum width about as long as basal width of mandible; clypeus not swollen; ocelli small, ocell–ocular distance 2× diameter of lateral ocellus; maxillary palp not swollen; head surface sculpture coarse granulate, occiput smooth and shining, frons not excavated, higher face with well defined mid-longitudinal ridge.

Mesosoma. Sculpturing mostly coarse granulate; pronotum foveate; propodeum rugulose with granular background, mid-longitudinal carina present on anterior 1/3; notauli very shallow and narrow, with few crenulae anteriorly; posteriorly meeting on almost flat and weakly rugose area; posterior margin of mesoscutum bordered by carina; scutellar sulcus long, with three strong but incomplete carina.

Wings. Fore wing: stigma 4.5× longer than high; vein r about 1.3× vein 2RS, 1.4× vein RS+Mb, and 1.0× vein m-cu; second submarginal cell small and trapezoidal, vein 3RSa 0.28× vein 3RSb, and 0.77× vein 2M; vein 1CUa 1.6× vein 1cu-a; vein 1CUb 2.7× 1CUa; vein 1M weakly curved on basal half. Hind wing: vein m-cu absent; vein M+CU 0.85× 1M; 1M about 2.7× length of r-m; vein RS sinuate at middle, then only slightly diverging from wing margin; vein M dark brown, well pigmented; vein 2-1A absent.

Legs. Hind tibia with comb of modified setae; tarsal claw simple, not pectinate; inner apical spur on hind tibia extremely short, hind basitarsus 5× longer than inner spur.

Metasoma. T1, T2 and basal 2/5 of T3 finely rugose–striate with coarse granulate background, longitudinal carina present along T1 and T2; apical 3/5 of T3 coarse granulate; remainder visible terga granular coriaceous; ovipositor sheaths as long as hind tarsomere II, and 0.47× length hind basitarsus; T1 long and narrow, 1.6× longer than its apical width.

Male. Unknown.

#### Type material.

Type-locality: ECUADOR, Napo Province, Yanayacu Biological Station, Macucoloma trail, S00°35.9', W77°53.4', 2163 m, cloud forest, August 15, 2006, A. Townsend col.

Type-specimen: Holotype female, point mounted. Top label: “ECUADOR: Napo Prov. / Yanayacu Biological Station / 15-Aug-2006 Macucaloma trail / Maxi net 2163m. A. Townsend”. (UWIM)

#### Discussion.

*Aleiodes delicatus* sp. n. belongs to *seriatus* species-group. It is very similar to *Aleiodes pectoralis* (Ashmead, 1894) (=*sanctivicentensis* Shenefelt, 1975), from St. Vincent, and *Aleiodes akidnus* Marsh & Shaw, 1998, from Florida – USA, by the very small ocelli and the wing venation, remarkably the short second submarginal cell of the forewing, and also the longitudinal carina incomplete on propodeum. *Aleiodes delicatus* sp. n. is mostly dark brown with some lighter regions on mesoscutum and mesopleuron (legs also lighter and metasoma ventrally white), being the color pattern very similar to *Aleiodes pectoralis*, while *Aleiodes akidnus* is entirely honey yellow with black stemmaticum; however, the gena of *Aleiodes delicatus* sp. n. is dark brown, compared to light yellowish in *Aleiodes pectoralis*. Though short, the second submarginal cell is not almost square as in *Aleiodes akidnus* and *Aleiodes pectoralis*. The vein r is 0.75× vein 3RSa in *Aleiodes delicatus* sp. n., but in *Aleiodes akidnus* and *Aleiodes pectoralis* r is slightly longer than 3RSa. In *Aleiodes delicatus* sp. n. the longitudinal carina is absent on metasomal tergite 3, but it is complete in *Aleiodes akidnus* and present on basal half in *Aleiodes pectoralis*, and the first metasomal tergite is 1.6× longer than apical width but roughly as long as wide in *Aleiodes akidnus* and about 1.3× in *Aleiodes pectoralis*.

#### Etymology.

From the Latin, meaning delicate.

### Aleiodes dyeri sp. n.

http://zoobank.org/6319072D-5E3A-4963-B0E2-135DBA18F0E4

http://species-id.net/wiki/Aleiodes_dyeri

Figures 38–40 , 122

#### Description of holotype.

Female (holotype). Body length 6.1 mm; antenna length 6+ mm; fore wing length 5.5 mm.

Color. Head honey yellow, ocellar triangle black; antenna dark brown, scape slightly lighter. Mesosoma honey yellow, some lighter parts on metapleuron and dorsal mesopleuron. Fore leg honey yellow, tarsi slightly darker but 5^th^ tarsomere brown. Mid leg with same pattern of fore leg, but all tarsi brown; coxa, trochanter and trochantellus whitish, coxa with dark lateral stains. Hind leg: coxa mostly dark brown, basal third pale yellow; trochanter and trochantellus white with infuscate stains dorsally; femur black except for narrow basal whitish band; tibia and tarsi dark brown, tibial spurs honey brown. Metasoma black dorsally, ventrally white. Wings hyaline basally, becoming weakly infuscate apically; veins dark brown.

Head. Antenna with 53 antennomeres, flagellomeres roughly 2.0× as long as wide; malar space short, just slightly longer than basal width of mandible, and 0.3× eye height; eyes large, in lateral view temple very narrow, in dorsal view eyes 4.7× longer than temples; occipital carina incomplete, not meeting dorsally and curving toward lateral ocelli, well defined laterally and meeting hypostomal carina; oral space small and circular, maximum width slightly smaller than basal width of mandible; clypeus slightly swollen; ocell–ocular distance about 0.7× diameter of lateral ocellus; maxillary palp not swollen; head surface sculpturing shining granulate, occiput smooth and shining, frons also smooth with small weak concentric wrinkles; higher face with a small longitudinal ridge and transverse rugosity directed to it; frons excavated with excavation bordered by a weak “W-shaped” carina.

Mesosoma. Sculpturing shining granulate; pronotum foveate; propodeum coarsely shining granular with complete mid-longitudinal carina; notauli shallow and crenulate anteriorly, posteriorly meeting on depressed rugose area; posterior margin of mesoscutum bordered with complete carina; scutellar sulcus with median carina plus three pairs of almost complete lateral carina.

Wings. Fore wing: stigma 3.5× longer than high; vein r 0.75× vein 2RS, slightly longer than vein RS+Mb, and 0.7× vein m-cu; vein 3RSa 0.55× vein 3RSb, and 0.9× vein 2M; vein 1CUa 3× vein 1cu-a; vein 1CUb almost as long as vein 1CUa; vein 1M evenly slightly curved. Hind wing: vein m-cu distinct, pigmented and apparently tubular, distinctly antefurcal; M+CU 1.5× 1M; vein 1M short, 0.85× vein r-m; RS almost parallel to wing margin on basal 1/3 then slightly sinuate; vein M dark brown, well pigmented; vein 2-1A absent.

Legs. Hind tibia with comb of modified setae; tarsal claw pectinate, bristles relatively long and tightly arranged, with a short gap between pectination and claw base; hind basitarsus 3.4× longer than inner apical spur of hind tibia.

Metasoma. T1, T2 and basal 2/5 of T3 rugose costate, longitudinal carina present along this sculpturing; remainder visible terga granular coriaceous; ovipositor sheaths about as long as tarsomere II; T1 length 1.36× its apical width.

Male unknown.

Mummy. Length 8.5 mm, reddish brown (similar to a dipteran Brachycera puparium), most setae fell apart, exit hole irregular, located postero-dorsally.

#### Type material.

Type-locality: ECUADOR, Napo Province, Yanayacu Biological Station, YY-53568, S00°35.9', W77°53.4', 2163 m, cloud forest, February 15, 2011.

Type-specimen: Holotype female and mummy, point mounted separately. Top label: “ECUADOR: Napo Province / Yanayacu Biological Station / S00°35.9', W77°53.4', 2163m / CAPEA - NSF-BSI-07-17458 / (hand written) Dec. 2010 / YY-53568; back (hand written): “15-Feb-2011”. (UWIM)

#### Biology.

Reared from a species of *Holophaea* Druce (Erebidae) caterpillar (YY-53568), feeding on *Diplazium costale* var. *robustum* (Dryopteridaceae). Time span, from host mummification until adult emergence, about 5 weeks.

#### Discussion.

*Aleiodes dyeri* sp. n. belongs to the *seriatus* species-group, where it resembles *Aleiodes greeneyi*, because of the dorsally incomplete occipital carina. It can be distinguished from *Aleiodes greeneyi* by the honey yellow mesosoma (dorsally black in *Aleiodes greeneyi*), the fore wing vein r 1.5× longer than RS+Mb (1.0× in *Aleiodes greeneyi*), and the hind wing vein r-m longer than 1M (shorter in *Aleiodes greeneyi*). *Aleiodes dyeri* sp. n. is similar to *Aleiodes longikeros* sp. n. in color patterns. These two species differ in the sculpturing of mesopleuron, being entirely granular in *Aleiodes dyeri* sp. n. but with a smooth elevated area in *Aleiodes longikeros* sp. n. The hind wing vein 1M is shorter than r-m in *Aleiodes dyeri* sp. n., as opposed to being 2.4× longer in *Aleiodes longikeros* sp. n., and the shape of fore wing vein 1M is weakly sinuate in *Aleiodes dyeri* sp. n., as compared with strongly curved in *Aleiodes longikeros* sp. n. Within the Nearctic species, *Aleiodes dyeri* sp. n. is more similar to *Aleiodes preclarus* Marsh & Shaw, 1998, from which it differs in the entire yellowish head but ocellar triangle black (several black spots in *Aleiodes preclarus*), wing veins mostly dark brown except fore wing veins M+CU and 1A, and hind wing veins 1M and M+CU proximally yellowish (pterostigma and fore wing vein C+SC+R with yellow spots in *Aleiodes preclarus*), and frons smooth (porcate in *Aleiodes preclarus*).

#### Comments.

The antenna tips of the type specimen have a withered aspect, which makes impossible to measure the exact length of the antenna or describe the shape of the apical flagellomere.

#### Etymology.

This species is named after Dr. Lee Dyer, of the University of Nevada (Reno), the lead investigator of the *Caterpillars and Parasitoids of the Eastern Andes of Ecuador* (CAPEA) project.

**Figures 38–44.**
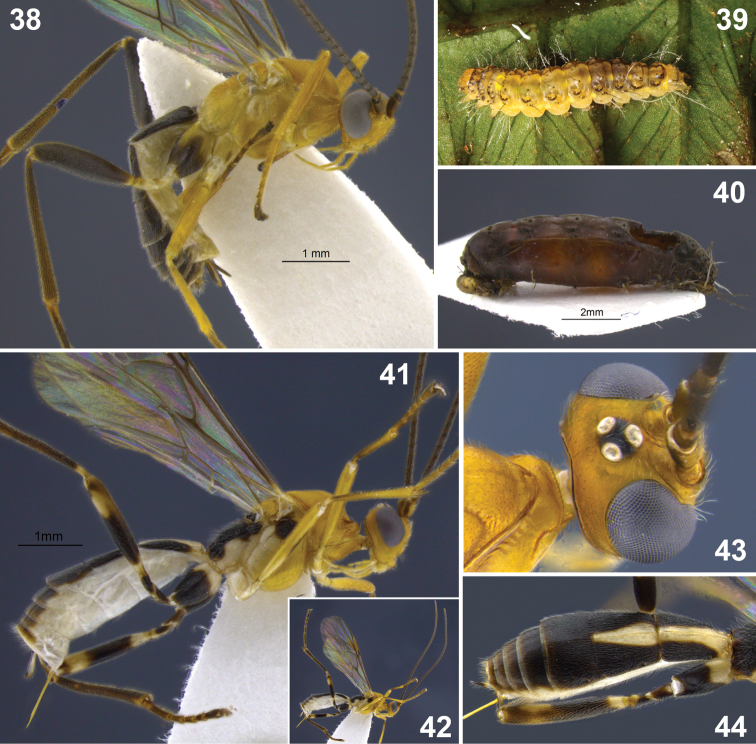
*Aleiodes dyeri* sp. n. **38** habitus **39** host larva, *Holophaea* sp. (Erebidae) **40** host mummy after parasitoid emergence **41–44** *Aleiodes elleni* sp. n. **41**, habitus **42** whole specimen habitus **43** head dorsal **44** metasoma, dorsal.

### Aleiodes elleni sp. n.

http://zoobank.org/B69D7595-D459-4032-B4BA-FC7CD67DFE48

http://species-id.net/wiki/Aleiodes_elleni

Figures 41–44

#### Description of holotype.

Female (holotype). Body length 6.1 mm; antenna length 7.1 mm; fore wing length 5.5 mm.

Color. Head yellow, ocellar triangle dark brown; antenna dark brown–black; scape, pedicel and first flagellomere proximally honey brown; scape darker dorsally; mesosoma yellow, anterior corner of mesopleuron, metanotum propodeum and metapleuron dorsally dark brown–black, mesopleuron light yellow–whitish postero-dorsally; fore and mid leg coxa, trochanter and trochantellus whitish, darkening toward apex, 5^th^ tarsomere honey brown, but tibia darker basally; mid trochanter, trochantellus and femur with infuscate marking on inner side; hind coxa basal 1/3 white, apical 2/3 black; hind trochanter dark brown; hind trochantellus black with ventral white stripe; hind femur ventrally from base to apex with alternating bands: about 1/6 white, 1/2 dark brown, 1/4 pale yellow and 1/12 brown; dorsally only the basal white band well defined; tibia dark brown with basal pale yellow band; tibial spurs honey brown; tarsi brown. Metasomal terga black; median white stripe throughout length of terga 1 and 2; ovipositor sheaths dark brown apically, base whitish. Wings weakly infuscate; stigma brown; most veins dark brown; vein C+SC+R darker (extreme base whitish) connecting to a black parastigma with light brown central spot.

Head. Antenna with 45 segments, flagellomeres roughly 2.0× as long as wide, apical flagellomere with short “bottle-nipple”-shaped tip; malar space moderate, about 1.3× basal width of mandible, 0.4× eye height; in dorsal view eyes 2.5× longer than temples; occipital carina complete dorsally, well defined laterally and meeting hypostomal carina; oral space small and circular, maximum width equal to basal width of mandible; clypeus slightly swollen; ocell–ocular distance about as long as diameter of lateral ocellus; maxillary palp not swollen; head surface sculpturing shining granulate, occiput smooth and shining, frons also smooth with weak concentric wrinkles; frons excavated, excavation bordered by a weak “W-shaped” carina, but stronger laterally.

Mesosoma. Sculpturing shining granulate; pronotum foveate; propodeum rugulose with granular background and complete mid-longitudinal carina. Meso- and metapleuron surface rugose on dorsal dark brown areas; notauli shallow and crenulate anteriorly, posteriorly meeting on depressed rugose area; posterior margin of mesoscutum bordered with complete carina; scutellar sulcus with complete median carina plus two pairs of poorly defined lateral carina.

Wings. Fore wing: stigma 3.4× longer than high; vein r about 0.8× vein 2RS, 1.2× vein RS+Mb, and 0.7× vein m-cu; vein 3RSa 0.48× vein 3RSb, and 0.9× vein 2M; vein 1CUa 3.2× vein 1cu-a; vein 1CUb about as long as vein 1CUa; vein 1M slightly curved on basal half. Hind wing: m-cu is distinct, apparently tubular, distinctly antefurcal; vein M+CU about 2× longer than vein 1M; 1M about 0.8× length of r-m; vein RS almost straight, slightly diverging from wing margin; vein M dark brown, well pigmented; vein 2-1A present as a very short stub.

Legs. Hind tibia with comb of modified setae; tarsal claw simple, not pectinate; hind basitarsus 3× longer than inner apical spur on hind tibia.

Metasoma. T1, T2 and basal 2/5 of T3 granular–striate, longitudinal carina present along this sculpturing; remainder visible terga granular coriaceous; ovipositor sheaths about 1/2 length of hind basitarsus; T1 1.4× longer than its apical width.

Male. Antenna with 42–43 segments; metasoma narrower, T1 about 1.5–1.6× longer than its apical width; ocell–ocular distance shorter, 0.7× diameter of lateral ocellus; stigma narrower, 4.2× longer than high; one male has the white median stripe on metasoma interrupted on posterior half of tergite 2.

#### Type material.

Type-locality: ECUADOR, Napo Province, Yanayacu Biological Station, Macucoloma trail, S00°35.9', W77°53.4', 2163 m, cloud forest, November 5–12, 2007, J. Simbaña col.

Type-specimen: Holotype female. Top label: “ECUADOR: Napo Province / Yanayacu Biological Station / S00°35.9', W77°53.4’ 2163m / 5-12 Nov. 2007, J. Simbaña / Macucoloma trail, Malaise trap / NSF-BSI-07-17458, S.R. Shaw”; second label: “SRS-00034”. (UWIM)

Paratypes, 2 males (UWIM), same locality as holotype, different collection methods and dates: 1♂, June 18, 2010, yellow pan trap, S.R. Shaw col.; 1♂, May 12, 2011, U.V. light, N. Zitani col.

#### Discussion.

This species belongs to the *seriatus* species-group. *Aleiodes elleni* sp. n. is the only newly described species in this group with a strong, complete occipital carina on vertex. This character is present in two other Neotropical species: *Aleiodes scriptus* (from Brazil) and *Aleiodes nebulosus* (from Ecuador) from which *Aleiodes elleni* sp. n. differs by having the hind wing vein RS straight. *Aleiodes elleni* sp. n. also differs from *Aleiodes scriptus* by having the sculpturing of metasoma rugose–costate, as compared with widely costate in *Aleiodes scriptus*. It differs from *Aleiodes nebulosus* mostly in color patterns: pronotum yellow (white in *Aleiodes nebulosus*), apical 2/3 of hind coxa, propodeum, metanotum, metapleuron dorsally and mesopleuron on antero-dorsal corner black (white in *Aleiodes nebulosus*), and white medial marking on metasoma extending throughout tegite 1 and 2 (only anteriorly on tergite 1 in *Aleiodes nebulosus*). *Aleiodes elleni* sp. n. also differs from *Aleiodes nebulosus* in having the 2^nd^ submarginal cell in fore wing long and rectangular (short and trapezoidal in *Aleiodes nebulosus*), 2RS and 3RSa forming a right angle (obtuse in *Aleiodes nebulosus*), and vein r less than half length of vein 3RSa (0.85× in *Aleiodes nebulosus*), wings hyaline (wings moderately infuscate in *Aleiodes nebulosus*), and frons with lateral ridges (absent in *Aleiodes nebulosus*). The straight vein RS of the hind wing is shared with two other species of this species group: *Aleiodes frosti* sp. n. and the Nearctic species *Aleiodes femoratus* Cresson, 1869; however, *Aleiodes elleni* sp. n. differs from *Aleiodes frosti* sp. n. by its smaller ocelli and relatively shorter petiole, and from both by the mostly black and medially whitish metasoma (yellowish in *Aleiodes frosti* sp. n. and *Aleiodes femoratus*). It also differs from *Aleiodes femoratus* by the smooth frons, as compared with porcate frontal sculpture in *Aleiodes femoratus*.

#### Etymology.

This species is named after the American actress, comedian, and television host Ellen Lee DeGeneres.

### Aleiodes falloni sp. n.

http://zoobank.org/63DE928D-1CA0-4B80-B9B7-FDC81B5D4AC3

http://species-id.net/wiki/Aleiodes_falloni

Figures 45–46 , 123

#### Description of holotype.

Female (holotype). Body length 6.9 mm; antenna length 7.6 mm; fore wing length 6.0 mm.

Color. Body gold–honey brown to orangish brown. Flagellum dark brown, scape and pedicel laterally brown; ocellar triangle black; tarsal claws brown; metasoma ventrally, mandibles, fore and mid coxa light yellowish. Wings weakly infuscate; veins dark brown but C+SC+R honey brown; stigma and vein R1 pale yellow–pale honey brown.

Head. Antenna with 50 antennomeres, flagellomeres roughly 2.0× as long as wide, apical flagellomere with short pointed apex; malar space as long as basal width of mandible, and 0.35× eye height; in dorsal view eye height 2.2× temple; occipital carina incomplete dorsally (but not curved toward vertex), otherwise complete but not touching hypostomal carina; oral space small and circular, maximum width equal to basal width of mandible; clypeus bulging; ocell–ocular distance 0.86× diameter of lateral ocellus; maxillary palp not swollen. Head surface sculpturing finely granulate, higher face with small longitudinal ridge and transverse rugosity directed to it, occiput smooth and shining.

Mesosoma. Sculpturing mostly granulate; pronotum foveate laterally; mesopleuron rugose on anterior corner; propodeum rugose–granulate, posteriorly with diverging and some transverse wrinkles and weakly rugose laterally, with mid-longitudinal carina present on anterior 2/3; notauli present anteriorly, wide and shallow, posteriorly disappears in a depressed area with striations running antero-laterally from mid-posterior region; posterior margin of mesoscutum with short carina, just anterior to scutellar sulcus; scutellar sulcus with median carina plus two pairs of well defined lateral carina.

Wings. Fore wing: stigma 3× longer than high; vein r 0.55× vein 2RS, as long as vein RS+Mb, and 0.5× as long as vein m-cu; vein 3RSa about 0.5 times vein 3RSb, and as long as vein 2M; vein 1CUa 2× vein 1cu-a; vein 1CUb 1.8× vein 1CUa; vein 1M evenly slightly curved. Hind wing: m-cu indicated as short pigmented vein antefurcal to vein r-m (in this species and others, the vein m-cu is very short but is also indicated by a slight bent on vein M, were these veins meet); vein M+CU about 1.4× 1M; vein 1M 1.4× vein r-m; vein RS smoothly curved at middle; vein M straight; vein 2-1A absent.

Legs. Hind tibia without comb of modified setae; tarsal claw pectinate at base, with a distinct gap between apical claw and basal pectination, pectin with 5–6 bristles; hind basitarsus 3× longer than inner apical spur on hind tibia; few rugositie dorso-laterally on outer side of hind coxa.

Metasoma. T1–T2 striated; remainder terga coriaceous; mid longitudinal carina complete from T1 throughout T2; ovipositor sheaths slightly shorter than hind tarsomere II, parallel sided and sharpened apically, setae longer pre-apically, longest setae about 1.5× longer than maximum width of sheaths; T1 slightly longer than apical width.

Variation. Antenna with 49–53 segments; some specimens with weak striation on basal 1/5 of metasomal tergite 3; scutellar sulcus with 3 to 5 well defined carina; one specimen with hind wing vein m-cu interstitial.

Male unknown.

#### Type material.

Type-locality: ECUADOR, Napo Province, Yanayacu Biological Station, Macucoloma trail, S00°35.9', W77°53.4', 2163 m, cloud forest, May 1–8, 2007, J. Simbaña col.

Type-specimen: Holotype female. Top label: “ECUADOR: Napo Province / Yanayacu Biological Station / S00°35.9', W77°53.4’ 2163m / 1-8 May 2007, J. Simbaña / Macucoloma trail, Malaise trap / NSF-BSI-07-17458, S.R. Shaw”; second label: “SRS-00028”. (UWIM)

Paratypes. 1♀, same as holotype; 9♀s, same data as holotype but different dates: 5♀s, May 1–10, 2010; 1♀, June 3–13, 2009; 1♀, April 1–8, 2007; 2♀ February 1–8, 2007. (UWIM)

#### Discussion.

This species belongs to *circumscriptus*/*gastritor* species group. It differs from the described species in this group by its mostly honey brown body color. *Aleiodes falloni* sp. n. resembles *Aleiodes speciosus* Townsend, from which it can be distinguished by the entire honey brown body color (mostly black–dark brown dorsally with first tergite white in *Aleiodes speciosus*) and the hind wing vein 2-1A absent (present in *Aleiodes speciosus*). It is very similar to *Aleiodes luteosicarius* sp. n., which belongs to the *pallidator* species-group, especially in color pattern (for distinguishing features see discussion at *Aleiodes luteosicarius* sp. n. section). Morphological distinction between *Aleiodes falloni* sp. n. and *Aleiodes luteosicarius* sp. n. is difficult due to their general resemblance. Separation of specimens in two different entities was supported by comparison of ribosomal COI sequences, resulting in two groups with considerably different genetic information.

#### Etymology.

This species is named after James Thomas Fallon, known as Jimmy Fallon, an American television host, comedian, actor, singer, musician and producer.

**Figures 45–46.**
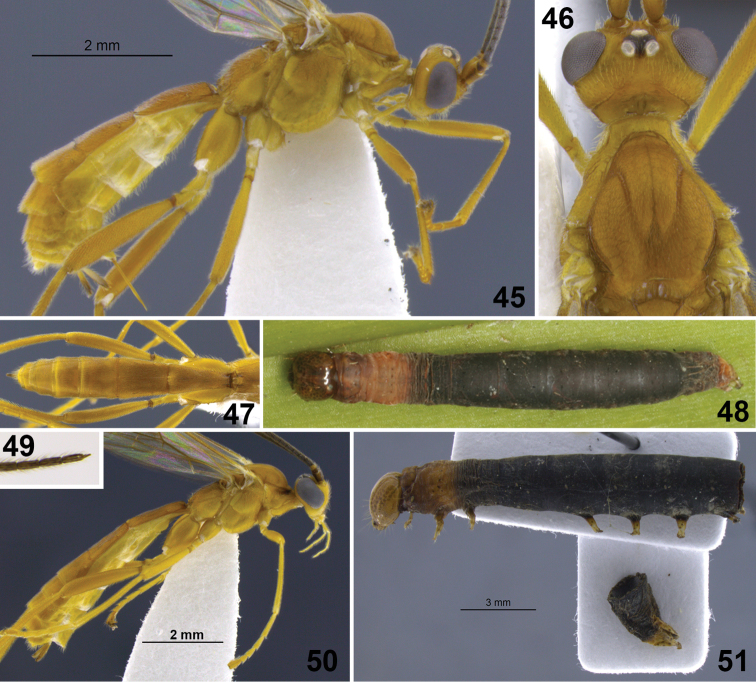
*Aleiodes falloni* sp. n. **45** habitus **46** head and mesonotum, dorsal **47–51** *Aleiodes frosti* sp. n. **47** metasoma, dorsal **48** host mummy, *Scoturopsis* Hering sp. (Notodontidae), before parasitoid emergence **49** apical flagellomeres **50** habitus **51** host mummy after parasitoid emergence.

### Aleiodes frosti sp. n.

http://zoobank.org/41C9635C-F5EF-4949-8822-01B80BEE93D0

http://species-id.net/wiki/Aleiodes_frosti

Figures 47–51

#### Description of holotype.

Female (holotype). Body length 8.1 mm; antenna length 10.7 mm; fore wing length 7.6 mm.

Body color. Yellowish to honey yellow, except for the ocellar triangle and antenna dark brown. Wings tinged yellowish; veins honey brown, parastigma blackish with central yellowish spot.

Head. Antenna 65 antennomeres, antenna 1.3× longer than body, flagellomeres roughly 2× longer than wide, apical flagellomere with long and narrow “bottle-nipple”-shaped apex; malar space short, about as long as basal width of mandible, and about 0.33× eye height; in dorsal view eyes 5× longer than temples; occipital carina incomplete dorsally, well defined laterally and meeting hypostomal carina; oral space small and circular, maximum width equal to basal width of mandible; clypeus large, not swollen; ocelli large, ocell–ocular distance about 0.4× diameter of lateral ocellus; maxillary palp not swollen; head surface sculpture finely shining granulate, occiput smooth and shining; higher face with a small longitudinal ridge and transverse rugosity directed to it; frons polished and excavated, without lateral ridges.

Mesosoma. Sculpturing shining granulate; pronotum with few wrinkles posteriorly and dorso-laterally; mesopleuron with small area at antero-dorsal corner rugose, and some wrinkles dorsally; propodeum longitudinally rugose posteriorly, mid-longitudinal carina almost complete; notauli with few crenulae and shallow anteriorly, meeting on depressed rugose area posteriorly; posterior margin of mesoscutum with complete carina; scutellar sulcus shallow and smooth except for the median carina.

Wings. Fore wing: stigma 5.3× longer than high; vein r 0.7× vein 2RS, 0.7× longer than vein RS+Mb, and 0.5× vein m-cu; vein 3RSa 0.5× vein 3RSb, and as long as vein 2M; vein 1CUa 1.7× vein 1cu-a; vein 1CUb 2.4× vein 1CUa; vein 1M weakly curved at its basal portion; RS+M straight. Hind wing: m-cu present, short and weakly pigmented, interstitial or just antefurcal to r-m; M+CU 1.25× vein 1M; 1M as long as r-m; RS mostly straight and gradually opening from wing margin, slightly bent downward at mid length; vein M straight, dark brown, well pigmented; vein 2-1A present as a short stub.

Legs. Hind tibia with comb of modified setae; tarsal claw simple, not pectinate; hind basitarsus 3× longer than inner apical spur of hind tibia.

Metasoma. T1, T2 and basal 2/3 of T3 rugose–striate, longitudinal carina complete on T1 and almost complete on T2, but not reaching posterior margin; ovipositor sheaths parallel sided and truncate, about as long as hind tarsomere III; metasoma unusually long and narrow, T1 2.2× longer than its apical width.

Variation. Body length about 8.5 mm; antennomeres = 63; ocelli larger, ocell–ocular distance 0.25× diameter of lateral ocellus; hind wing vein 2-1A absent to short.

Male. Body length about 8.5 mm; antenna with 63 segments. Virtually identical to female, but ocell–ocular distance 0.3× diameter of lateral ocellus.

Mummy. Length 14.0 mm, black, thorax brown, head honey brown mottled brown, tubular in shape, thorax compact and wrinkled, exit mode unique within *Aleiodes*: the parasitoid cuts a radial opening at posterior side of the mummy, just behind the hind abdominal prolegs, releasing a “lid” with the anal prolegs.

#### Type material.

Type-locality: ECUADOR, Napo Province, Yanayacu Biological Station, YY-50211, Beat C-16, S00°35.9', W77°53.4', 2163 m, cloud forest, September 5, 2010.

Type-specimen: Holotype female and mummy, point mounted separately. Top label: “ECUADOR: Napo Province / Yanayacu Biological Station / S00°35.9', W77°53.4’ 2163m / CAPEA - NSF-BSI-07-17458 / (hand written) July 2010 / YY-50211; back (hand written): “5-Sep-2010”. (UWIM)

Paratypes, 1 female and 1 male (UWIM), same data as holotype, except: 1♀, 23 May 2008, Yanayacu Road, YY-31409, colección por golpeo / ex. Poaceae *Chusquea scandens*– wasp emerged 02 July 2008; 1♂ YY-46923, beat 638, ex. host plant: Poaceae *Chusquea scandens* parasitoid emerged 10 May 2010.

#### Biology.

Host plant *Chusquea scandens* (Poaceae); host Lepidoptera: *Scoturopsis* Hering sp. (Notodontidae); time span from pupation to emergence: about 5 weeks for females, unknown for the male. The parasitoid cuts a radial opening at posterior side of the mummy releasing a “lid”, comprinsing the anal apex of mummified caterpillar, before emergence. The mummy exit mode of this species is unique for the genus, since all previously known mummies produced by *Aleiodes* species had a posterior hole cut for emergence (Zaldívar–Riverón et al. 2008).

#### Discussion.

*Aleiodes frosti* sp. n. belongs to the *seriatus* species-group. This species resembles *Aleiodes nigricosta* (Enderlein, 1920) because of its entirely yellowish to honey yellow body, black stemmaticum and brown antenna, but differs in the honey brown fore wing vein C+SC+R, black in *Aleiodes nigricosta*. *Aleiodes frosti* sp. n. also differs in the extension of median longitudinal carina on metasoma, which is incomplete on tergite 2, but extends to half of tergite 3 in *Aleiodes nigricosta*, and the exceptionally elongate metasoma. *Aleiodes frosti* sp. n. is also similar to *Aleiodes elleni* sp. n. by the nearly straight hind wing vein RS, enclosing a marginal cell gradually widening toward wing apex, but it can be readily distinguish by the interrupted occipital carina on vertex, compared to the complete occipital carina of *Aleiodes elleni* sp. n. The diameter of lateral ocelli, 3–4× longer than ocell–ocullar distance, is also a diagnostic character shared only with one Neotropical species, *Aleiodes nigribasis* (Enderlein, 1920); however, most of the already mentioned diagnostic features for *Aleiodes frosti* sp. n. (e. g. shape of metasoma and hind wing vein RS, and color patern) are also useful to distinguish it from *Aleiodes nigribasis*. Within the Yanayacu species in the *Aleiodes seriatus* group it is similar to *Aleiodes greeneyi* because of the incomplete occipital carina at vertex. It differs from *Aleiodes greeneyi* by the entire yellowish body and its unusual long and narrow metasoma.

#### Etymology.

The species is named after the American poet Robert Frost (1874 – 1963), author of the poem “The Road Not Taken.” This species name is also a reference to that poem, and to the unusual emergence mode of this species, recorded here for the first time. The following quotation extracted from this poem summarizes its idea: “*Two roads diverged in a wood, and I – I took the one less traveled by*,” Robert Frost, 1920. This *Aleiodes* species takes a “road not taken” by other species, to its adulthood, by emerging in a different and unique way.

### Aleiodes greeneyi

Townsend, 2009

http://species-id.net/wiki/Aleiodes_greeneyi

#### Diagnosis.

Body length 4.9 mm; antenna with 42 segments; head color honey brown with black ocellar triangle; malar space 1.25× basal width of mandible; ocell–ocular distance about as long as width of lateral ocellus; occipital carina interrupted at vertex; mesopleuron granulate; apex of hind tibia with comb of flattened setae; propodeum strongly punctate with several longitudinally oriented rugae, longitudinal carina complete; metasomal terga 1–2 dark brown, terga 3–4 brown medially and dark brown laterally, reminder terga light brown; ovipositor sheaths about 0.6× hind basitarsus length.

#### Additional characters.

Last flagellomere with “bottle-nipple”-like tip; mesoscutum with carina at posterior margin complete although not well defined; scutellar sulcus with complete median carina plus one pair of slightly weaker lateral carina; fore wing vein 1M moderately curved at base; hind wing vein 2-1A indicated as a very short stub, vein m-cu present, interstitial to vein r-m; ovipositor sheaths about as long as hind tarsomere II, 0.6× hind basitarsus.

#### Type material examined.

(UWIM)

#### Biology.

*Aleiodes greeneyi* has been reared from a Geometridae caterpillar feeding on *Evodianthus funifer* (Cyclanthaceae).

#### Distribution.

Known only from the type locality, YBS, Napo province, ECUADOR.

#### Discussion.

*Aleiodes greeneyi* is known only by the holotype. It belongs to the *seriatus* species-group. The incomplete occipital carina at vertex and the moderate ocelli size are similar to those presented in *Aleiodes longikeros* sp. n. and *Aleiodes dyeri* sp. n.; however, the wing vein pattern is somewhat intermediate between these two species. *Aleiodes greeneyi* differs from *Aleiodes longikeros* sp. n. and *Aleiodes dyeri* sp. n. in having the pronotum and mesoscutum mostly black (yellow in *Aleiodes longikeros* sp. n. and *Aleiodes dyeri* sp. n.), and the hind coxa light brown (bicolored black and white in *Aleiodes longikeros* sp. n. and *Aleiodes dyeri* sp. n.). *Aleiodes greeneyi* was reared from geometrid larva. Within the *seriatus*-group species from Ecuador, *Aleiodes nebulosus* and *Aleiodes longikeros* sp. n. were also reared from Geometridae hosts.

### Aleiodes kingmani sp. n.

http://zoobank.org/4A47BA21-4AE9-4074-8F1A-B67F5EEB8EE2

http://species-id.net/wiki/Aleiodes_kingmani

Figures 52 , 53

#### Description of holotype.

Male. Body length 4.7 mm; antenna length 5.5 mm; fore wing length 4.3 mm.

Color. Mostly black. Head honey yellow, ocellar triangle black; antenna dark brown except apical border of pedicel honey yellow. Mesosoma almost entirely black; propleuron and ventral quarter of pronotum, mesoscutum mid-posteriorly and scutellum medially yellowish to honey yellow; posterior border of propodeum white. Fore and mid legs with whitish coxa, darkening apically to honey yellow apical femur and tibia, and brown tarsi; hind leg coxa, trochanter and trochantellus black, but apical border of trochanter and trochantellus, and a small ventral spot on trochantellus white; femur black on basal 2/5 and dorso-apically, otherwise yellowish; tibia and tarsi brown, subbasal whitish small band on tibia. Metasoma black dorsally except for the white T1; apical borders of T4–T7 whitish; basal T5–T7 brownish. Wings moderately infuscate; veins dark brown.

Head. 40 antennomeres, flagellomeres roughly 2.0× as long as wide, apical flagellomere with short pointed apex; malar space moderate, length 1.3× basal width of mandible, and approximately 1/3 eye height; in dorsal view eye 2.6× temples; occipital carina incomplete dorsally, curving toward lateral ocelli, well defined laterally but not meeting hypostomal carina; oral space small and circular, maximum width equal to basal width of mandible; clypeus slightly swollen; ocellus moderate, ocell–ocular distance short, about half diameter of lateral ocellus; maxillary palp not swollen; head surface sculpturing shining granulate, occiput smooth and shining; frons excavated with short lateral ridges.

Mesosoma. Sculpturing finely granulate; pronotum with some wrinkles laterally; mesopleuron coriaceous on central elevated area and some irregular latero-ventral parts; propodeum with mid-longitudinal carina incomplete, granulate postero-laterally with irregular wrinkles, and triangular rugose area mid-anteriorly with diverging wrinkles; notauli well defined anteriorly, narrow and crenulate, meeting a depressed rugose area posteriorly; posterior margin of mesoscutum bordered by complete carina; scutellar sulcus shallow, with median carina plus two pairs of poorly defined lateral carina.

Wings. Fore wing: stigma 4× longer than high; vein r 0.8× vein 2RS, 0.9× vein RS+Mb, and 0.7× as long as vein m-cu; vein 3RSa about 0.4 times vein 3RSb, and 0.8× vein 2M; vein 1CUa 2.7× vein 1cu-a; vein 1CUb slightly shorter than vein 1CUa; vein 1M moderately curved at basal portion. Hind wing: m-cu absent; vein M+CU 1.3× vein 1M; vein 1M 1.8× vein r-m; RS smoothly diverging from margin beyond middle; vein M dark brown, well pigmented; vein 2-1A absent.

Legs. Hind tibia without comb of modified setae; tarsal claw simple, with a comb of thin bristles medially; hind tibial spurs relatively long, hind basitarsus 2.4× longer than inner apical spur.

Metasoma. T1, T2 and basal 3/4 of T3 rugose costate, longitudinal carina present along this sculpturing; remainder T3 and T4 granular; remainder visible terga weakly shining coriaceous; T1 about as long as its apical width.

Female unknown.

Mummy. Length 9.5 mm, abdomen dark reddish brown, thorax, head and anal prolegs light brown, mummy bent (curled) ventrally, “J-shaped”, thorax inflated, exit hole barely round, located postero-dorsally at apex of mummy, posterior to hind abdominal prolegs.

#### Type material.

Type-locality: ECUADOR, Napo Province, Yanayacu Biological Station, YY-57074, S00°35.9', W77°53.4', 2163 m, cloud forest, June 20, 2011.

Type-specimen: Holotype male and mummy, point mounted separately. Top label: “ECUADOR: Napo Province / Yanayacu Biological Station / S00°35.9', W77°53.4', 2163m / CAPEA - NSF-BSI-07-17458 / (hand written) May 2011 / YY-57074; back (hand written): “em. 20-June-2011”. (UWIM)

#### Biology.

Reared from mummified Geometridae caterpillar on *Chusquea scandens*. The host was collected as mummy.

#### Discussion.

This species belongs to *circumscriptus*/*gastritor* species-group. It produces an unusual curled mummy, “J-shaped”, and emerge through a large and almost round exit hole at the posterior end. The white first tergite of *Aleiodes kingmani* sp. n., contrasting to the mostly black metasoma, is a useful diagnostic character shared only with *Aleiodes speciosus* and *Aleiodes townsendi* sp. n. *Aleiodes kingmani* sp. n. differs from *Aleiodes townsendi* sp. n. by the mostly black mesosoma (mostly yellowish in *Aleiodes townsendi* sp. n.), and the entirely black hind coxa (bicolored black and white in *Aleiodes townsendi* sp. n.). *Aleiodes kingmani* sp. n. differs from *Aleiodes speciosus* in having mesopleuron black (mostly yellow in *Aleiodes speciosus*) and entirely granulate (mostly smooth in *Aleiodes speciosus*); black region on head restricted to ocellar triangle (covering most of vertex and occiput dorsally in *Aleiodes speciosus*); hind coxa, trochanter and trochantellus black (yellow in *Aleiodes speciosus*); and occipital carina not meeting the hypostomal carina.

#### Etymology.

This species is named after Eduardo Kingman (Loja, February 23, 1913 – Quito, November 27, 1997), one of the greatest Ecuadorian artists, who dedicated his art to portray the indigenous people of Ecuador.

**Figures 52, 53.**
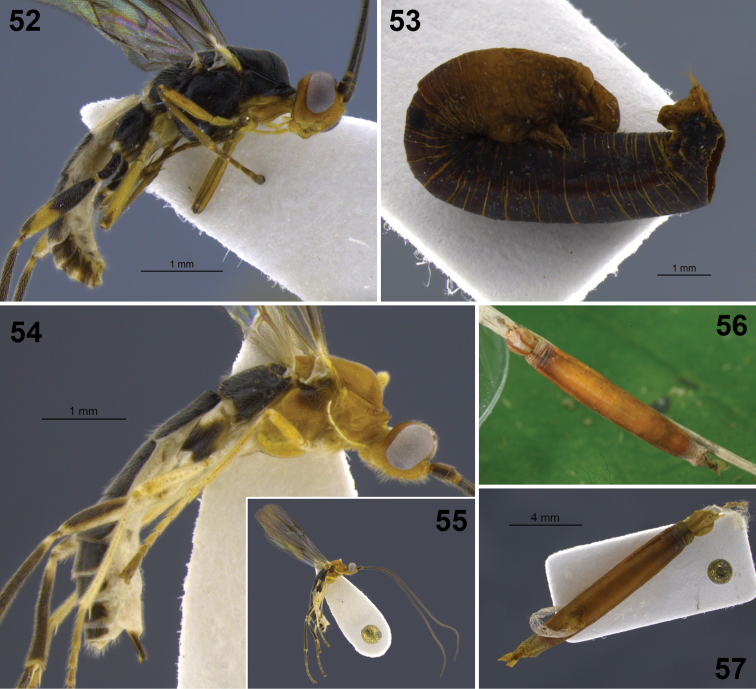
*Aleiodes kingmani* sp. n. **52** habitus **53** host mummy after parasitoid emergence **54–57** *Aleiodes longikeros* sp. n. **54** habitus **55** whole specimen habitus **56** host mummy before parasitoid emergence **57** host mummy after parasitoid emergence.

### Aleiodes longikeros sp. n.

http://zoobank.org/28CEB0BB-417E-4E91-A5FF-D232646AEEB9

http://species-id.net/wiki/Aleiodes_longikeros

Figures 54–57

#### Description of holotype.

Female (holotype). Body length 7.3 mm; antenna length 12.0 mm; fore wing length 7.0 mm.

Color. Head honey yellow, ocellar triangle brown; antenna dark brown, scape and pedicel honey brown with darker outer side. Mesosoma honey yellow, dorso-anterior corner of mesopeluron infuscate; metapeluron and propodeum black, posterior 1/5 of metapleuron white. Fore and mid leg whitish, tarsi darkening gradually, 5^th^ tarsomeres brown. Hind leg: coxa mostly dark brown, basal third white; trochanter and trochantellus white; femur white with dorso-subapical dark brown stain; tibia and tarsi dark brown except for sub-basal white band on tibia which is larger ventrally; tibial spurs white. Metasoma black dorsally, with white spots mid-apically on T1 and medially from T2 to T4; ventrally white. Wings weakly hyaline; veins dark brown.

Head. Antenna with 60 antennomeres, about 1.6× as long as body, flagellomeres narrower than in other species, most flagellomeres 2.5× longer than wide, apical flagellomere with long “bottle-nipple”-shaped apex; malar space 1.1× longer than basal width of mandible, 0.4× longer than eye height; in dorsal view eyes 3× longer than temples; occipital carina barely incomplete, almost meeting dorsally and directed toward vertex, well defined laterally and meeting hypostomal carina; oral space small and circular, maximum width 0.8× basal width of mandible; clypeus not swollen; ocell-ocular distance about as long as diameter of lateral ocellus; maxillary palp not swollen; head surface sculpturing shining granulate, occiput smooth and shining; higher face with a small longitudinal ridge and transverse rugosity directed to it; frons only shallowly excavated.

Mesosoma. Sculpturing shining granulate; pronotum smooth laterally, dorsally granulate and foveate; mesopleuron with central elevated area smooth, antero-dorsal corner rugose; propodeum coarsely shining granular with complete mid-longitudinal carina; notauli very shallow anteriorly, virtually absent. Mesoscutum with some transverse wrinkles on anterior region of notauli, mid-posterior depressed area with few longitudinal wrinkles; posterior margin of mesoscutum with complete carina; scutellar sulcus with median carina plus two pairs of complete lateral carina.

Wings. Fore wing: stigma 5.7× longer than high; vein r as long as vein 2RS, slightly longer than vein RS+Mb, and 0.75× vein m-cu; vein 3RSa 0.55× vein 3RSb, and as long as vein 2M; vein 1CUa 1.8× vein 1cu-a; vein 1CUb 2× vein 1CUa; vein 1M strongly curved in its basal portion. Hind wing: m-cu absent; vein M+CU 0.8× vein 1M; 1M about 2.4× longer than r-m; vein RS smoothly curved at middle; vein M straight dark brown, well pigmented; vein 2-1A present.

Legs. Hind tibia with comb of modified setae at apex; tarsal claw simple, not pectinate, with a comb of relatively long thin setae basally. Hind tibial spurs relatively short, hind basitarsus 3× longer than inner spur.

Metasoma. T1, T2 and basal 2/5 of T3 granulose striate, longitudinal carina present along this sculpturing; remainder visible terga granular coriaceous; ovipositor sheaths about 0.85× hind tarsomere II; apex of ovipositor sheaths truncate; T1 length 1.4× its apical width, strongly widening posteriorly, its apical width about 2× basal width.

Male unknown.

Mummy. Length 12.5 mm, light reddish brown, head and prolegs light yellow, head with two longitudinal brownish stripes, thorax compact and wrinkled, posterior apex withered, glue hole located ventrally on the thorax, exit hole irregular, located postero-dorsally, posterior to hind abdominal prolegs.

#### Type material.

Type-locality: ECUADOR, Napo Province, Yanayacu Biological Station, YY-57074, S00°35.9', W77°53.4', 2163 m, cloud forest, April 12, 2010.

Type-specimen: Holotype female and mummy, point mounted separately. Top label: “ECUADOR: Napo Province / Yanayacu Biological Station / S00°35.9', W77°53.4', 2163m / CAPEA - NSF-BSI-07-17458 / (hand written) March 2010 / 46444”; back (hand written): “em. 12 April 2010”. (UWIM)

#### Biology.

Reared from mummified Geometridae caterpillar (YY-46444), on *Chusquea scandens* (Poaceae). The host was collected as mummy.

#### Discussion.

*Aleiodes longikeros* sp. n. belongs to the *seriatus* species-group. This species has the longest antenna of any of these 24 newly described species, even though this is a medium sized species. Its antenna is about 1.6× longer than body, as compared with at most 1.3× in other species. The fore wing vein 1M strongly curved basally is also seen in *Aleiodes townsendi* sp. n. and *Aleiodes shakirae* sp. n., both belonging to *circumscriptus*/*gastritor* species-group, therefore both distinct from *Aleiodes longikeros* sp. n. by the absence of apical comb of flattened setae on hind tibia. *Aleiodes longikeros* sp. n. is similar to *Aleiodes dyeri* sp. n. (distinguishing features are discussed on diagnosis section on *Aleiodes dyeri* sp. n.). It also resembles *Aleiodes greeneyi* because of the dorsally incomplete occipital carina, from which it can be distinguished by the honey yellow mesosoma (dorsally black in *Aleiodes greeneyi*), and white mid-apical spots on metasomal terga 1–4 (mostly black to dark brown in *Aleiodes greeneyi*); mesopleuron smooth on dorsal elevated area (granulate in *Aleiodes greeneyi*). *Aleiodes longikeros* sp. n. can be distinguished from all New World species by the following combination of characters: fore wing second submarginal cell long and narrow, vein 2RS 0.35× longer than vein 2M; the long antenna; and strongly curved vein 1M on fore wing. In the key to Nearctic species of the *Aleiodes seriatus* group (Marsh and Shaw 1998), *Aleiodes longikeros* sp. n. runs to *Aleiodes preclarus*. The new species differs from *Aleiodes preclarus* by the above mentioned character combination, and also by the entirely yellowish head and mesosoma, with several dark spots in *Aleiodes preclarus*, and the smooth frons, as compared with porcate frontal sculpturing in *Aleiodes preclarus*.

#### Etymology.

From the Latin, meaning “long horned,” being a reference to the unusually long antenna of this species.

### Aleiodes luteosicarius sp. n.

http://zoobank.org/E5AD2981-1E53-4CBD-9BFF-DFB446513ECD

http://species-id.net/wiki/Aleiodes_luteosicarius

Figures 58–60 , 61–68 , 119 , 124

#### Description of holotype.

Female (holotype). Body length 6.9 mm; antenna length 7.6 mm; fore wing length 6.0 mm.

Color. Entire body honey brown to bronze, notum slightly darker; ocellar triangle black; antenna dark brown; wings hyaline; veins brown except C+SC+R, parastigma centrally, stigma, and R1 yellow; ovipositor mostly with same color of body, only weakly darkening apically.

Head. Antenna with 48 antennomeres, flagellomeres roughly 2.0× as long as wide, apical flagellomere with “bottle-nipple”-shaped apex; malar space as long as basal width of mandible, and 0.3× eye height; in dorsal view eye height 2.7× temple; occipital carina incomplete dorsally but not curved toward vertex, otherwise complete but not touching hypostomal carina; oral space small and circular, maximum width equal to basal width of mandible; clypeus not swollen; ocell–ocular distance 0.9× diameter of lateral ocellus; maxillary palp not swollen. Head surface sculpturing finely granulate, higher face with small longitudinal ridge and transverse rugosity directed to it, vertex coarsely granulate with some transverse wrinkles, occiput smooth and shining.

Mesosoma. Sculpturing mostly granulate; pronotum foveate laterally; mesopleuron rugose on anterior corner; propodeum mostly coarsely granulate with few longitudinal wrinkles posteriorly, laterally weakly rugose, with mid-longitudinal carina present on anterior 2/3; notauli present anteriorly, wide and shallow, posteriorly disappears in a depressed longitudinally rugose–striate area; posterior margin of mesoscutum with short carina just in front of scutellar sulcus; scutellar sulcus with strong median carina plus two pairs of strong but incomplete lateral carina.

Wings. Fore wing: stigma about 4× longer than high; vein r 0.7× vein 2RS, about as long as vein RS+Mb, and 0.5× as long as vein m-cu; vein 3RSa about 0.5× vein 3RSb, and as long as vein 2M; vein 1CUa 2.5× vein 1cu-a; vein 1CUb 1.8× vein 1CUa; vein 1M virtually straight, only very slightly curved at basal half. Hind wing: m-cu indicated as short pigmented vein just postfurcal to vein r-m; vein M+CU about 1.3× 1M; vein r-m as long as vein 1M; vein RS faint, smoothly curved at middle; vein M straight; vein 2-1A present.

Legs. Hind tibia without comb of modified setae; tarsal claw pectinate with distinct gap between apical claw and bristles; hind basitarsus 3× longer than inner apical spur on hind tibia; few rugositie dorso-laterally on outer side of hind coxa.

Metasoma. T1–T2 and basal half of T3 striated; remainder terga coriaceous; mid longitudinal carina complete from T1 throughout T2; ovipositor sheaths slightly shorter than hind tarsomere II, relatively thick and somewhat rounded at tip, uniformly and densely covered with relative short regular sized pubescence, but dorso-basally bare, setae length no longer than width of sheaths; T1 slightly longer than apical width.

Paratype variation. Body length 6.7–7.4 mm; antenna 48–50 segments; 3 to 5 carina on scutellar sulcus; about half of paratypes have a lighter body color, otherwise very similar to holotype.

Male. Body length 5.7 mm; 45 antennomeres; body color a little darker; ocelli larger, ocell–ocular distance 0.6× diameter of lateral ocellus; hind wing vein 1M relatively shorter, and vein m-cu just postfurcal to r-m.

Mummy. All mummies densely setose, setae mostly yellowish to light brown contrasting with dark brown body, head varying from dark brown to yellowish brown; morphology of mummies variable according to different host species, exit hole irregular, postero-dorsal.

#### Type material.

Type-locality: ECUADOR, Napo Province, Yanayacu Biological Station, YY-47385, S00°35.9', W77°53.4', 2163 m, cloud forest, May 26, 2010.

Type-specimen: Holotype female and mummy, point mounted separately. Top label: “ECUADOR: Napo Province / Yanayacu Biological Station / S00°35.9', W77°53.4’ 2163m / CAPEA - NSF-BSI-07-17458 / (hand written) Apr. 2010 / YY-47385”; back (hand written): “26-May-2010”. (UWIM)

Paratypes, 9 females and 1 male (UWIM). 8♀ and 1♂, same data as holotype, except: 1♀, October 27, 2009, reared from *Amastus* nr. *hyalina* (Erebidae), YY-42218, feeding on *Chusquea scandens*; 3 ♀ reared from *Pelochyta gandolfii* (Erebidae) feeding on *Chusquea scandens*: April 27, 2009, YY-37911, March 16, 2007, YY-20534, and March 9, 2011, YY-54645; 1♀, December 2, 2009, YY-43108, reared from *Desmotricha imitata* (Erebidae) on *Barnadesia parviflora* (Asteraceae); 1♀ April 25, 2013, YY-73553, reared from *Pelochyta gandolfii*, on unknown host plant; 1♀ September 24, 2013, YY-78795, reared from Erebidae; 1♀ October 3, 2013, reared from *Amastus* nr. *hyalina*; 1♂, May 21, 2011, hand collected at daylight by aspirator (Ridge trail), H. Aguirre col. 1♀, ECUADOR, Napo Province, Cuyuja, Chalpi Grande, 2800m, March 3, 2006, hand collected at light sheet – Yanayacu exp. 2006, D.L. Wagner col.

#### Biology.

Parasitoids on several Arctiinae (Erebidae) species: *Amastus coccinator* Schaus (including the holotype), *Pelochyta gandolfii* Schaus and *Amastus* nr. *hyalina* Dognin, collected on *Chusquea scandens* (Poaceae), and *Desmotricha imitata* Druce feeding on *Barnadesia parviflora* (Asteraceae). Nevertheless, *Amastus coccinator* and *Pelochyta gandolfii* have no previous rearing records feeding on *Chusquea scandens*, which is the most common plant in the sampling sites. Since Arctiinae caterpillars are highly active, it is possible that they were just wandering over this plant; therefore, feeding on *Chusquea scandens* for the these two species needs confirmation. Time span from host mummification to adult emergence was about one month.

#### Discussion.

*Aleiodes luteosicarius* sp. n. is the only species herein described which belongs to *pallidator* species-group. It is very similar to *Aleiodes falloni* sp. n., despite *Aleiodes falloni* sp. n. belongs to *circumscriptus*/*gastritor* species-group. *Aleiodes luteosicarius* sp. n. differs from *Aleiodes falloni* sp. n. in the following characters: ocell–ocular distance 0.3× diameter of lateral ocellus (about 0.9× in *Aleiodes falloni* sp. n.); at least half of tergite 3 striated, as in tergite 2 (tergite 3 mostly smooth–coriaceous); m-cu of hind wing slightly postfurcal to almost interstitial (antefurcal in females of *Aleiodes falloni* sp. n.); clypeus not swollen (swollen in *Aleiodes falloni* sp. n.); ovipositor sheaths relatively thick and somewhat rounded at tip, uniformly and densely covered with relative short regular sized pubescence, but dorso-basally bare, setae length no longer than width of sheaths (ovipositor sheaths sharpening at apex, setae concentrated apically, and not regular sized, longest setae about 1.5× longer than width of sheaths in *Aleiodes falloni* sp. n.); fore wing as long as body length (distinctly shorter in *Aleiodes falloni* sp. n.); stigma narrower (4× longer than high in *Aleiodes luteosicarius* sp. n. vs. 3× in *Aleiodes falloni* sp. n.); hind wing vein r-m as long as vein 1M (distinctly shorter in *Aleiodes falloni* sp. n.); depressed area on mesoscutum longitudinally rugose–striate (striations running antero-laterally from mid-posterior region); propodeum mostly coarsely granulate with few longitudinal wrinkles posteriorly, laterally weakly rugose (distinct pattern of rugosity on propodeum in *Aleiodes falloni* sp. n.); occipital carina strong and abruptly interrupted dorsally (weaker and gradually disappearing in *Aleiodes falloni* sp. n.); vertex coarsely granulate with some transverse wrinkles (finely granulate in *Aleiodes falloni* sp. n.). In the key to species of *pallidator* species-group from North America (S. Shaw et al. 2013) it runs to *Aleiodes pallidator* (Thunberg, 1822). The new species closely resembles *Aleiodes pallidator*, differing from it by the following: propodeum surface is entire rugose (laterally coriaceous in *Aleiodes pallidator*) and the longitudinal carina is present on anterior 3/4 (complete in *Aleiodes pallidator*); vein 1CUa about 2× length of 1cu-a (just slightly longer in *Aleiodes pallidator*); basal cell of hind wing very broad, vein r-m as long as vein 1M (narrower in *Aleiodes pallidator*); metasomal tergite 1 granular–rugose and tergite 2 striate–rugose (costate in *Aleiodes pallidator*); parasitoid on Arctiinae (Erebidae) (*Aleiodes pallidator* attacks Lymantriidae).

#### Comments.

All previous known species of the *pallidator* species-group are parasitoids on Lymantriinae caterpillars (S. Shaw et al. 2013, as Lymantriidae). This is the first record of a species in this group attacking Arctiinae, and also the first species of the group with known host from Neotropical region. The status of subfamily for these groups is relatively recent, and both belong to Erebidae. Species within the *pallidator* group has been consistently reared from the setose mummified caterpillars of Lymantriinae in Japan, Europe and North America. The host associations reported here broaden the known host range for this species-group, but also denotes its ecological preferences for attacking densely setose caterpillars.

#### Etymology.

From the Latin meaning “yellow killer,” referring to the main color of this parasitoid.

**Figures 58–60.**
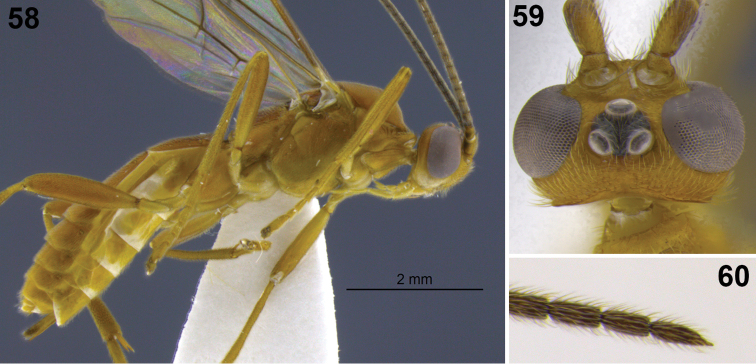
*Aleiodes luteosicarius* sp. n. **58** habitus **59** head, dorsal **60** apical flagellomeres.

**Figures 61–68.**
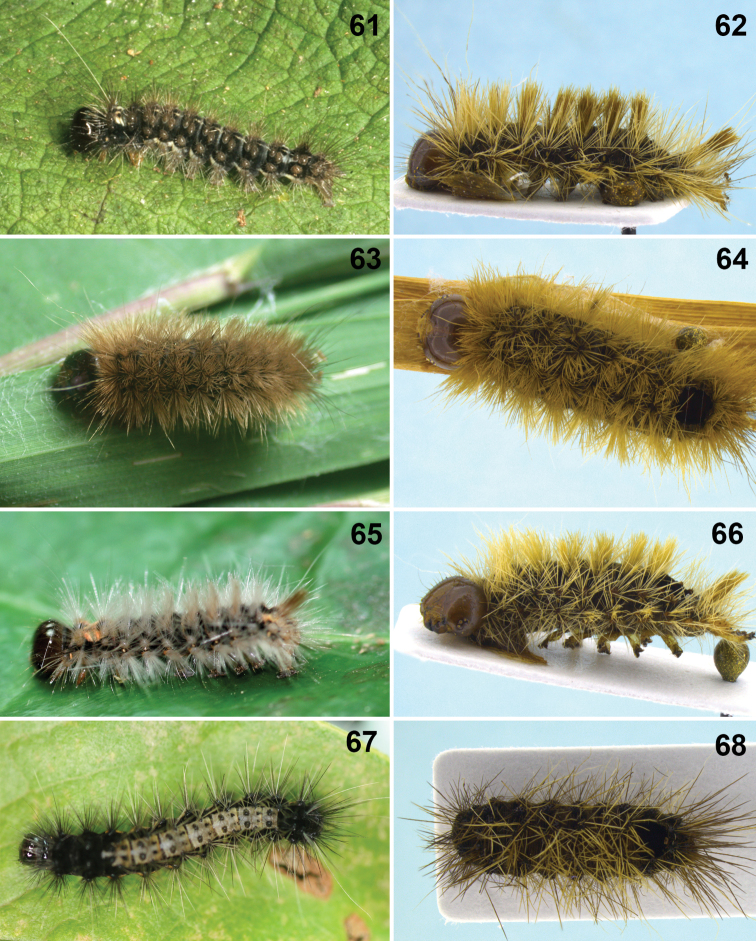
Arctiinae hosts of *Aleiodes luteosicarius* sp. n. **61** *Amastus coccinator* Schaus larva **62** host mummy (*Amastus coccinator*) after parasitoid emergence **63** *Pelochitas gandolfii* Schaus larva **64** host mummy (*P. galdolfi*) after parasitoid emergence **65** *Amastus* nr. *hyalina* Dognin larva **66** host mummy (*Amastus* nr. *hyalina*) after parasitoid emergence **67** *Desmotricha imitata* Druce larva **68** host mummy (*Desmotricha imitata*) after parasitoid emergence.

### Aleiodes marilynae sp. n.

http://zoobank.org/6AB9A206-B015-42CC-9632-31E33DFF6DCD

http://species-id.net/wiki/Aleiodes_marilynae

Figures 69–73

#### Description of holotype.

Female (holotype). Body length 6.6 mm; antenna length 7.5 mm; fore wing length 6.6 mm.

Color. Head whitish, except for the ocellar triangle black, and the light yellow face and palp. Mesosoma black but pronotum and propleuron light yellow, and small posterior spot on metapleuron and posterior 1/5 of propodeum whitish; fore legs coxa, trochanter, trochantellus and mostly femur yellow; fore femur with dorso-apical brown stain, reminder fore legs brown; mid legs dark brown, but trochanter and trochantellus white; hind legs black. Metasomal tergite 1 with a black oval spot medially spanning from just behind basal carina to posterior edges of the tergite, remainder of tergite 1 white; tergite 2 black medially, dark region almost quadrate in shape with lateral borders convex at 2/3 posterior, white laterally; tergite 3 mostly white, with basal almost semi-circular black mark; reminder terga white apically, basally black, more or less concealed by the preceding tergite; metasoma ventrally white with a pair of latero-basal spots on each sternite, the spots larger on the second sternite; ovipositor sheaths black, ovipositor yellow.

Head. 50 antennomeres; most flagellomeres roughly 2× longer than wide, apical flagellomere with small pointed apex; malar space wide, about 2.5× times basal width of mandible and almost as long as eye height; temple wide, in dorsal view about as long as eye; occipital carina absent; oral space small and circular, diameter about equal to basal width of mandible; clypeus weakly swollen; ocelli very small, ocell–ocular distance 2.2× diameter of lateral ocellus; head polished (smooth and shining).

Mesosoma. Sculpturing polished (smooth and shining); pronotum shining granular dorsally, otherwise shining coriaceous; mesopleuron sparsely punctuate; notauli very weakly indicated only anteriorly; posterior margin of mesoscutum without carina, smoothly depressing into scutellar sulcus; scutellar sulcus with five incomplete carina posteriorly; mesopleuron central region bare; epicnemial carina absent; longitudinal carina on propodeum absent.

Wings. Fore wing: stigma 3.8× longer than high; vein r 0.75× length of 2RS, 0.75× length of m-cu, and as long as vein RS+Mb; vein 3RSa 0.44× vein 3RSb, and as long as vein 2M; vein 1CUa about 2× vein 1cu-a; 1CUb 2× length of 1CUa; vein 1M almost straight. Hind wing: vein RS curved at middle, marginal cell narrowest point at middle; vein 1M about 2× longer than vein r-m; vein M+CU 0.9× vein 1M; vein m-cu absent; vein 2-1A present.

Legs. Tarsal claws simple, not pectinate; hind basitarsus 3× longer than inner apical spur of hind tibia; hind coxa smooth.

Metasoma. T1–T3 smooth and shining with punctuations on setal “pores”; reminder terga coriaceous; longitudinal carina barely indicated on T1 and absent on T2; petiole broad, 0.8× longer than its apical width; ovipositor sheaths about as long as hind tarsomere II.

Male unknown.

Mummy. Length 14.9 mm, mostly pale brown with some dark spots, dorsally at middle of abdomen with faint “X-shape” mark and four withered expansions on each tip of the mark, thorax very strongly compact and abdomen angled upward, very similar to *Aleiodes capillosus* mummies, glue hole ventrally on thorax, exit hole postero-dorsal, between abdominal and anal prolegs.

#### Type material.

Type-locality: ECUADOR, Napo Province, Yanayacu Biological Station, YY-44198, S00°35.9', W77°53.4', 2163 m, cloud forest, February 5, 2010.

Type-specimen: Holotype female and mummy, point mounted separately. Top label: “ECUADOR: Napo Province / Yanayacu Biological Station / S00°35.9', W77°53.4’ 2163m / CAPEA - NSF-BSI-07-17458 / (hand written) Dec. 2009 / 44198”; back (hand written): “em. 5 Feb 2010”. (UWIM)

#### Biology.

Host plant: *Acalypha macrostachya* (Euphorbiaceae). Host Lepidoptera: Geometridae – common name: “cachos X blanca en la espalda”. The parasitoid adult emerged one month after host mummification.

#### Discussion.

This is the fourth species of the *gressitti* species-group described. Of the former three, two are from New World, *Aleiodes lissos* Marsh & Shaw, 2003 and *Aleiodes capillosus* Townsend, and one from Campbell Island in the South Pacific: *Aleiodes gressitti* Muesebeck, 1964. This new species most resembles the Neotropical *Aleiodes capillosus* because of the absence of occipital and epicnemial carina, and also in the mostly black mesosoma and infuscate wings. It differs from *Aleiodes capillosus* in the whitish head, except for the ocellar triangle being black and the face light yellow, pronotum and propleuron, mid trochanter and trochantellus white, and fore leg coxa, trochanter, trochantellus and mostly femur yellow (while all these parts are black in *Aleiodes capillosus*). The malar space is very large, being about as long as the eye height (at most 1/2 in other *gressitti*-group species). The mummies produced by *Aleiodes marilynae* sp. n. and *Aleiodes capillosus* are very similar in the extremely contracted thorax and relatively swollen abdomen, and also in the characteristic acute angle at which the mummy is attached to the substrate.

#### Etymology.

This species is named in honor of Marilyn Rieden Shaw, wife of the co-author, Scott R. Shaw, in gratitude for her support for his entomological studies over many years.

**Figures 69–73.**
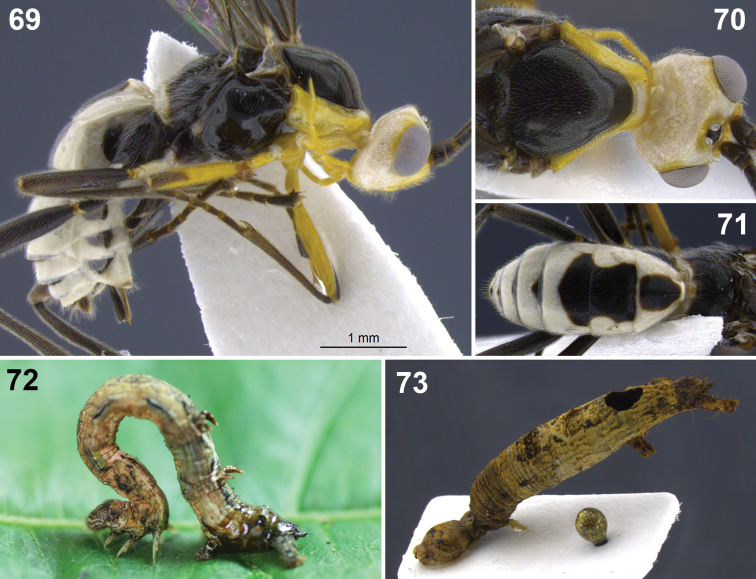
*Aleiodes marilynae* sp. n. **69** habitus **70** head and mesoscutum, dorsal **71** metasoma, dorsal **72** host larva (Geometridae) **73** host mummy after parasitoid emergence.

### Aleiodes mirandae sp. n.

http://zoobank.org/80CF0827-3BF3-473E-94D4-4E35D0200C8A

http://species-id.net/wiki/Aleiodes_mirandae

Figures 74–76

#### Description of holotype.

Female (holotype). Body length 5.4 mm; antenna length 6.3 mm; fore wing length 4.8 mm.

Color. Mostly black. Head orangish yellow, including mandibles and palp, but mandibles tip brown; ocellar triangle black; antenna dark brown except apical border of pedicel honey yellow. Mesosoma almost entirely black; propleuron, pronotum, and scutellum medially honey yellow; posterior border of propodeum white. Fore leg yellowish; outer apical 1/3 of femur infuscate; basal half of tibia and tarsi brown. Mid leg with same pattern of fore leg but ground color whitish. Hind leg black; light yellow markings on apical border of trochanter and trochantellus, small ventral spot on trochantellus, apical half of femur latero-ventrally, and small sub-basal band on tibia. Metasoma black dorsally, bordered white; T1, triangular mid-basal area on T2 and apical borders of T3–T7 white; ventrally white but infuscate medially; ovipositor sheaths basal 1/3 whitish, apical 2/3 black. Wings weakly infuscate; veins dark brown.

Head. Antenna with 46 antennomeres, flagellomeres roughly 2.0× as long as wide, apical flagellomere with very short pointed tip; malar space wide, about 1.8× basal width of mandible, and 0.6× eye height; in dorsal view eye 1.4× temple; occipital carina incomplete, close but not meeting dorsally and curving toward lateral ocelli, well defined laterally and meeting hypostomal carina; oral space small and circular, maximum width equal to basal width of mandible; clypeus slightly swollen; ocelli small, ocell–ocular distance about 1.8× diameter of lateral ocellus; maxillary palp not swollen; head surface sculpturing granulate, occiput smooth and shining; frons excavated with short lateral ridges.

Mesosoma. Sculpturing finely granulate; pronotum foveate; mesopleuron central disc mostly smooth and bare, posteriorly and ventrally coriaceous, antero-dorsal corner rugose; propodeum coarsely granular with complete mid-longitudinal carina; notauli well defined and crenulate anteriorly, barely defined but traceable posteriorly, meeting a depressed rugose area; posterior margin of mesoscutum bordered with complete carina; scutellar sulcus with median carina plus two pairs of incomplete lateral carina.

Wings. Fore wing: stigma about 4× longer than high; vein r 0.8× vein 2RS, as long as vein RS+Mb, and 0.75× as long as vein m-cu; vein 3RSa about 0.5× vein 3RSb, and 0.9× vein 2M; vein 1CUa 2.5× vein 1cu-a; vein 1CUb 2× vein 1CUa; vein 1M evenly slightly curved. Hind wing: m-cu absent; M+CU 0.8× 1M; vein 1M almost 3× vein r-m; vein RS smoothly curved at middle; vein M dark brown, well pigmented; vein 2-1A present, short.

Legs. Hind tibia without apical comb of modified setae; tarsal claw simple, not pectinate, with a comb of relatively long thin setae basally; hind tibial spurs relatively short, about 1/4 basitarsus length.

Metasoma. T1, T2 and basal 3/4 of T3 rugose costate, longitudinal carina present along this sculpturing; remainder T3 and T4 granular; remainder visible terga weakly shining coriaceous; ovipositor sheaths 0.7× length of hind basitarsus; T1 length slightly longer than its apical width.

Variation. 45–46 antennomeres; scutellar sulcus with 5 or 7 carina; white markings larger: almost all apical 1/2 on hind femur, basal 1/2 of hind tibia, basal spot on inner hind basitarsus, and throughout T2 length medially (widening toward base) reaching T3 basally as tiny central mark.

Male unknown.

Mummy. Length 10.0 mm, entire mottled pale yellow and gray, thorax compact and wrinkled, anal prolegs extended posteriorly, exit hole irregular, located postero-dorsally, posterior to hind abdominal prolegs.

#### Type material.

Type-locality: ECUADOR, Napo Province, Yanayacu Biological Station, S00°35.9', W77°53.4', 2163 m, cloud forest, 16 May 2011, M. Bryant col.

Type-specimen: Holotype female, point mounted. Top label: “ECUADOR: Napo Province / Yanayacu Biological Station / S00°35.9', W77°53.4’ 2163m / M. Bryant, 10–20 May 2011 / NSF-DEB-10-20751 / (hand written) blacklight / 16 May 2011”. (UWIM)

Paratypes, 3 females (UWIM): same locality as holotype, different collecting dates and methods: 1♀, March 15, 2010, reared, YY-45457; 1♀, February 7, 2010, reared YY-53818; 1♀, February 9, 2010, reared, YY-53961.

#### Biology.

Reared from a Geometridae species, common name “palito café chusquea”, feeding on *Chusquea scandens* (Poaceae). Morphology of mummies and rearing data corroborate a single host species. Three weeks elapsed between mummification until adult emergence.

#### Discussion.

*Aleiodes mirandae* sp. n. belongs to *circumscriptus*/*gastritor* species-group. It is similar to *Aleiodes napo* sp. n. in the very small ocelli (ocell–ocular distance about 2× diameter of lateral ocelli), and also the mostly smooth mesopleuron. It differs from *Aleiodes napo* sp. n. in the rugose depressed mid-posterior area on mesoscutum, flat and granular in *Aleiodes napo* sp. n., the head, except black ocellar triangle, pronotum, propleuron and scutellum orangish yellow, all black in *Aleiodes napo* sp. n. except for small reddish marking on temples. The color pattern is very similar to *Aleiodes kingmani* sp. n., but additionally to already mentioned diagnostic characters *Aleiodes mirandae* sp. n. have hind wing vein M+CU shorter than 1M, while in *Aleiodes kingmani* sp. n. 1M is more than 2× longer than r-m. The host species “palito café chusquea” (Geometridae) is the same species attacked by *Aleiodes nubicola* sp. n. and *Aleiodes shakirae* sp. n..

#### Etymology.

This species is named after Miranda Bryant, collector of the holotype.

**Figures 74–76.**
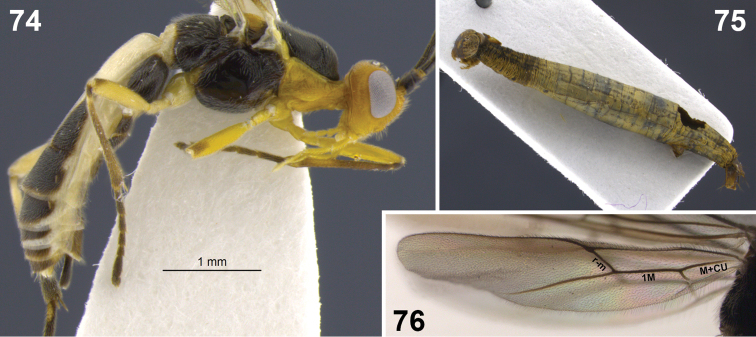
*Aleiodes mirandae* sp. n. **74** habitus **75** host mummy after parasitoid emergence **76** hind wing.

### Aleiodes napo sp. n.

http://zoobank.org/6779D464-D253-4A4D-B873-B955737CD320

http://species-id.net/wiki/Aleiodes_napo

Figures 77–81

#### Description of holotype.

Female. Body length 5.0 mm; antenna length 6.3 mm; fore wing length 5.3 mm.

Color. Mostly black. Head with a small brown stain on temples, just behind eyes; palp, fore and mid coxa, trochanter and trochantellus pale yellowish. Metasomal tergite 1 white, with a black oval spot medially spanning from just behind basal carina to posterior edges of the tergite; remainder terga mostly black, white laterally; metasoma ventrally white with a pair of latero-basal spots on each sternite, the spots larger on the second sternite; ovipositor sheaths black, ovipositor yellow.

Head. 47 antennomeres; most flagellomeres about 1.5× longer than wide, apical flagellomere with small pointed tip; malar space moderate, about 1.5× times basal width of mandible, and 0.5× eye height; temple wide, in dorsal view slightly shorter than eye; occipital carina absent dorsally, running toward vertex, ventrally almost meeting hypostomal carina; oral space small and circular, diameter about equal to basal width of mandible; clypeus with distinct line separating dorsal and ventral surfaces, dorsally coriaceous, ventrally convex and smooth; ocelli very small, ocell–ocular distance about 2.5× diameter of lateral ocellus; head, including frons, granulate.

Mesosoma. Mesoscutum and scutellum granular coriaceous; pronotum dorsally and laterally on anterior half granular with scrobiculate sulcus, reminder lateral pronotum smooth except for the posterior corner coarsely rugose; propleuron granular–coriaceous; mesopleuron mostly smooth centrally to finely coriaceous, antero-dorsal corner weakly rugose–punctate; metapleuron and propodeum coarsely granular; longitudinal carina on propodeum almost complete; notauli present only anteriorly, narrow and crenulate, mid-posterior area of mesoscutum not depressed and granulate; entire posterior margin of mesoscutum bordered by carina; scutellar sulcus with median carina plus two pairs of lateral carina.

Wings. Fore wing: stigma 4.4× longer than high; vein r 0.65× length of 2RS, 0.7× length of m-cu, and 0.85× vein RS+Mb; vein 3RSa 0.3× vein 3RSb, and 0.93× vein 2M; vein 1CUa 1.4× vein 1cu-a; 1CUb 2.8× length of 1CUa; vein 1M almost straight. Hind wing: vein RS curved at middle, marginal cell narrowest point at middle; vein 1M 2.2× longer than vein r-m; vein M+CU 0.68× vein 1M; vein m-cu absent; vein 2-1A present.

Legs. Apex of hind tibia without comb of modified setae; tarsal claws simple, not pectinate; hind basitarsus about 3× inner apical spur of hind tibia; hind coxa granulate basally.

Metasoma. T1 striate–rugose; T2 striated on basal 2/3; apical 1/3 of T2 and basal half of T3 coriaceous; longitudinal carina present along with the striated sculpturing on T1 and T2; T1 0.87× longer than its apical width; ovipositor sheaths about as long as hind tarsomere II.

Male unknown.

Mummy. Length 12.0 mm, head honey yellow, thorax pale brown, abdomen dark reddish brown, almost tubular in shape, exit hole located postero dorsally.

#### Type material.

Type-locality: ECUADOR, Napo Province, Yanayacu Biological Station, YY-48553, S00°35.9', W77°53.4', 2163 m, cloud forest, July 14, 2010.

Type-specimen: Holotype female and mummy, point mounted separately. Top label: “ECUADOR: Napo Province / Yanayacu Biological Station / S00°35.9', W77°53.4’ 2163m / CAPEA – NSF-BSI-07-17458 / REARED / 2010 (hand written) May 48553”; back (hand written): “14-Jul-2010”. (UWIM)

#### Biology.

Reared from a Noctuidae caterpillar (common name “raya roja a los lados chusquea”) collected on *Chusquea scandens* (Poaceae). The parasitoid took six weeks from host mummification until emergence.

#### Discussion.

This species belongs to *circumscriptus*/*gastritor* species-group. The color pattern of *Aleiodes napo* sp. n. is similar to those of *Aleiodes capillosus*; however, it does not belong to the *Aleiodes gressitti* species group because of the sculpturing on metasomal tergite 3, which is granular coriaceous on basal half. *Aleiodes napo* sp. n. differs from *Aleiodes capillosus* also in the presence of both occipital and epicnemial carina, though the former is incomplete dorsally, the presence of a complete longitudinal carina on metasomal terga 1 and 2, body sculpturing mostly granular–coriaceous, and longitudinal carina on propodeum almost complete (in *Aleiodes capillosus* all the above mentioned carina are absent, and the body sculpturing mostly smooth). Within the *circumscriptus*/*gastritor* group, *Aleiodes napo* sp. n. is similar to *Aleiodes mirandae* sp. n. in having a smooth central disc of mesopleuron and hind wing vein M+CU shorter than 1M, but differs from it in the almost entirely black head and thorax, mostly orangish yellow in *Aleiodes mirandae* sp. n., and the posterior central region of mesoscutum flat and granular, depressed and rugose in *Aleiodes mirandae* sp. n.

#### Etymology.

This species is named after the indigenous inhabitants of the eastern Ecuador, for whom the Province (locality of the type) is also named: the Napo Runas.

**Figures 77–81.**
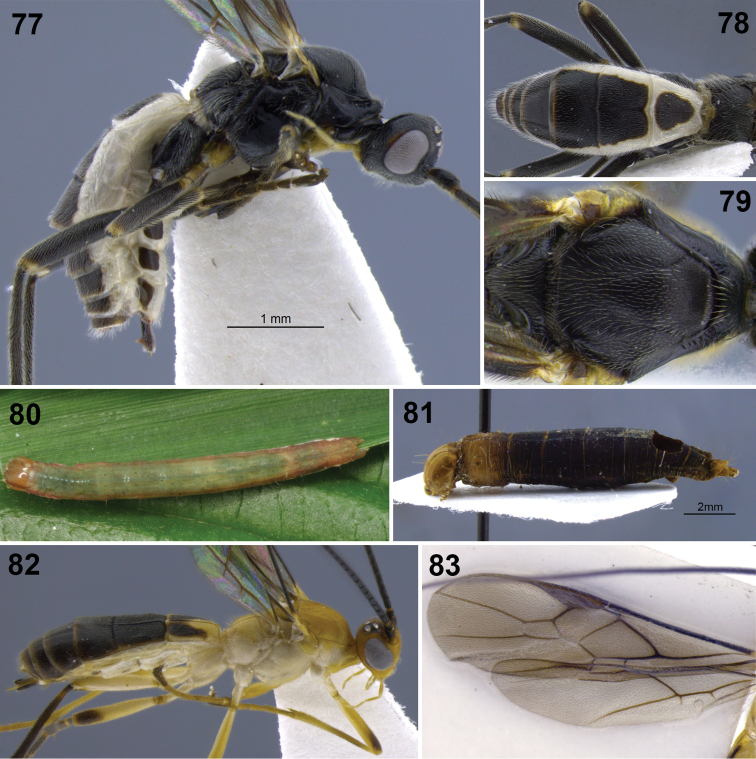
*Aleiodes napo* sp. n. **77** habitus **78** metasoma, dorsal **79** mesonotum, dorsal **80** host larva (Noctuidae) **81** host mummy after parasitoid emergence **82, 83** *Aleiodes nebulosus* Townsend **82** habitus **83** wings.

### Aleiodes nebulosus

Townsend, 2009

http://species-id.net/wiki/Aleiodes_nebulosus

Figures 82 , 83

#### Diagnosis.

Body length 6.1 mm; antenna with 47 segments; head color honey brown with black ocellar triangle; malar space 1.2× basal width of mandible; ocell–ocular distance 1.25× width of lateral ocellus; occipital carina complete; mesopleuron granulate; apex of hind tibia with comb of flattened setae; propodeum granulate anteriorly and rugose posteriorlly, longitudinal carina complete; metasomal terga mostly dark brown–black, tergite 1 with antero-median off-white marking; ovipositor sheaths length about half of hind basitarsus length.

#### Additional characters.

Last flagellomere with short pointed tip; mesoscutum with complete and well defined carina bordering posterior margin; scutellar sulcus with seven carina; fore wing vein 1M slightly curved at base; hind wing vein 2-1A absent, vein m-cu present, distinctly antefurcal to vein r-m; ovipositor sheaths about as long as hind tarsomere II, 0.5× hind basitarsus. Different than originally described, the sternaulus is absent.

Type material examined. (UWIM)

#### Biology.

*Aleiodes nebulosus* has been reared from unidentified Noctuidae caterpillar feeding on *Acalypha platyphylla* (Euphorbiaceae). One hyperparasitoid, *Mesochorus* sp. (Ichneumonidae), was reared from a similar mummy.

#### Distribution.

Known only from the type locality, Isla de Las Palmas, Napo province, ECUADOR, at 1,885 meters elevation.

#### Discussion.

*Aleiodes nebulosus* is known only by the holotype. In the *Aleiodes seriatus* species-group, *Aleiodes nebulosus* is similar to *Aleiodes elleni* sp. n. in having the occipital carina complete at vertex. These two species are also similar in color pattern because of the most yellowish head and mesoscutum, and the mostly dark brown to black metasoma with a whitish mark antero-medially. Distinguishing features between *Aleiodes nebulosus* and *Aleiodes elleni* sp. n. are presented in the discussion section for *Aleiodes elleni* sp. n.

### Aleiodes nubicola sp. n.

http://zoobank.org/198B4299-54FC-41C7-A661-CEE1AABFCB89

http://species-id.net/wiki/Aleiodes_nubicola

Figures 84–91

#### Description of holotype.

Female (holotype). Body length 3.8 mm; antenna length 4.9 mm; fore wing length 3.6 mm.

Color. Mostly dark brown to black. Head: gena and clypeus honey yellow; mandibles and palp whitish, but teeth brown. Mesosoma: lighter stripe, brown to reddish brown, laterally on mesopeluron; mesoscutum and scutellum mid apically yellowish; metapleuron just posteriorly whitish. Legs mostly yellowish but all tibia and tarsi brownish, hind coxa, trochanter and trochantellus dark brown to black, 1/3 basal of hind femur brown. Metasoma: ventrally, central apical T4 and remainder apical visible terga whitish to light yellow; ovipositor sheaths dark brown. Wings hyaline, veins dark brown, but some apical veins, stigma and base of fore wind M+CU and 1A light brown to almost colorless.

Head. Antenna with 38 antennomeres, flagellomeres roughly 2.0× as long as wide, apical flagellomere lanceolate, without pointed apex; malar space moderate, length 1.65× basal width of mandible, and about 0.65× eye height; in dorsal view sub-rectangular (temples not receding), eye height 2× temples; occipital carina complete and well defined, reaching hypostomal carina; oral space small and circular, maximum width about equal to basal width of mandible; clypeus not swollen; ocelli small, ocell–ocular distance 1.6× diameter of lateral ocellus; maxillary palp not swollen; head surface sculpturing finely granulate, but occiput smooth and shining.

Mesosoma. Sculpturing mostly granulate; pronotum with some wrinkles; mesopleuron anterior corner rugose; propodeum more coarsely granulate, with long mid-longitudinal carina on basal 2/3; notauli deep and crenulate anteriorly, porsteriorly meeting on depressed area; posterior margin of mesoscutum with short carina, just anterior to scutellar sulcus; scutellar sulcus with incomplete median carina, otherwise smooth.

Wings. Fore wing: stigma about 5× longer than high; vein r 0.65× vein 2RS, 0.8× vein RS+Mb, and 0.7× as long as vein m-cu; vein 3RSa about 0.3 times vein 3RSb, about 0.8× vein 2M; vein 1CUa about 2.5 times vein 1cu-a; vein 1CUb about 2.3 times 1CUa; vein 1M evenly slightly curved. Hind wing: m-cu indicated as short pigmented not tubular vein just postfurcal to vein r-m; vein M+CU as long as vein 1M; vein 1M 1.8× vein r-m; vein RS smoothly curved at middle; vein 2-1A present as a very short stub.

Legs. Hind tibia without apical comb of modified setae; tarsal claw simple, not pectinate; apical spurs on hind tibia small, hind basitarsus 4× longer than inner spur.

Metasoma. T1–T3 rugose–striated with granulate background; remainder terga granulate; mid longitudinal carina complete from T1 throughout; metasoma outline narrow, petiole relatively small, T1 1.2× longer than its apical width, ovipositor sheaths as long as hind tarsomere II.

Paratype variation. Body length 3.8–4.6 mm; antenna with 38–40 antennomeres; head in most specimens mostly dark brown, except most of gena and temples just behind eyes honey yellow, but the gena is entirely dark brown in two type specimens; the clypeus color is also variable, in most specimens it is contrasting honey yellow, but in two specimens the clypeus has the same color of face; scutellum color varies from entire black to yellow on apical half, scutellar sulcus varies from yellow to black; light lateral stripes on mesopleuron varies from brown, reddish brown to yellow, in one specimen the stripes are connected by ventral yellowish stripe; most paratypes with metasomal terga 4 and 5 mostly blackish; position of hind wing vein m-cu varies from just postfurcal to just antefurcal; hind basitarsus 4–5× longer than inner apical spur on hind tibia.

Male. Essentially as in female, but eyes slightly smaller; antenna with 35 and 38 segments; metasomal terga entire dark brown in on specimen, the other with whitish central markings throughout all metasomal terga from apical tergite 1.

Mummy. Length 8.5–10.8 mm, entire brown, mummy with elongate aspect and thin skin, widening gradually from neck to posterior exit hole, thorax wrinkled, mummy attached to the substrate by silk posteriorly at prolegs region, exit hole irregular, located postero-dorsally anterior to abdominal prolegs, but in two specimens the hole is located postero-ventrally.

#### Type material.

Type-locality: ECUADOR, Napo Province, Yanayacu Biological Station, YY-47035, S00°35.9', W77°53.4', 2163 m, cloud forest, May 7, 2010.

Type-specimen: Holotype female and mummy, point mounted. Top label: “ECUADOR: Napo Province / Yanayacu Biological Station / S00°35.9', W77°53.4’ 2163m / CAPEA - NSF-BSI-07-17458 / (hand written) em. 7 May 2010 / YY-47035”; back (hand written): “Abr-2010 / 7-May-2010”. (UWIM)

Paratypes, 5 females and 3 males (UWIM), same data as holotype, different dates: 1♀ March 17, 2010, YY-45382; 2♀ May 4, 2010, YY-47069 and YY-46993; 1♀, October 8, 2010, YY-50796; 1♀, November 12, 2010, YY-52470; 1♂ March 18, 2010, YY-45448; 1♂ April 21, 2010, YY-46669; 1♂ April 22, 2010, YY-46568. 1♀, ECUADOR, Napo, Baeza, 2000m, Feb. ‘79 Mason. (CNC).

#### Biology.

All specimens reared on the same Geometridae host caterpillar species (common name “palito café chusquea”) feeding on *Chusquea scandens* (Poaceae), collected from February to April. Consistent morphology of mummies and caterpillars support a single host species for this parasitoid. There is a variation in dorso-ventral orientation of the exit hole as observed in other species by M. Shaw (1983). All host caterpillars were collected during 2^nd^ (all males and one female) or 3^rd^ (only females) larval instars. Time span between host mummification and adult emergence varied mostly from two to three weeks, but one female took almost two months to emerge.

#### Discussion.

*Aleiodes nubicola* sp. n. is similar to *Aleiodes cacuangoi* sp. n. and *Aleiodes atripileatus* (see diagnosis of *Aleiodes cacuangoi* for differences). This species also resembles *Aleiodes arbitrium* in the size of ovipositor and the mostly dark brown head. It differs from *Aleiodes arbitrium* by the longer malar space, about 1.6× longer than mandible width at base (about 1.0× in *Aleiodes arbitrium*), the mostly black pronotum and mesonotum (mostly brownish orange in *Aleiodes arbitrium*), and the position of light marks on head bordering eyes on temples (same marks on vertex in *Aleiodes arbitrium*). The host species “palito café chusquea” (Geometridae) is the same species attacked by *Aleiodes mirandae* sp. n. and *Aleiodes shakirae* sp. n.

#### Etymology.

From Latin, means “cloud inhabiting”, a reference for the cloud forest habitat.

**Figures 84–91.**
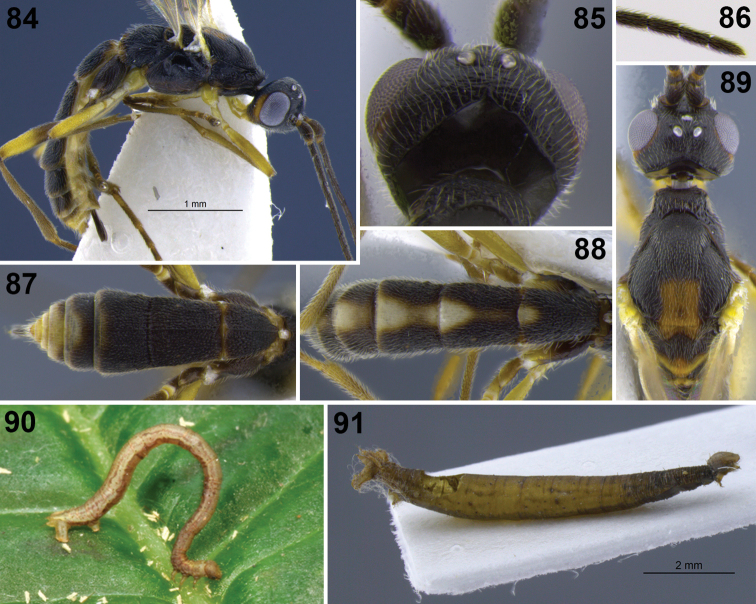
*Aleiodes nubicola* sp. n. **84** habitus **85** head occiput and vertex **86** apical flagellomeres **87** female holotype metasoma, dorsal **88** male paratype metasoma, dorsal **89** head and mesonotum, dorsal **90** host larva (Geometridae) **91** host mummy after parasitoid emergence.

### Aleiodes onyx sp. n.

http://zoobank.org/6BBAEA0E-F0BA-4CFF-B472-0FED7C7656DF

http://species-id.net/wiki/Aleiodes_onyx

Figures 92–95

#### Description of holotype.

Female (holotype). Body length 4.2 mm; antenna length 4.5 mm; fore wing length 4.1 mm.

Color. Mostly black. Head orangish except black ocellar triangle; cheeks, palp and mandibles whitish; mesocutum with postero-median yellowish square mark; scutellum lighter; fore coxa and femur honey yellow; mid coxa, and all trochanter and trochantellus whitish; most of mid and hind femur whitish; all femur with small brown spot apical–dorsally; all tibia and tarsi brown; hind coxa black; hind tibial spurs and base of basitarsi honey brown; tip of metasoma behind T5 brown; ovipositor sheaths dark brown on apical half, basal half whitish.

Head. Antenna with 33 antennomeres, flagellomeres roughly 2.0× as long as wide, apical flagellomere with small pointed tip; malar space moderate, length about 1.5× basal width of mandible, and half eye height; in dorsal view eye height 2× temples; occipital carina interrupted at vertex, ventrally reaching hypostomal carina; oral space small and circular, maximum width equal to basal width of mandible; clypeus not swollen; ocelli moderate, ocell–ocular distance as long as diameter of lateral ocellus; maxillary palp not swollen; head surface sculpturing finely granulate, but occiput smooth and shining.

Mesosoma. Sculpturing mostly granulate; pronotum with some wrinkles; mesopleuron with anterior corner rugose; propodeum more coarsely granulate, with long mid-longitudinal carina on basal 2/3; notauli deep and crenulate anteriorly, meeting on depressed area posteriorly; posterior margin of mesoscutum with short carina, just anterior to scutellar sulcus; scutellar sulcus with median carina plus two pairs of poorly defined lateral carina.

Wings. Fore wing: stigma 5.5× longer than high; vein r 0.75× vein 2RS, 1.25× vein RS+Mb, and 0.7× as long as vein m-cu; vein 3RSa about 0.3× vein 3RSb, and 0.8× vein 2M; vein 1CUa 2.4× vein 1cu-a; vein 1CUb 2× 1CUa; vein 1M evenly slightly curved. Hind wing: m-cu indicated as short pigmented not tubular vein just postfurcal to vein r-m; vein M+CU about as long as vein 1M; vein 1M 1.4× vein r-m; RS smoothly curved at middle; vein 2-1A present.

Legs. Hind tibia without comb of modified setae; tarsal claw simple, not pectinate, with a comb of relatively long thin setae basally; hind basitarsus 3× longer than inner apical spur on hind tibia.

Metasoma. T1–T3 rugose–striated with granulate background; remainder terga granulate; mid longitudinal carina complete from T1 throughout T3; T1 0.8× longer than its apical width; ovipositor sheaths as long as hind tarsomere II.

Paratype variation. None observed, virtually identical to holotype.

Male unknown.

Mummy. Length about 6.5 mm, body entire graphite metallic black color, head orangish yellow, mummy aspect robust, body with one row of setal sockets on each segment and sparse setae except dorsally, wrinkles on thorax; exit hole irregular, located postero-dorsally.

#### Type material.

Type-locality: ECUADOR, Napo Province, Yanayacu Biological Station, YY-57102, S00°35.9', W77°53.4', 2163 m, cloud forest, July 11, 2011.

Type-specimen: Holotype female and mummy, point mounted separately. Top label: “ECUADOR: Napo Province / Yanayacu Biological Station / S00°35.9', W77°53.4’ 2163m / CAPEA - NSF-BSI-07-17458 / (hand written) May 2011 / 57102”; back (hand written): “11-Ago-2011”. (UWIM)

Paratype, female: same data as holotype, except: July 8, 2011, YY-57101. (UWIM)

#### Biology.

All type specimens were reared from the same Zygaenidae caterpillar species (“espalda tomate rubiacea”) feeding on *Notopleura plagiantha* (Rubiaceae). Parasitoid emerged about five weeks after host mummification. Five caterpillars were collected together from the same plant, suggesting gregarious feeding behavior by the host caterpillars; four of them were parasitized, but adult parasitoids emerged from only two.

#### Discussion.

This species belongs to *circumscriptus*/*gastritor* species group. *Aleiodes onyx* sp. n. is similar to *Aleiodes atripileatus*; however, it can be distinguished by color patterns: head entirely orangish except ocellar triangle black (occiput mostly black in *Aleiodes atripileatus*), propleuron and ventral 1/4 pronotum honey brown (dark brown–black in *Aleiodes atripileatus*), mesopleuron whole black (ventral 1/2 honey brown in *Aleiodes atripileatus*), mesoscutum postero-central region honey yellow (whole dark-brown–black in *Aleiodes atripileatus*); as well as sculpturing features: mesopleuron central elevated area smooth (granulate in *Aleiodes atripileatus*), propodeum extensively rugose (granulate in *Aleiodes atripileatus*); and antenna with fewer flagellomeres: 31 in *Aleiodes onyx* sp. n. vs. 34 or more in *Aleiodes atripileatus*. Other diagnostic characters fo *Aleiodes onyx* sp. n. are the occipital carina interrupted on vertex, the very short ovipositor, about as long as hind 3^rd^ tarsomere, metasoma stout, T1 about 0.9× as long as its apical width and slightly wider than mesosoma. The host mummy is similar to that of *Aleiodes atripileatus*, but the anal prolegs are not posteriorly extended in mummies made by *Aleiodes onyx* sp. n. The base color of the mummy is a metallic graphite-like tone, as opposed to opaque black in *Aleiodes atripileatus* mummies. *Aleiodes onyx* sp. n. is the first *Aleiodes* species known from Ecuador to be reared from Zygaenidae caterpillars.

#### Etymology.

From the Greek, the word *onyx* means “nail”. It is the name of a rock, used in adornments since ancient times, with several colors, being the black ones the most appreciated. The name is a reference for the main black color of this parasitoid mummy, which resembles the color of the black onyx rocks.

**Figures 92–95.**
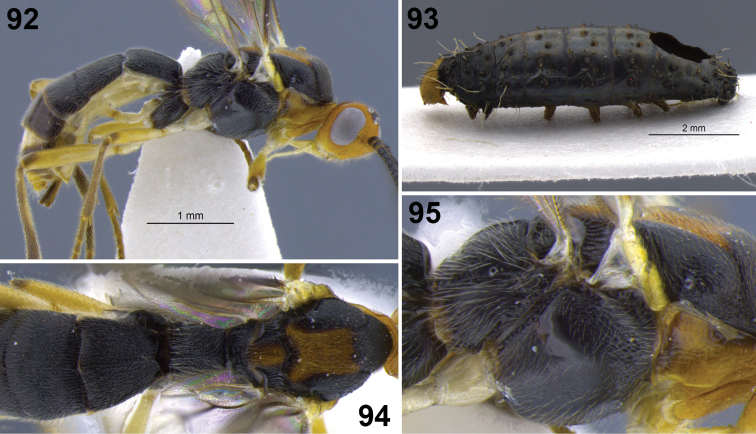
*Aleiodes onyx* sp. n. **92** habitus **93** host mummy after parasitoid emergence **94** mesopleuron **95** mesosoma and metasomal terga 1–2, dorsal.

### Aleiodes shakirae sp. n.

http://zoobank.org/DD5650C8-3A93-4088-88A2-300A07CE2557

http://species-id.net/wiki/Aleiodes_shakirae

**Figures 96–100.**
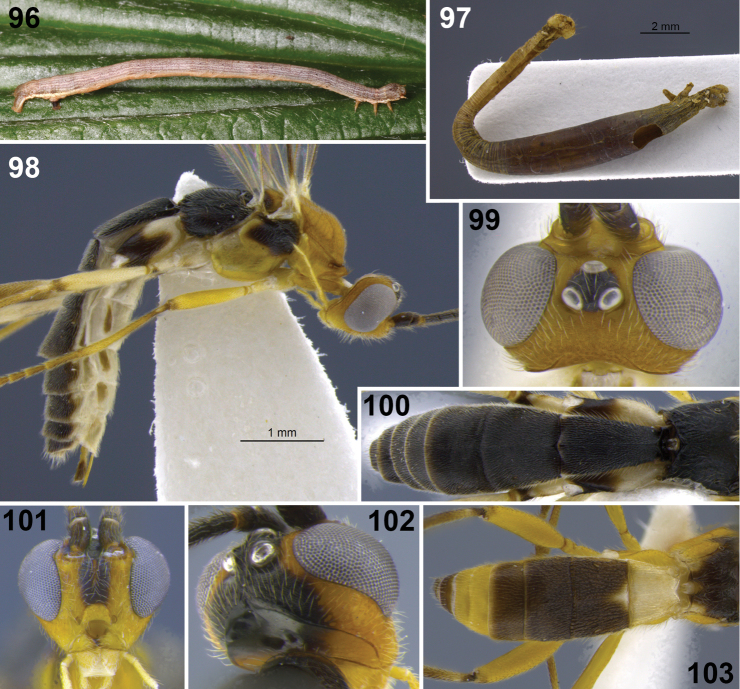
*Aleiodes shakirae* sp. n. **96** host larva (Geometridae) **97** host mummy after parasitoid emergence **98** habitus **99** head, dorsal **100** metasoma, dorsal. **Figures 101–103.** *Aleiodes speciosus* Townsend. **101** head frontal **102** head, occiput and vertex **103** metasoma, dorsal.

Figures 96–100 , 120

#### Description of holotype.

Female (holotype). Body length 6.0 mm; antenna length 7.4 mm; fore wing length 5.7 mm.

Color. Head yellow, ocellar triangle dark brown; antenna dark brown–black, scapus lighter dorsally; mesosoma yellow, anterior corner of mesopleuron, metanotum and part of lunules, propodeum and metapleuron dark brown–black, mesopleuron light yellow–whitish at broader with metapeluron but blackish in immediate border; fore and mid legs whitish at coxa, trochanter and trochantellus, darkening toward apex from light yellow to honey brown; hind leg: 1/2 basal coxa white, 1/2 apical black, trochanter white, trochantellus black with ventral white stripe, femur and tibia whitish with infuscate stains mid laterally and at apex, tibial spurs and telotarsi honey brown, remaining tarsi brown. Metasomal terga black, but T4–T7 white laterally; metasoma ventrally white; ovipositor sheaths dark brown on apical half, basal half whitish. Wings hyaline; stigma brown with central area lighter; most veins dark brown; vein C+SC+R black (extreme base whitish) connecting to a black parastigma with whitish central spot.

Head. Antenna with 47 antennomeres, flagellomeres roughly 2.0× as long as wide, apical flagellomere with long “bottle-nipple”-shaped apex; malar space as long as basal width of mandible, and approximately 1/4 eye height; in dorsal view eyes 3.4× temples; occipital carina incomplete dorsally, curving toward lateral ocelli, laterally complete and meeting hypostomal carina; oral space small and circular, maximum width slightly equal to basal width of mandible; clypeus not swollen; ocellus moderate, ocell–ocular distance short, about 0.45× diameter of lateral ocellus; maxillary palp not swollen; head surface sculpturing finely granulate, occiput smooth and shining; higher face with some wrinkles just bellow toruli.

Mesosoma. Sculpturing finely granulate; propodeum with mid-longitudinal carina complete; notauli weak, shallow and smooth; posterior margin of mesoscutum with complete carina; scutellar sulcus with median carina plus two pairs of lateral carina.

Wings. Fore wing: stigma 4.5× longer than high; vein r 1.27× vein 2RS, 1.2× vein RS+Mb, and as long as vein m-cu; vein 3RSa about 0.44× vein 3RSb, and 0.8× vein 2M; vein 1CUa 1.6× vein 1cu-a; vein 1CUb 2.2× vein 1CUa; vein 1M strongly curved at basal portion. Hind wing: m-cu virtually absent; vein M+CU 1.4× vein 1M; vein 1M 1.2× vein r-m; RS smoothly curved at middle; vein M dark brown, well pigmented; vein 2-1A absent.

Legs. Hind tibia without comb of modified setae; tarsal claws pectinate basally, with several very short and tight bristles, longer and sparser apically, wide gap between apical bristles and claw; hind basitarsus 3× longer than inner apical spur on hind tibia.

Metasoma. T1–T3 granular–rugose; remainder terga granular; mid longitudinal carina complete from T1 throughout T3; ovipositor sheaths about as long as hind tarsomere II; T1 long and narrow, T1 length 1.7× its apical width.

Paratypes variation. Body length 4.5–6.2 mm; antenna with 47–50 segments; legs color somewhat darker in few paratypes, and/or mid trochantellus laterally infuscate; scutellar sulcus with 3 to 5 carina; other color and proportions with only minimal variation.

Male. Antenna with 44 or 46 segments; ocelli larger, ocell–﻿cular distance 1/3 diameter of lateral ocellus; tergite 1 narrower, about 2× longer than apical width.

Mummy. Length 14.0–18.7 mm, dark reddish brown medially, light brown anteriorly and posteriorly, thorax narrow and wrinkled, mummy withered posteriorly behind exit hole, general long and narrow aspect, curved down- or sideward “V-shaped” to almost straight, exit hole irregular, located postero-dorsally, anterior to prolegs, but one specimen with ventral exit hole.

#### Type material.

Type-locality: ECUADOR, Napo Province, Yanayacu Biological Station, canopy Malaise trap on bamboo, S00°35.9', W77°53.4', 2163 m, cloud forest, July 10–16, 2010, S.R: Shaw col.

Type-specimen: Holotype female, point mounted. Top label: “ECUADOR: Napo Province / Yanayacu Biological Station / S00°35.9', W77°53.4’ 2163m / 10-16 June 2010, Scott R. Shaw / canopy Malaise trap, bamboo / NSF-BSI-07-17458 expedition”. (UWIM)

Paratypes 5 females and 6 males (UWIM), same data as holotype, except: 1♀, June 1–8, 2007, Malaise trap, SRS-00047; 3♀ and 6 males, reared from Geometridae larvae on *Chusquea scandens* (Poaceae): 1♂ June 2, 2006, YY-14220; 1♂ March 25, 2008, YY-29593; 1♂ August 26, 2009, YY-40478; 1♀ January 30, 2010, YY-44297; 1♂ July 29, 2010, YY-48878; 1♂ September 9, 2010, YY-50943; 1♂ November 16, 2010, YY-52497; 1♀, January 7, 2011, YY-52852; 1♀ March 10, 2011, YY-54224; 1♀ September 3, 2013, YY-78769.

#### Biology.

Reared from Geometridae caterpillar feeding on *Chusquea scandens* (Poaceae). Most host caterpillars were commonly named “palito café chusquea.” Considering associated caterpillar pictures, rearing information, and similar mummy morphology, it is likely that all the hosts were conspecific. Host caterpillars were collected in 2^nd^ and 3^rd^ instars. Time span, from pupation until adult emergence, varied from 2.5 up to 6 weeks. As the mummy dries, the middle section bends and dries in different ways, resulting in different but distinctive bent mummies.

#### Discussion.

*Aleiodes shakirae* sp. n. belongs to *circumscriptus*/*gastritor* species group. The “V-shaped” mummies from Geometridae and some color features resemble *Aleiodes townsendi* sp. n.; however, *Aleiodes shakirae* sp. n. differs from *Aleiodes townsendi* sp. n. in the ocell–ocular distance, which is about half the diameter of the lateral ocellus, but is nearly the same length as the lateral ocellus in *Aleiodes townsendi* sp. n. In *Aleiodes shakirae* sp. n. the metapeluron and metasoma are dorsally black (in *Aleiodes townsendi* sp. n. the metapleuron and T1 are mostly white with black markings), the hind coxa basally white and apically black (colors inverted in *Aleiodes townsendi* sp. n.), and hind tibia and tarsi are lighter in *Aleiodes shakirae* sp. n., the hind tibial spurs are yellowish to honey yellow (as compared with black in *Aleiodes townsendi*). The sculpturing of metasomal terga 1–3 in *Aleiodes shakirae* sp. n. is granular–rugose (rugose–striate in *Aleiodes townsendi* sp. n.), the metasoma is much slender in *Aleiodes shakirae* sp. n., petiole is 1.7–2.0 times longer than apical width (as compared with 1.2 times in *Aleiodes townsendi* sp. n.), and the hind wing m-cu is absent in *Aleiodes shakirae* sp. n. (present as a short pigmented stub in *Aleiodes townsendi* sp. n.). The host species “palito café chusquea” (Geometridae) is the same species attacked by *Aleiodes nubicola* sp. n. and *Aleiodes mirandae* sp. n.

#### Etymology.

This species is named after the famous Colombian singer Shakira. Since parasitism by this species causes the host caterpillar to bend and twist its abdomen in various ways, and Shakira is also famous for her belly-dancing, the name seems particulary appropriate for this species.

### Aleiodes speciosus

Townsend, 2009

http://species-id.net/wiki/Aleiodes_speciosus

Figures 101–103

#### Diagnosis.

Body length 4.9 mm; antenna with 42 segments; head color honey brown with black mark covering most of occiput, frons and vertex, and face medially; malar space 1.3× basal width of mandible; ocellus large, ocell–ocular distance 0.6× width of lateral ocellus; occipital carina shortly interrupted at vertex; mesopleuron mostly smooth, rugose on anter-dorsal corner; apex of hind tibia without comb of flattened setae; propodeum granulate anteriorly and rugose posteriorlly, longitudinal carina complete; metasomal tergite 1 white constrasting with most dark-brown–brow reminder terga, except terga 5–6 yellow.

#### Additional characters.

Last flagellomere with short pointed tip; mesoscutum with carina at posterior margin almost complete but weakly defined; scutellar sulcus shallow, with incomplete median carina; wings moderately infuscate; fore wing vein 1M moderately curved at base; hind wing vein 2-1A present, vein m-cu absent.

Type material examined. (UWIM)

#### Biology.

*Aleiodes speciosus* has been reared from unidentified Lepidoptera caterpillar on *Miconia* sp. (Melastomataceae).

#### Distribution.

Known only from the type locality, Camino a Loreto, Napo province, ECUADOR, at 1,383 meters elevation.

#### Discussion.

*Aleiodes speciosus* is known only by the male holotype. This species belongs to the *circumscriptus*/*gastritor* species-group. *Aleiodes speciosus* is similar to *Aleiodes kingmani* sp. n. mainly by color pattern, but also in having relatively large ocelli. These two species have a white metasomal tergite 1, contrasting with the reminder dark brown terga; however the mesosoma laterally and ventrally, and hind coxa of *Aleiodes speciosus* is mostly yellowish, compared with being almost entirely black in *Aleiodes kingmani* sp. n. The mummy produced by *Aleiodes speciosus*, although decapitated, is noticeably distinct from the ventrally bent “J-shaped” mummy of *Aleiodes kingmani* sp. n.

### Aleiodes stewarti sp. n.

http://zoobank.org/FE5FEE90-69ED-466B-88EA-7E9D52E58ADB

http://species-id.net/wiki/Aleiodes_stewarti

**Figures 104–106.**
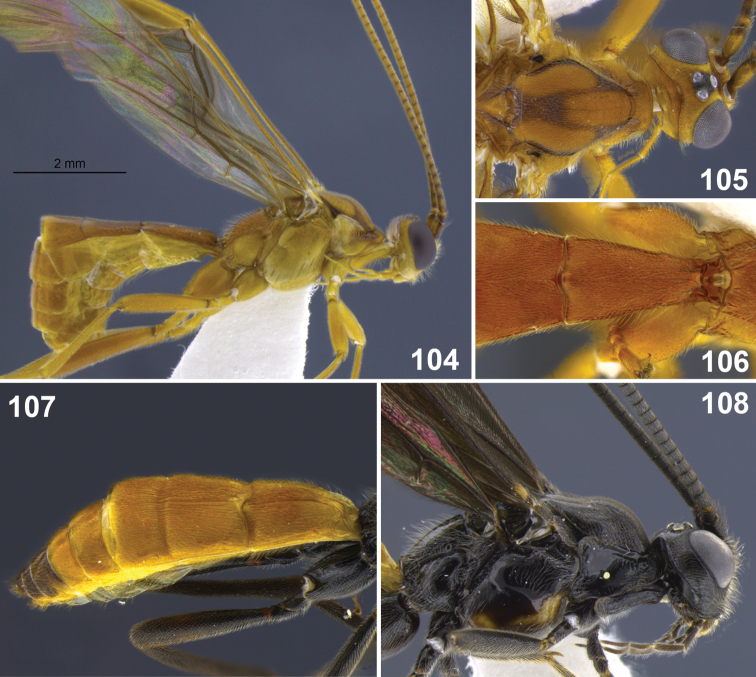
*Aleiodes stewarti* sp. n. **104** habitus **105** head and mesonotum, dorsal **106** metasomal tergite 1 **107, 108** *Aleiodes stilpnos* Townsend. 107, metosoma **108** head and mesosoma, lateral.

Figures 104–106 , 125

#### Description of holotype.

Female (holotype). Body length 9.1 mm; antenna length 9.4 mm; fore wing length 7.4 mm.

Color. Entire body yellowish brown to honey brown, darker dorsally; antenna basally dark brown, lightening gradually toward apex, apical 1/3 pale brown, scape light brown dorsally; face pale yellow, ocellar triangle black; lateral borders of mesoscutum, notauli and posterior depressed area brown; ovipositor sheaths mostly dark brown, basally whitish; wings slightly brown infuscate; veins dark brown except C+SC+R, stigma and R1 honey yellow.

Head. Antenna with 61 segments; flagellomeres about as long as wide, except apical 1/3 and basal 1/6 slightly longer than wide, apical flagellomere with small pointed apex; malar space as long as basal width of mandible, and 0.3× eye height; temple narrow, in dorsal view about eyes 5× longer than temples; occipital carina complete, reaching hypostomal carina; oral space small and circular, diameter about equal to basal width of mandible; clypeus weakly swollen; ocelli moderate, ocell–ocular distance about 1/2 diameter of lateral ocellus; face and gena rugose–costate, with mid-longitudinal ridge just bellow toruli, frons smooth and excavated, bordered by weak “W- shaped” carina; temples and vertex granulate.

Mesosoma. Sculpturing mostly granular; pronotum with median scrobiculate line; mesopleuron mostly shining granular–coriaceous, antero-dorsal corner rugose, central elevated area sharply defined and smooth, epicnemial carina complete; propodeum on posterior 1/3 smooth with longitudinal wrinkles, mid-longitudinal carina on anterior 2/3; metapleuron rugose posteriorly; notauli shallow and crenulate anteriorly, meeting rugose depressed area posteriorly; posterior margin of mesoscutum with carina interrupted laterally; scutellar sulcus with long median carina plus two pairs of incomplete lateral carina.

Wings. Fore wing: stigma 3.7× longer than high; vein r 0.55× length of 2RS, 0.45× length of m-cu, and 0.7× vein RS+Mb; vein 3RSa 0.46× vein 3RSb, and 0.88× vein 2M; vein 1CUa about 2× vein 1cu-a; 1CUb 1.6× length of 1CUa; vein 1M moderately curved at basal half. Hind wing: marginal cell widening toward apex, vein RS smoothly curved downward on base and well pigmented throughout; vein M+CU 1.3× longer than 1M; vein 1M about 1.4× longer than r-m; vein m-cu short, pigmented and non-tubular; vein 2-1A present and relatively long.

Legs. Tarsal claws strongly pectinate, with several relatively short bristles extending over the base of apical claw; basitarsus 3× longer than inner apical spur of hind tibia.

Metasoma. T1 and T2 striate, longitudinal carina complete on T1, incomplete on posterior ½ of T2; T3 with weak striation on anterior corners; remainder visible terga smooth; petiole long, very narrow basally, T1 1.7× longer than apical width; ovipositor sheaths about as long as hind tarsomere III.

Paratype variation. Essentially as holotype but antennomeres 60–64, scutellar sulcus with one or two pairs of lateral carina more or less defined and incomplete.

Male unknown.

#### Type material.

Type-locality: ECUADOR, Napo Province, Yanayacu Biological Station, Macucoloma trail, S00°35.9', W77°53.4', 2163 m, cloud forest, January 1–8, 2007, J. Simbaña col.

Type-specimen: Holotype female, point mounted. Top label: “ECUADOR: Napo Province / Yanayacu Biological Station / S00°35.9', W77°53.4', 2163m / 1-8 January 2007, J. Simbaña / Macucoloma trail, Malaise trap / NSF-BSI-07-17458, S.R. Shaw”. (UWIM)

Paratypes. 2♀, same data as holotype; 3♀, same data as holotype, except: 1♀, black light, May 15, 2011, S.R. Shaw col; 1♀, June–December 2011, canopy malaise trap (*Chusquea*); 1♀, September 5, 2005, malaise trap (Pumayacu ridge). (UWIM)

#### Discussion.

*Aleiodes stewarti* sp. n. belongs to the *pulchripes* species-group. In the key to New World species (S. Shaw et al. 1997), this species will run to *Aleiodes rossi* Marsh & Shaw, 1997. *Aleiodes stewarti* sp. n. can be distinguished from *Aleiodes rossi* by its mostly bronze color with a distinct color pattern on mesoscutum: notauli+posterior depressed area brown, all wing veins brown and antenna lightening apically (in *Aleiodes rossi* the body is entirely light yellow with all tarsi brown and apex of hind tibia black, the antenna is entirely brown with scape and pedicel yellowish). The bristles in tarsal claws in *Aleiodes stewarti* sp. n. are more numerous than in *Aleiodes rossi* and shortening apically, very similar to those on *Aleiodes cazieri* Marsh & Shaw, 1997 and *Aleiodes vaughani* Muesebeck, 1960. The first tergite of *Aleiodes stewarti* sp. n. is about 1.7× longer than its apical width, distinctly more slender than the previous described species in *pulchripes*-group, in which this proportion is around 1.0×, but in *Aleiodes colberti* sp. n. it is 1.5×. *Aleiodes stewarti* sp. n. and *Aleiodes colberti* sp. n. are the only two species in *Aleiodes pulchripes* species-group found so far from Yanayacu. *Aleiodes stewarti* sp. n. differs from *Aleiodes colberti* sp. n. in the antenna dark brown basally, gradually lightening toward pale brown apex (black with mid white band in *Aleiodes colberti* sp. n.), wings uniformly weakly infuscate (with dark band bellow stigma in *Aleiodes colberti* sp. n.), ocelli about 2× ocell–ocular distance (about 8× in *Aleiodes colberti* sp. n.); body mostly honey brown (reddish brown in *Aleiodes colberti* sp. n.), tarsal claw pectination with several short bristles extending to base of claw (in *Aleiodes colberti* sp. n. the pectination have less and larger bristles, and a distinct gap with claw base).

#### Comments.

Since *Aleiodes stewarti* sp. n. is described based on several females and *Aleiodes colberti* sp. n. is described based on one male, and considering the geographical distribution and sexual dimorphism in the group, there is a possibility of these two species are one single species with very extreme sexual dimorphism. However, we do not think that it is likely because none of the known species in *Aleiodes pulchripes* species-group, having both males and females described, exhibit anything close to such extreme variation, which compels us to maintain these two entities as distinct species.

#### Etymology.

This species is named after Jon Stewart (John Stuart Leibowitz), an American comedian, political satirist, writer, director, actor, and television host of *The Daily Show*.

### Aleiodes stilpnos

Townsend, 2009

http://species-id.net/wiki/Aleiodes_stilpnos

Figures 107 , 108 , 126

#### Diagnosis.

Body length 9.0 mm; antenna with 65 segments; head and mesosoma color mostly black, contrasting with yellowish orange metasoma; malar space 1.3× basal width of mandible; ocellus moderate, ocell–ocular distance slightly longer than width of lateral ocellus; occipital carina complete; mesopleuron smooth; apex of hind tibia without comb of flattened setae; propodeum mostly rugose, longitudinal carina complete.

#### Additional characters.

Last flagellomere with “bottle-nipple”-shaped tip; mesoscutum with carina at posterior margin present only in front of scutellar sulcus; scutellar sulcus with three strong and short carina; fore wing vein 1M slightly curved at base; hind wing vein 2-1A present and relatively long, vein m-cu absent.

Type material examined. (UWIM)

#### Biology.

*Aleiodes stilpnos* has been reared from unidentified Noctuidae caterpillar on *Polygonum punctatum* (Polygonaceae).

#### Distribution.

Known only from the type locality, YBS, Napo province, ECUADOR.

#### Discussion.

*Aleiodes stilpnos* is the only species treated in this work in the *albitibia* species-group. The *albitibia*-group is relatively rarely collected in the Neotropical region, with only two described – *Aleiodes stilpnos* from Ecuador and *Aleiodes fuscipennis* (Szépligeti, 1904) from Peru, Venezuela (Torres and Briceño 2005) and Chile (deposited at UWIM), and at least two undescribed species from Costa Rica. *Aleiodes stilpnos* differs from other species in Ecuador by the following characters: head, mesosoma and legs mostly black contrasting with light orange metasoma, and wings infuscate; mesopleuron central disc smooth and bare; tarsal claws strongly pectinate; and costate sculpturing on metasomal terga 1–3.

### Aleiodes townsendi sp. n.

http://zoobank.org/DE46E183-E9B7-4C43-9DCA-CCAEC880B559

http://species-id.net/wiki/Aleiodes_townsendi

Figures 109 , 110

#### Description of holotype.

Female (holotype). Body length 5.7 mm; antenna length 6.7 mm; fore wing length 5.1 mm.

Color. Head yellow, ocellar triangle dark brown; antenna dark brown–black, scapus lighter dorsally, extreme base of scapus and first flagellomere yellow; mesosoma yellow, anterior corner of mesopleuron, lunules, metanotum, propodeum and dorsal 1/4 of metapleuron dark brown, remainder metapleuron and mesopleuron, at border with metapleuron, white; latero-ventral mesopleuron with slightly lighter stripe; fore leg yellow, telotarsus brown; mid coxa, trochanter, trochantellus and femur basally white, remainder mid leg yellow, darkening toward apex, telotarsi and tibial spurs brown; hind leg: coxa black on 1/2 basal and white on 1/2 apical, trochanter and trochantellus black, but apical border of trochanter, and trochantellus ventral-apical 1/3 plus a small ventral spot white; femur black on basal 2/5 and at extreme apex, otherwise white; tibia and tarsi black, small white subbasal band on tibia. Metasoma white with black dorsal triangle beginning on central apex of T1 and covering most dorsal portion of subsequent terga; ovipositor sheaths dark brown on apical half, basal half whitish. Wings weakly infuscate; most veins and stigma dark brown; vein C+SC+R black (extreme base whitish) connecting to a black parastigma with whitish central spot.

Head. Antenna with 46 antennomeres, flagellomeres roughly 2.0× as long as wide, apical flagellomere with “bottle-nipple”-shaped apex; malar space as long as basal width of mandible, and 1/3 eye height; in dorsal view eye height 2.8× temple; occipital carina incomplete dorsally, curving toward lateral ocelli, laterally complete and meeting hypostomal carina; oral space small and circular, maximum width slightly smaller than basal width of mandible; clypeus not swollen; ocellus moderate, ocell–ocular distance 0.8× diameter of lateral ocellus; maxillary palp not swollen; head surface sculpturing finely granulate, occiput smooth and shining; higher face with some transverse wrinkles just bellow toruli.

Mesosoma. Sculpturing finely granulate; propodeum more coarsely granulate, with mid-longitudinal carina complete and some diverging wrinkles posteriorly; notauli weak, shallow and smooth; posterior margin of mesoscutum with complete carina; scutellar sulcus with median carina plus one pair of weak lateral carina.

Wings. Fore wing: stigma 4.5× longer than high; vein r 1.4× vein 2RS, 1.5× vein RS+Mb, and about as long as vein m-cu; vein 3RSa about 0.5× vein 3RSb, and 0.85× vein 2M; vein 1CUa 1.7× vein 1cu-a; vein 1CUb 1.7× vein 1CUa; vein 1M strongly curved at basal portion. Hind wing: m-cu indicated as short pigmented not tubular vein interstitial to vein r-m; vein M+CU 1.3× vein 1M; vein 1M 1.4× vein r-m; RS smoothly curved at middle; vein M dark brown, well pigmented; vein 2-1A absent.

Legs. Hind tibia without comb of modified setae; tarsal claw pectinate basally, with several very short and tight bristles, longer and more sparse apically, wide gap between apical claw and basal pecitnation; hind basitarsus about 3× longer than inner apical spur on hind tibia.

Metasoma. T1–T3 rugose–striate; remainder terga coriaceous; mid longitudinal carina complete from T1 throughout T3; ovipositor sheaths about as long as hind tarsomere II; T1 length 1.2× its apical width.

Paratype variation. Antenna broken, otherwise essentially as holotype.

Male unknown.

Mummy. Length 17.0 mm, entire mummy mottled with gray and brownish, thorax narrow and wrinkled, mummy withered posteriorly behind exit hole, mummy aspect long and narrow, curved upward “V-shaped”, exit hole irregular, located postero-dorsally, anterior to prolegs.

#### Type material.

Type-locality: ECUADOR, Napo Province, Yanayacu Biological Station, YY-44074, S00°35.9', W77°53.4', 2163 m, cloud forest, January 23, 2010.

Type-specimen: Holotype female and mummy, point mounted separately. Top label: “ECUADOR: Napo Province / Yanayacu Biological Station / S00°35.9', W77°53.4’ 2163m / 23 December 2010, Yanayacu Road / YY-44074, ex. Geometridae”. (UWIM)

Paratype, female, same data as holotype, except: August 3, 2005, Malaise trap, A. Townsend col. (UWIM)

#### Biology.

Reared from a Geometridae caterpillar (no common name) on *Dendrophobium lloense* (Asteraceae). Time span from host mummification until adult emergence was 20 days.

#### Discussion.

*Aleiodes townsendi* sp. n. belongs to the *circumscriptus*/*gastritor* species group. It is similar to *Aleiodes shakirae* sp. n. because of the presence of a strongly curved vein 1M in the fore wing, some similar color patterns, and the elongate and curved “V-shaped” mummy. It differs from *Aleiodes shakirae* sp. n. by having the metasomal tergite 1 mostly white with a small mid-apical black spot and about as long as apical width, entirely black or dark brown and about 2× longer than apical width in *Aleiodes shakirae* sp. n. The metapleuron in *Aleiodes townsendi* sp. n. is bicolored, black and white, while entirely black in *Aleiodes shakirae* sp. n. *Aleiodes townsendi* sp. n. hind coxa is black basally and apically white, compared with the inverse color pattern in *Aleiodes shakirae* sp. n., and the wings are moderately infuscate, while hyaline in *Aleiodes shakirae* sp. n. (additional features are cited in discussion section for *Aleiodes shakirae* sp. n.). The ocelli in *Aleiodes townsendi* sp. n. are relatively small, but its large eyes, almost 3× longer than temple in dorsal view, make the ocell–ocular distance shorter, the width of lateral ocellus being roughly equal to ocell–ocular distance.

#### Etymology.

This species is named after Andrew Townsend, collector of one of the type specimens, for his contributions to the knowledge of the Ecuadorian Braconidae fauna.

**Figures 109–113.**
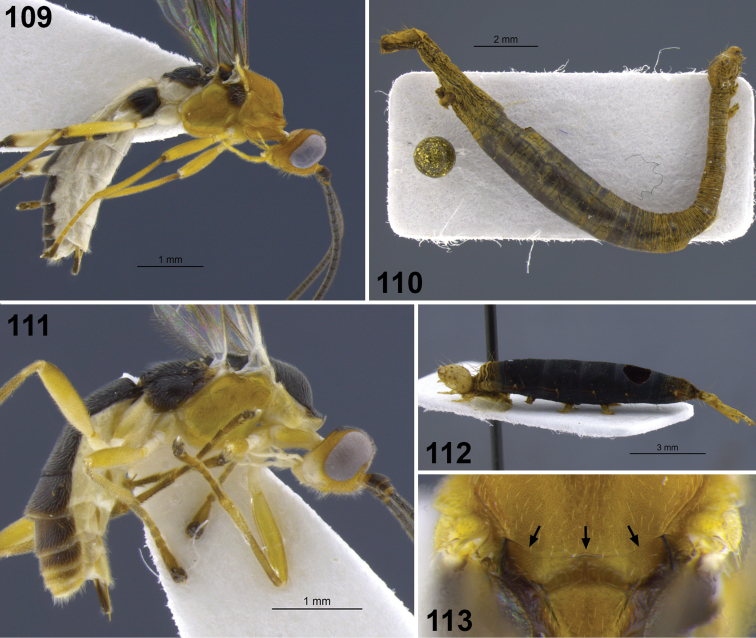
**109, 110** *Aleiodes townsendi* sp. n. **109** habitus **110** host mummy after parasitoid emergence. **111–113** *Aleiodes tzantza* sp. n. **111** habitus **112** host mummy **113** scutellar sulcus, arrows indicating carina at posterior margin of mesoscutum.

### Aleiodes tzantza sp. n.

http://zoobank.org/9351F3F9-1AE2-4940-BC7B-CF592B3DBD2A

http://species-id.net/wiki/Aleiodes_tzantza

Figures 111–113

#### Description of holotype.

Female (holotype). Body length 5.0 mm; antenna length 5.8 mm; fore wing length 5.0 mm.

Color. Dorsally dark brown to black, except head; laterally and ventrally mostly yellowish to whitish. Head honey yellow; mandibles, cheeks, and palp whitish, but teeth brown; black stain dorsally, from ocellar triangle through occiput mid-dorsally. Mesosoma black dorsally; pronotum and propleuron whitish, but mid-dorsally pronotum dark brown; mesopleuron honey yellow; metapleuron black. Legs mostly yellowish, darkening toward apex, from whitish coxa to light brown, tarsi and dark brown claws. Metasoma dark brown dorsally, apex lightening behind T3, ventrally whitish; ovipositor sheaths dark brown. Wings weakly infuscate, veins brown.

Head. Antenna with 42 segments, flagellomeres roughly 2.0× as long as wide, apical flagellomere with short “bottle-nipple”-shaped apex; malar space moderate, length 1.3× basal width of mandible, and 0.4× eye height; in dorsal view eye height 2.6× temple; occipital carina incomplete dorsally, otherwise well defined, reaching hypostomal carina; oral space small and circular, maximum width slightly smaller than basal width of mandible; clypeus not swollen; ocell–ocular distance 0.6× diameter of lateral ocellus; maxillary palp not swollen. Head surface sculpturing finely granulate, vertex sculpturing coarser, face with irregular transverse rugosity concentrated near toruli, occiput smooth and shining.

Mesosoma. Sculpturing mostly finely granulate; propodeum rugose–granulate, with complete mid-longitudinal carina; notauli anteriorly, narrow, posteriorly indicated by carina and meeting in front of scutellar sulcus; mesiscutum mid-posterior area depressed and granular, posterior margin of mesoscutum bordered with complete carina; scutellar sulcus with short median carina.

Wings. Fore wing: stigma 3.8× longer than high; vein r 0.9× vein 2RS, as long as vein RS+Mb, and 0.7× as long as vein m-cu; vein 3RSa about 0.45× vein 3RSb, and as long as vein 2M; vein 1CUa 2× vein 1cu-a; vein 1CUb 2.2× vein 1CUa; vein 1M evenly slightly curved. Hind wing: m-cu indicated as short fold, interstitial to r-m; M+CU 1.3× 1M; vein 1M 1.45× vein r-m; RS smoothly curved at middle; vein 2-1A present as a short stub.

Legs. Hind tibia without comb of modified setae; tarsal claw simple, with a comb of thin bristles medially; hind basitarsus about 4× longer than inner apical spur on hind tibia.

Metasoma. T1–T3 rugose–striated with granulate background; remainder terga granulate; mid longitudinal carina complete from T1 throughout T2, absent on T3; ovipositor sheaths about as long as hind tarsomere II; T1 1.2× longer than its apical width.

Paratype variation. Very similar to holotype, but with 41 antennomeres.

Male unknown.

Mummy. Length 9.0–10.0 mm, abdomen black, head, thorax, legs, and prolegs light pale brown, thorax compact and wrinkled, anal prolegs largely extended posteriorly, glue hole located ventrally on the thorax, exit hole irregular, located postero-dorsally, posterior to hind abdominal prolegs.

#### Type material.

Type-locality: ECUADOR, Napo Province, Yanayacu Biological Station, YY-48320, S00°35.9', W77°53.4', 2163 m, cloud forest, June 5, 2010.

Type-specimen: Holotype female and mummy, point mounted separately. Top label: “ECUADOR: Napo Prov. / Yanayacu Biological Station / S00°35.9', W77°53.4’ 2163m / REARED / (hand written) 48320”; back (hand written): “May 2010 / 5-May-2010”. (UWIM)

Paratype, female, same locality as holotype, August 14, 2005, reared YY-5189. (UWIM)

#### Biology.

The holotype was reared from a mummified larva collected on *Palicourea ulloana* (Rubiaceae). The sampling date is listed in the database as May 12, 2010, and the adult emergence date is listed as May 5, 2010, so clearly one of these dates must be incorrect. Since this caterpillar record is nested within a large group of other caterpillar records also collected on May 12, 2010, it seems most likely that the emergence date was recorded incorrectly. Since the pupation date is assigned as May 18, 2010, this also corroborates that the emergence date could not possibly have been May 5, 2010. It seems most likely that the emergence month was recorded incorrectly and perhaps the actual emergence date was June 5, 2010. The holotype host is probably a Noctuidae due to similarity with the paratype mummy, which is possibly conspecific, identified as a Noctuidae. The host plant for the paratype is unknown, and it is not possible to determine if the holotype host caterpillar fed or not on *Palicourea ulloana*.

#### Discussion.

This species belongs to *circumscriptus*/*gastritor* species group. *Aleiodes tzantza* sp. n. is similar to *Aleiodes atripileatus*, from which it can be distinguished by the larger ocelli: ocell–ocular distance about half diameter of lateral ocelli (1.5 to 1.7× in *Aleiodes atripileatus*), and the color of mesopleuron entirely yellow (dark brown at least dorsally in *Aleiodes atripileatus*). *Aleiodes tzantza* sp. n. mummies are very similar to those of *Aleiodes atripileatus*, but the projecting anal prolegs are much longer in this species, and the body is distinctly longer. Its short ocell–ocullar distance is similar to *Aleiodes speciosus*, from which it differs by having the mesopleuron surface granular (mostly smooth with anterior quarter rugose in *Aleiodes speciosus*) and entirely honey yellow (anterior 1/4 black in *Aleiodes speciosus*), pronotum whitish laterally (mostly dark brown in *Aleiodes speciosus*), and metasomal terga all dark brown (first tergite white in *Aleiodes speciosus*).

#### Etymology.

“Tzantza” is the Shuar word for the ritual of reducing heads by a mummification process used by the Shuar, a people native from the current Ecuadorian Amazon territory, resulting in a shrunken mummy as the ones produced by the *Aleiodes* species.

### Aleiodes yanayacu sp. n.

http://zoobank.org/FEA654AE-3EF8-444B-839B-CE6B241EBED9

http://species-id.net/wiki/Aleiodes_yanayacu

Figures 114–118

#### Description of holotype.

Male (holotype). Body length 4.4 mm; antenna length 5.5 mm; fore wing length 4.5 mm.

Color. Mostly black. Propodeum with mid-apical white mark; metapleuron mostly whitish, but brown dorsally. Metasoma with T1 and T2 pale light yellow, reminder terga dark brown. Fore legs mostly dark brown, trochanter whitish with brown mark on inner side; mid legs mostly dark brown, coxa, trochanter and trochantellus yellowish, femur mostly light brown; hind legs yellowish basally, apex of femur with brown stain dorsally, tibia and tarsi dark brown except for whitish stain covering basal half of inner side of tibia. Wings infuscate with dark brown veins and stigma.

Head. Antenna with 42 antennomeres, flagellomeres roughly 2.0× as long as wide, apical flagellomere with pointed tip; malar space about as long as basal width of mandible, and 0.4× eye height; in dorsal view eye 1.4× temple; occipital carina incomplete, interrupted at vertex, well defined laterally and meeting hypostomal carina; oral space small and circular, maximum width roughly equal to basal width of mandible; clypeus slightly swollen; ocell–ocular distance about 1.3× diameter of lateral ocellus; maxillary palp not swollen; head surface sculpturing granulate, occiput smooth and shining; frons slightly excavated, shining coriaceous, sculpturing concentrically arranged; face with some transverse rugae medially, and a short but well defined mid-longitudinal carina higher on face, carina extending dorsally between toruli.

Mesosoma. Sculpturing finely granulate; pronotum foveate; mesopleuron central disc smooth and bare, antero-dorsal corner rugose; metapleuron smooth; propodeum coarsely granular with complete mid-longitudinal carina and some longitudinal rugosity posteriorly; notauli well defined and crenulate anteriorly, barely defined but traceable posteriorly, meeting a rugose area; posterior margin of mesoscutum bordered with complete carina; scutellar sulcus with median carina plus two pairs of incomplete lateral carina.

Wings. Fore wing: stigma about 3.3× longer than high; second submarginal cell relatively large; vein r 0.87× vein 2RS, 1.4× as long as vein RS+Mb, and 0.8× as long as vein m-cu; vein 3RSa about 0.5× vein 3RSb, and 0.9× vein 2M; vein 1CUa 2.8× vein 1cu-a; vein 1CUb about as long as vein 1CUa; vein 1M slightly curved at base. Hind wing: m-cu absent; M+CU as long as 1M; vein 1M 1.7× vein r-m; vein RS smoothly curved at middle; vein M well pigmented; vein 2-1A present, short.

Legs. Hind tibia without apical comb of modified setae; tarsal claw simple, not pectinate, with a comb of relatively long thin setae basally; hind tibial spurs relatively short, about 1/4 basitarsus length. Hind coxa smooth.

Metasoma. T1, T2 and basal 1/2 of T3 costate, costa widely spaced over underlying granulation, sculpturing weaker at T3, longitudinal carina present along this sculpturing; remainder T3 and apical terga weakly shining coriaceous; T1 length about as long as its apical width.

Female unknown.

Mummy. Length 7.5 mm, head missing, black, exit hole irregular, located postero-dorsally, posterior to hind abdominal prolegs.

#### Type material.

Type-locality: ECUADOR, Napo Province, Yanayacu Biological Station, S00°35.9', W77°53.4', 2163 m, cloud forest, 18 July 2013, reared from caterpillar: YY-75311.

Type-specimen: Holotype male, point mounted. Top label: “ECUADOR: Prov. Napo / Yanayacu Biological Station / S00°35.9', W77°53.4’ 2163m / REARED: Jun 2013 / (hand written) 75311. Back: “18-Jul-2013”. (UWIM)

#### Biology.

Reared from a Geometridae species, feeding on *Phenax rugosus* (Urticaceae). Three weeks elapsed from mummification until adult emergence.

#### Discussion.

*Aleiodes yanayacu* sp. n. belongs to *circumscriptus*/*gastritor* species-group. This species is similar to *Aleiodes mirandae* sp. n. and *Aleiodes napo* sp. n. in the mostly smooth mesopleuron, and the occipital carina, interrupted at vertex. *Aleiodes yanayacu* sp. n. differs from both species in having a slightly larger ocellus, the ocell–ocular distance is about 1.3× the diameter of lateral ocellus, while in *Aleiodes mirandae* sp. n. and *Aleiodes napo* sp. n. it is about 2.0×. The hind wing vein M+CU is about as long as vein 1M in *Aleiodes yanayacu* sp. n., but distinctly shorter in *Aleiodes mirandae* sp. n. and *Aleiodes napo* sp. n. The sculpturing of metasomal terga 1 and 2 is also distinctive in *Aleiodes yanayacu* sp. n., with widely spaced costa, as compared with the finely rugose–costate sculpturing in *Aleiodes mirandae* sp. n. and *Aleiodes napo* sp. n. The metasomal tegite 2 is entirely withish in *Aleiodes yanayacu* sp. n. (black and white in *Aleiodes mirandae* sp. n. and *Aleiodes napo* sp. n.) and the hind coxa yellowish (black *Aleiodes mirandae* sp. n. and *Aleiodes napo* sp. n.).

#### Etymology.

The species is named after the sampling and rearing location, theYanayacu Biological Station.

**Figures 114–118.**
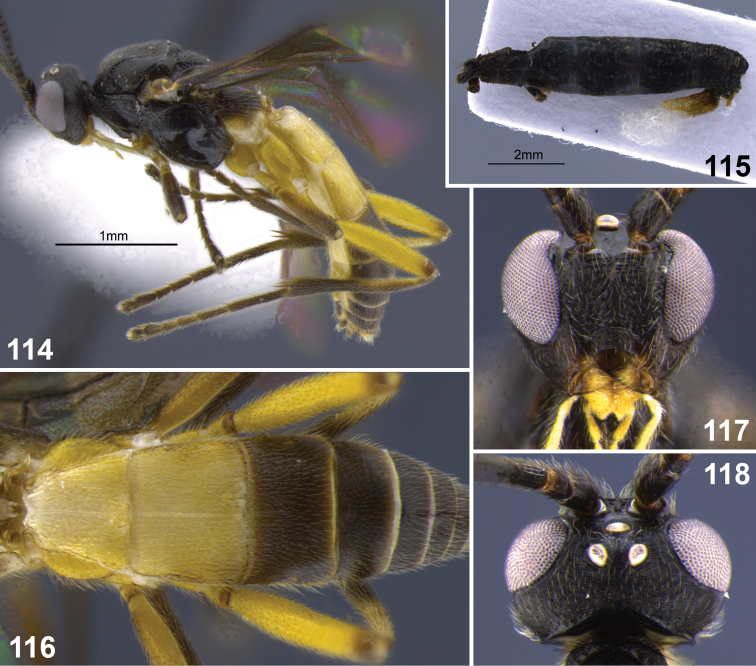
*Aleiodes yanayacu* sp. n. **114** habitus **115** host mummy after parasitoid emergence **116** metasoma, dorsal **117** head, frontal **118** head, dorsal.

**Figures 119–120.**
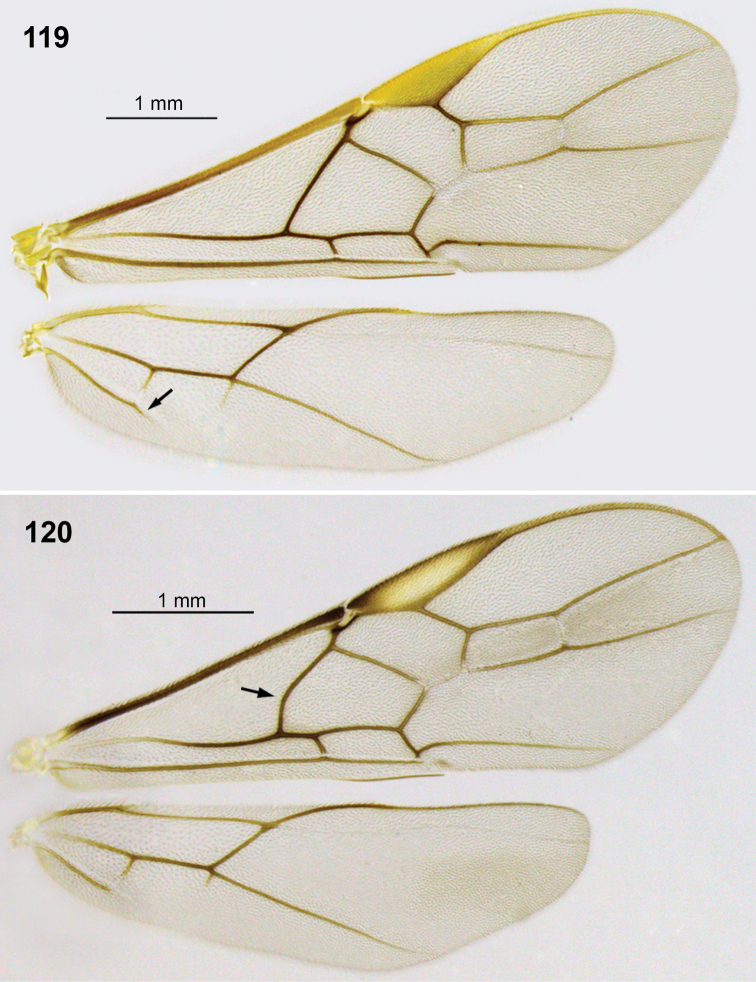
Wings. **119** *Aleiodes luteosicarius* sp. n., arrow = vein 2-1A, hind wing, fore wing **120** *Aleiodes shakirae* sp. n., arrow = vein 1M.

**Figures 121–126.**
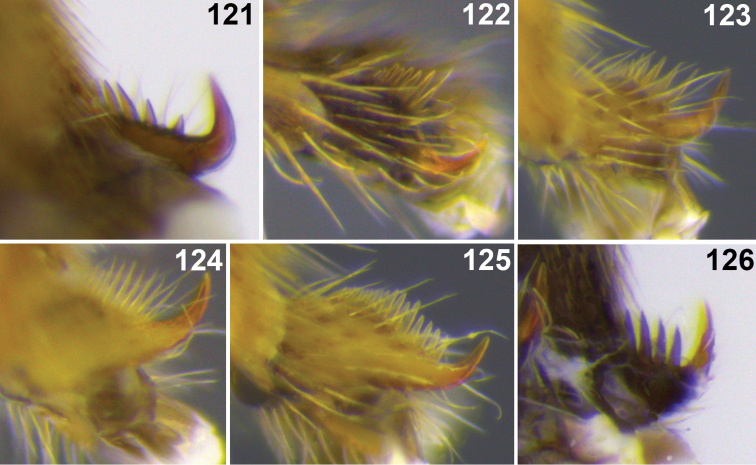
Tarsal claws. **121** *Aleiodes colberti* sp. n. **122** *Aleiodes dyeri* sp. n. **123** *Aleiodes falloni* sp. n. **124** *Aleiodes luteosicarius* sp. n. **125** *Aleiodes stewarti* sp. n. **126** *Aleiodes stilpnos* Townsend.

## Supplementary Material

XML Treatment for Aleiodes aclydis

XML Treatment for Aleiodes albidactyl

XML Treatment for Aleiodes albigena

XML Treatment for Aleiodes albiterminus

XML Treatment for Aleiodes albiviria

XML Treatment for Aleiodes arbitrium

XML Treatment for Aleiodes atripileatus

XML Treatment for Aleiodes bimaculatus

XML Treatment for Aleiodes cacuangoi

XML Treatment for Aleiodes capillosus

XML Treatment for Aleiodes colberti

XML Treatment for Aleiodes delicatus

XML Treatment for Aleiodes dyeri

XML Treatment for Aleiodes elleni

XML Treatment for Aleiodes falloni

XML Treatment for Aleiodes frosti

XML Treatment for Aleiodes greeneyi

XML Treatment for Aleiodes kingmani

XML Treatment for Aleiodes longikeros

XML Treatment for Aleiodes luteosicarius

XML Treatment for Aleiodes marilynae

XML Treatment for Aleiodes mirandae

XML Treatment for Aleiodes napo

XML Treatment for Aleiodes nebulosus

XML Treatment for Aleiodes nubicola

XML Treatment for Aleiodes onyx

XML Treatment for Aleiodes shakirae

XML Treatment for Aleiodes speciosus

XML Treatment for Aleiodes stewarti

XML Treatment for Aleiodes stilpnos

XML Treatment for Aleiodes townsendi

XML Treatment for Aleiodes tzantza

XML Treatment for Aleiodes yanayacu
